# Small RNA expression from viruses, bacteria and human miRNAs in colon cancer tissue and its association with microsatellite instability and tumor location

**DOI:** 10.1186/s12885-019-5330-0

**Published:** 2019-02-20

**Authors:** Robin Mjelle, Wenche Sjursen, Liv Thommesen, Pål Sætrom, Eva Hofsli

**Affiliations:** 1 Department of Clinical and Molecular Medicine, Norwegian University of Science and Technology, NTNU, Erling Skjalgssons gt 1, 7030 Trondheim, Norway; 2 Department of Medical Genetics, St Olavs Hospital, Trondheim Norway, Erling Skjalgssons gt 1, 7030 Trondheim, Norway; 3 Department of Biomedical Laboratory Science, Norwegian University of Science and Technology, NTNU, 7491 Trondheim, Norway; 4 Department of Computer and Information Science, Norwegian University of Science and Technology, NTNU, Sem Sælandsvei 9, 7491 Trondheim, Norway; 5 Bioinformatics core facility-BioCore, Norwegian University of Science and Technology, NTNU, 7491 Trondheim, Norway; 6 K.G. Jebsen Center for Genetic Epidemiology, Norwegian University of Science and Technology, NTNU, 7491 Trondheim, Norway; 7 The Cancer Clinic, St. Olav’s Hospital, Trondheim University Hospital, Trondheim, Norway

**Keywords:** miRNA, isomiR, ncRNA, sRNA, Epstein-Barr virus, Fusobacterium nucleatum, Colon cancer, High throughput sequencing

## Abstract

**Abstract:**

**Background:**

MicroRNAs (miRNA) and other small RNAs are frequently dysregulated in cancer and are promising biomarkers for colon cancer. Here we profile human, virus and bacteria small RNAs in normal and tumor tissue from early stage colon cancer and correlate the expression with clinical parameters.

**Methods:**

Small RNAs from colon cancer tissue and adjacent normal mucosa of 48 patients were sequenced using Illumina high-throughput sequencing. Clinical parameters were correlated with the small RNA expression data using linear models. We performed a meta-analysis by comparing publicly available small RNA sequencing datasets with our original sequencing data to confirm the main findings.

**Results:**

We identified 331 differentially expressed miRNAs between tumor and normal samples. We found that the major changes in miRNA expression between left and right colon are due to miRNAs located within the Hox-developmental genes, including miR-10b, miR-196b and miR-615. Further, we identified new miRNAs associated with microsatellite instability (MSI), including miR-335, miR-26 and miR-625. We performed a meta-analysis on all publicly available miRNA-seq datasets and identified 117 common miRNAs that were differentially expressed between tumor and normal tissue. The miRNAs miR-135b and miR-31 were the most significant upregulated miRNA in tumor across all datasets. The miRNA miR-133a was the most strongly downregulated miRNA in our dataset and also showed consistent downregulation in the other datasets. The miRNAs associated with MSI and tumor location in our data showed similar changes in the other datasets. Finally, we show that small RNAs from Epstein-Barr virus and Fusobacterium nucleatum are differentially expressed between tumor and normal adjacent tissue.

**Conclusions:**

Small RNA profiling in colon cancer tissue revealed novel RNAs associated with MSI and tumor location. We show that Fusobacterium nucleatum are detectable at the RNA-level in colon tissue, and that both Fusobacterium nucleatum and Epstein-Barr virus separate tumor and normal tissue.

**Electronic supplementary material:**

The online version of this article (10.1186/s12885-019-5330-0) contains supplementary material, which is available to authorized users.

## Background

Both host small RNAs (sRNAs) and bacterium and virus pathogens are implicated in the pathogenesis of several human cancers, including colorectal cancer (CRC). MicroRNAs (miRNAs), which is one of the most studied classes of sRNAs, have altered expression profiles in CRC and these profiles differ between CRC subtypes. For example, miRNAs correlate with microsatellite instability (MSI) status [1, 2], tumor location [3, 4], BRAF and KRAS mutation [5, 6] and tumor stage [7]. It is important to understand the underlying molecular mechanisms of CRC heterogeneity, since these mechanisms influence treatment decision-making.

Many studies have investigated the role of microorganisms in CRC, mostly focusing on the gut microbiota (e.g. [8, 9]). Analyses by 16S ribosomal DNA sequencing or real-time PCR have demonstrated enrichment of bacteria, including the oral bacterium *Fusobacterium nucleatum* (*F. nucleatum*), in tumor compared to adjacent normal tissue [10–14]. *F. nucleatum* and other bacteria can modulate the microenvironment to cause inflammation and increase proliferation [15, 16].

In contrast to bacteria, the role of viral pathogens in CRC is less explored. Human Papilloma Virus (HPV) is detected in CRC tumors in some studies; however, no consensus is reached regarding its role, and the frequency of detection varies largely between studies [17]. The role of HPV is more evident for anal cancer, in which HPV is detected in the majority of the malignancies [18]. Epstein-Barr virus (EBV) is detected in CRC tumors, also with varying frequency [19–23]. Other viruses such as John Cunningham virus and Human Cytomegalovirus have been detected in CRC tissue and are suggested to have a role in enhancing cell proliferation [24, 25].

Here we use small RNA sequencing to analyze 48 early stage colon tumors and 48 matched adjacent normal controls. By combining our data with small RNA sequencing data from The Cancer Genome Atlas (TCGA) and three other cohorts [7, 26, 27], we provide a comprehensive view of canonical miRNAs, miRNA variants (isomiRs), and other small RNAs that differ between tumor and normal samples and between clinical subtypes of the tumors. Furthermore, we show that a large proportion of the sequencing reads that did not match any region in the human genome mapped to virus and bacterial species. Among the most prominent species were *Fusobacterium nucleatum* and *Epstein-Barr-virus*, which both showed over-expression in tumors compared with normal adjacent colon. Our results show that small RNAs can provide comprehensive and consistent data for early detection and subtyping of colon cancer.

## Methods

### Patient samples

The current study was performed as a retrospective study on samples from St. Olavs Hospital biobank. Samples from patients with newly diagnosed primary colon cancer from two hospitals in Norway (St Olavs Hospital, Trondheim and Hamar Hospital, Hamar) were included, as described in [28]. Tumor tissue from colon (coecum to sigmoideum, not rectum) and adjacent normal mucosa from 48 patients with tumors detected at early stages I-II (no lymph nodes or distant metastases), were included in the present study. All patients gave written consent to attend the study.

### Tumor molecular analyses and clinical parameters

Status of MSI, BRAF mutation (V600E) and methylation of MLH1 promoter in tumor were analyzed as described [28]. KRAS mutation analyses were performed in DNA isolated from tumor tissue by sequencing of exon 2 and 3, to identify activating mutations in codon 12, 13 and 61. Primers for exon 2 were 5′-TGGTGGAGTATTTGATAGTGT-3′ (forward) and 5’-CCTCTATTGTTGGATCATATTC-3′ (reverse) and primers for exon 3 were 5’-GGTGCACTGTAATAATCCAGACTGTGT-3′ (forward) and 5’-TGCATGGCATTAGCAAAGAC-3′ (reverse).

Right-sided colon was defined as location 1–5 (appendix, coecum, ascendens and right flexure and transversum). Left-sided colon was defined as location 6–10 (left flexure, descendens, sigmoid, rectosigmoid and rectum).

### RNA isolation

Total RNA was isolated from tissue samples by using miRVana RNA isolation kit (ThermoFisher). RIN values were measured using Eukaryote total RNA assay on the Agilent 2100. RIN > 9 was regarded as high quality and sufficient for sequencing.

### Preparation of cDNA library for small RNA sequencing

Small RNA sample preparation was performed using the TruSeq small RNA protocol (Illumina) according to the manufacturer’s instructions, using 1μg RNA and 13 PCR cycles. During the 3′ ligation step, 10 synthetic calibrator RNAs were added to the master mix to serve as internal controls. The miRNA fragments were sequenced on the Illumina HiSeq 2500 system using 50 base pair single read.

### Mutation count analysis

Masked somatic mutations for TCGA colon adenocarcinomas were downloaded from the national cancer institute’s genome data commons. A linear model correlating somatic mutation counts in exomes with presence of loss-of-function and missense mutations in one or more of the MMR genes MSH2, MSH6, MLH1, and PMS2 in TCGA were created using the *lm* function in R in the following form: Count ~ LossOfFunction + Missense. Here, “Count” is the log2 number of mutations, and “LossOfFunction” and “Missense” is presence of loss-of-function and missense mutation, respectively. The alternative model including miR-155 expression had the following form: Count ~ LossOfFunction + Missense + miR155, where miR155 was log2 normalized miR-155-5p expression. The *p*-values represents the p-values from the F-statistics in the linear model in R.

### Reverse transcription quantitative PCR (RT-qPCR)

We used the Taqman technology for RT-qPCR analyses (ThermoFisher). Custom probes were designed using Custom TaqMan® Small RNA Assays. We used the following probes:

Fusobacterium probe 1: GGUCGCAUAGCUCAGUUGGGAGAGCACC.

Fusobacterium probe 2:ACCGGGAUGGACAAACCUCUGAUGUACCAGUU.

miR-BART10-3p: TACATAACCATGGAGTTGGCT.

miR-BART19-5p. Cat. #4427975. Assay ID: 006693.

## Results

### Patient cohort

Patient characteristics are described in Additional file 1: Table S1. Of the 48 patients, 23 were men and 25 were women. Median age at diagnosis was 76 years (range 30–93). 18 patients were diagnosed with MSI and 30 with MSS. 17 patients were characterized with V600E-BRAF mutation, 9 with KRAS mutation and 16 with MLH1 methylation. 14 patients were diagnosed with left-sided tumor and 34 with right-sided tumor.

### MicroRNAs and isomiRs are consistently differentially expressed between tumors and normal colon across datasets

Based on a cohort of 48 stage I-II colon cancer patients, we set out to characterize consistent expression differences in sRNAs between cancer and normal tissue, and between colon cancer subtypes characterized by MSI status, BRAF and KRAS mutations, and tumor location (Additional file 1: Table S1). Small RNA sequencing of the 96 paired tumor and normal samples identified miRNAs as the dominant class of RNAs, followed by transfer RNAs (tRNAs), small nucleolar RNAs (snoRNAs), ribosomal RNAs (rRNAs), and other sRNAs (Additional file 2: Sequencing Statistics, Additional file 3: Figure S1A-C).

**Fig. 1:**
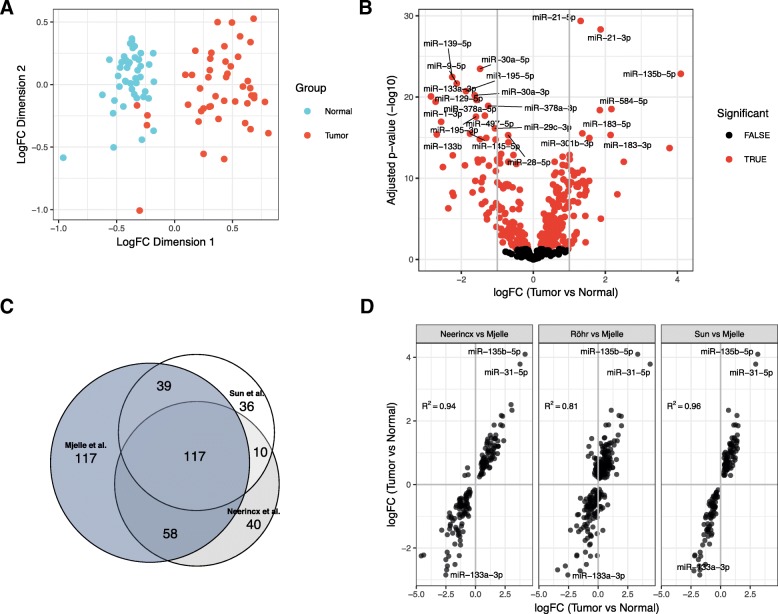
MicroRNAs and isomiRs are consistently differentially expressed between tumors and normal colon. **a** Multidimensional scaling plot (MDS plot) of the canonical miRNAs. Tumor samples are depicted in turquoise and matched adjacent normal samples are depicted in red. **b** Volcano plot showing expression differences for individual miRNAs between tumor and normal. The x-axis shows fold change values (log2) between tumor and normal tissue. The statistical comparison used was tumor vs normal such that a positive fold change indicates that the miRNA is upregulated in tumor tissue compared to normal tissue. The y-axis shows the minus log10 adjusted *p*-value, such that a higher value corresponds to higher significance. Red dots indicate that the miRNA is significant with adjusted p-value less than 0.05. **c** Venn-diagram showing the number and overlap of significant miRNAs in our dataset and the datasets of Neerincx and Sun. **d** Scatterplot comparing fold-change values between datasets. The most significantly upregulated miRNAs are shown with names as well as miR-133 which is the miRNA with the lowest logFC in our data. The statistical comparison is described in B). The correlation value is the Pearson correlation between the logFC values in the two datasets that are being compared

Focusing on the miRNA expression data revealed clear separation of most tumor samples from the adjacent normal mucosa (Fig. 1a). 331 miRNAs were detected as differentially expressed between tumor and normal, which includes about 66% of all miRNAs after filtering out those with low expression (Additional file 1: Table S2). Of these, 88 miRNAs had an absolute log fold-change (logFC) > 1 between tumor and normal (Fig. 1b). The miRNAs miR-135b-5p, miR-21-3p, miR-21-5p and miR-584-5p had higher expression in tumor tissue compared to normal tissue for all patients, making these miRNAs strong biomarker candidates for tumor tissue. Clustered miRNAs, such as the oncogenic miR-17-92 cluster, tended to have similar expression changes, suggesting that altered transcription explain many miRNA expression changes between tumor and normal (Additional file 4: Figure S2A, Additional file 3: Clustered miRNAs tend to be co-expressed).

For 48% of the detected miRNAs, the highest expressed miRNA sequence differed from the canonical miRNA sequence reported in miRBase, indicating that isomiRs are important to consider when analyzing miRNA expression (Additional file 4: Figure S2B). After filtering, we found 2451 of 3618 isomiRs (68%) to be differentially expressed. The significant isomiRs derived from 343 unique miRNAs, with hsa-miR-192-5p having the highest number of significant isomiRs (131 isomiRs).

To validate our differentially expressed miRNAs we reanalyzed three publicly available datasets that contained small RNA sequencing data for normal and tumor CRC tissue: Neerincx [26], Sun [27], and Röhr [7]. We detected 225 and 202 differentially expressed miRNAs for the Neerincx and Sun data sets, respectively, and 117 of these miRNAs shared significant differential expression with our data (Fig. 1c, Additional file 2: Table S2). The expression changes for the significant miRNAs were highly correlated between these three datasets, with only a few miRNAs being regulated in opposite direction (Fig. 1d). Although we detected no significant miRNAs in Röhr (Additional file 2: Table S2), expression changes were highly correlated between the miRNAs in Röhr and our data (Fig. 1d). Similarly, isomiR expression changes were highly correlated (Additional file 4: Figure S2C), confirming consistent miRNA expression differences between tumor and normal tissue for all four datasets.

### Several sRNA classes are consistently differentially expressed between tumors and normal colon across datasets

**Fig. 2:**
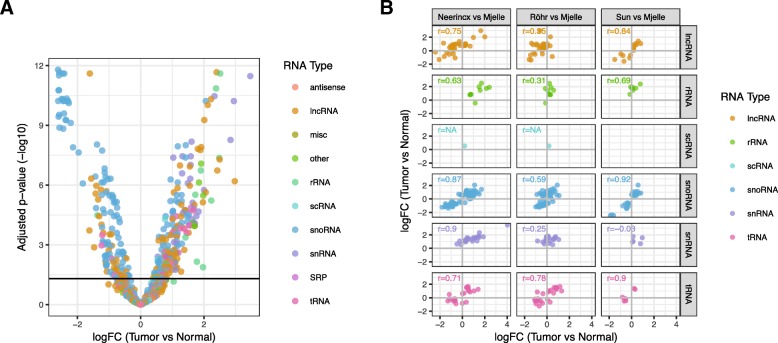
Several sRNAs classes are consistently differentially expressed. **a** Volcano plot showing expression differences for individual ncRNAs between tumor and normal (see Fig. 1b). Each RNA type is shown with a unique color. **b** Scatterplots comparing fold-change values between datasets for different ncRNA types (see Fig. 1d)

Having determined strong and consistent expression differences between tumor and normal tissue for miRNAs, we analyzed the other detected sRNAs. Of 18,735 unique non-miRNA sRNAs species, 363 were differentially expressed between tumor and normal tissue (Fig. 2a). Grouping the significant sRNAs by their class showed that small nuclear RNAs (snRNAs), rRNAs, signal recognition particle RNAs (SRPs), and other RNAs were upregulated in tumor cells (Additional file 5: Figure S3A). SnoRNAs was the only class downregulated in tumor cells, but this was largely due to several large snoRNA clusters being downregulated (Additional file 5: Figure S3B, C, Additional file 3: Clustered sRNAs). These clusters included two (ID12, ID13) located next to two miRNA clusters (ID2, ID3) and the *MEG3* and *MEG8* genes on chromosome 14 (Additional file 5: Figure S3D). Data from cap analysis of gene expression (CAGE) suggest that *MEG3* is the host gene for miRNA clusters ID2 and ID3 [29], and low expression of *MEG3* is previously shown to be associated with poor survival in CRC [30].

Comparing expression differences for sRNAs between ours and the Neerincx, Sun, and Röhr datasets showed general positive but varying correlations for the different sRNAs classes (Fig. 2b). SnoRNAs and tRNAs were highly correlated for all datasets, whereas snRNAs only showed strong correlation to our results in the Neerincx data. As for miRNAs, the Röhr dataset had no significant sRNAs, but showed positive correlations to our results. Overall, snoRNAs, tRNAs, lncRNAs, and rRNAs showed consistent differential expression between tumor and normal colon.

### MicroRNA differences between right and left normal colon are reduced in tumors

**Fig. 3:**
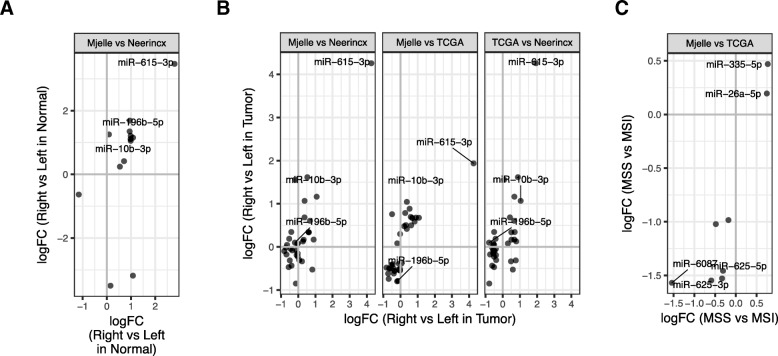
Meta-analysis of miRNAs associated to clinical parameters. **a**, **b** MicroRNAs differentially expressed between right and left (**a**) normal colon and (**b**) tumor tissue in the Neerincx, TCGA, and our datasets. Positive fold changes indicate that the corresponding miRNA is upregulated in right compared to left colon. **c** MicroRNAs differentially expressed between MSI positive and negative tumors in the TCGA and our datasets. Positive fold changes indicate that the corresponding miRNA is upregulated in MSS compared to MSI

Next we asked whether miRNA expression was associated with available clinicopathological characteristics (tumor location and MSI status) for samples in our cohort (Additional file 2: Table S1), in Neerincx, and for colon cancer samples in The Cancer Genome Atlas (TCGA) [31]. CRC tumors are classified as right or left depending on colon localization. The right-sided colon is derived from the midgut and includes the proximal two-thirds of the transverse colon, ascending colon and caecum, whereas left-sided colon is derived from the hindgut and includes the distal third of the transverse colon, splenic flexure, descending colon, sigmoid colon and rectum [32]. It is often challenging to define tissue samples from the transversum region as part of the right or left colon. We therefore investigated the differences in miRNAs expression between right and left colon for the normal samples with location 5 defined as right colon or having samples with location 5 removed from the analysis. By including location 5 to the right colon samples, we detected 10 differentially expressed miRNAs (Fig. 3a; Additional file 1: Table S3). By exlcuding samples with location 5 from the analysis, we detected the same 10 miRNAs as differentially expressed, in addtion to miR-1224-5p, which was downregulated in right colon. This shows that in our data there is only minor differences in miRNA expression depending on whether transversum is defined as right colon or remove from the analysis. We therefore chose to include samples with location 5 to the right colon samples. For the normal samples, we found most of the significant miRNAs to be upregulated in right colon (Fig. 3a; Additional file 1: Table S3). When comparing with the other datasets, the miRNAs showed consistent expression, except for miR-490, which was highly downregulated in Neerincx and slightly upregulated in our dataset. Three of the miRNAs upregulated in right normal colon – miR-615, miR-10b and miR-196b – reside in homeobox (Hox) clusters C, D, and A, respectively, indicating that some of the differences in miRNA expression could be due to differences in expression of Hox developmental genes (Additional file 5: Figure S3E). Differences in isomiR expression were also highly consistent between the two datasets (Additional file 6: Figure S4A). Next, we analyzed the tumor samples and compared right and left colon in our dataset and in Neerincx and TCGA, while adjusting for MSI status when available (not available in Neerincx). Overall, we observed consistent expression for most miRNAs and isomiRs across the datasets and especially high consistency between our dataset and TCGA (Fig. 3b; Additional file 6: Figure S4B; Additional file 1: Table S3).

Generally, different miRNAs were differentially expressed between right and left colon in cancer and normal tissue. MicroRNA miR-196b, for example, was highly upregulated in right normal tissue, but downregulated or unchanged in right vs left tumor tissue, indicating that this miRNA’s expression depended on location and disease. Overall, only miR-615-3p and its isomiRs showed significant and consistent expression differences between left and right colon in both normal and tumor tissue (Additional file 6: Figure S4C, D).

### Tumor mutation levels is associated with miR-155-5p expression

Moving on, we compared MSI positive and negative tumors in our dataset and TCGA, while adjusting for tumor location. Both miRNAs and isomiRs showed consistent expression differences in the two datasets (Fig. 3c; Additional file 6: Figure S4E, Additional file 1: Table S4), though only isomiRs for miR-26a-5p were significant in both datasets.

Microsatellite instability is associated with inactivation of DNA mismatch repair (MMR) genes causing increased mutation count in the cell. We therefore analyzed the association of loss-of-function or missense mutations in MMR genes and the number of somatic mutations in the TCGA dataset. Using linear modeling, we found that somatic loss-of-function or missense mutations in MMR genes were associated with approximately 8 and 4 times higher somatic mutation counts, respectively (estimated slopes of 3.02 and 2.01; *p* < 2e-16 and *p* = 2.85e-13; model r^2^ = 0.33).

As miR-155-5p, which was significantly downregulated in MSI tumors, has been shown to target the MMR genes in colon cancer [33], we asked whether adding the expression of miR-155-5p in the abovementioned model could improve its predictions of somatic mutation counts. Indeed, this combined model explained an additional 8% of the variation in mutation counts and estimated that doubling miR-155-5p expression gave a 1.5 increase in somatic mutation counts (slope 0.6; *p* = 3.25e-15; model r^2^ = 0.41). Increased mutation count was associated with increased miR-155-5p expression only in tumors negative for loss-of-function or missense mutations in MMR genes (Additional file 6: Figure S4F, G), which is consistent with miR-155-5p only affecting mutation levels when its MMR target genes produce functional proteins.

### Expression of bacteria and virus RNAs in CRC tissue

As both bacteria and viruses have been associated with CRC tissue, we hypothesized that the small RNA sequencing data could be used to detect expression of bacterial and viral small RNAs. We restricted the bacteria analysis to *F. nucleatum* only, due to its proposed role in CRC*,* whereas the virus analysis included all known viral miRNAs.

**Fig. 4:**
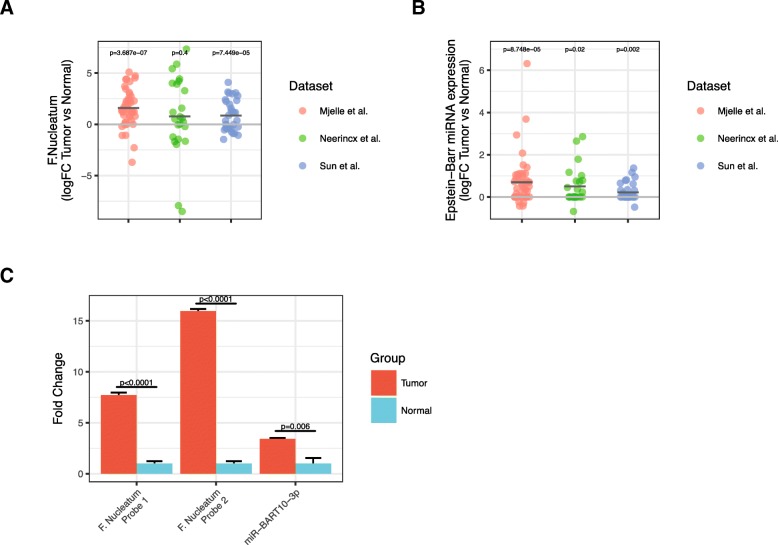
Expression of bacteria and virus RNAs in CRC tissue. **a**, **b** Fold-change values between paired tumor and normal tissue for (**a**) *F. nucleatum* and (**b**) Epstein-Barr virus in the Neerincx, Sun, and our datasets. **c** Bar-plot showing RT-qPCR validation of two probes against *F. nucleatum* and miR-BART10-5p. The normal samples are set to 1 and the error bars represent the standard deviation from three technical replicates. The experiment included 8 normal samples and 8 tumor samples

Paired tumor vs normal analyses of the Neerincx, Sun, and our datasets detected significant over-representation of *F. nucleatum* in tumors for two of the three datasets (Fig. 4a). In the TCGA dataset, which included 8 normal and 455 tumor samples, we detected *F. nucleatum* all normal and tumor samples and observed a trend towards increased expression in tumors (Additional file 7: Figure S5A, B).

Similar analyses on viruses detected miRNAs from Epstein-Barr virus (EBV) as highly upregulated in tumor samples compared to paired normal samples in all three datasets (Fig. 4b). In TCGA, 4 normal and 349 tumor samples had detectable levels of EBV miRNAs (Additional file 7: Figure S5C). Most of the detected EBV miRNAs were located in the BamHI A rightward transcripts (BARTs) cluster within the EBV genome; the BART miRNAs comprised 345 of 355 viral isomiRs. In total, we detected 28 different viral miRNAs, 25 of which were from EBV and 3 which were from and Human cytomegalovirus (HCMV). There was no correlation in tumor-expression of EBV miRNAs and *F. nucleatum* RNAs across the Neerincx, Sun, TCGA, and our datasets, indicating that presense of EBV and *F. nucleatum* in tumor tissue is independent (Additional file 7: Figure S5D).

**Fig. 5:**
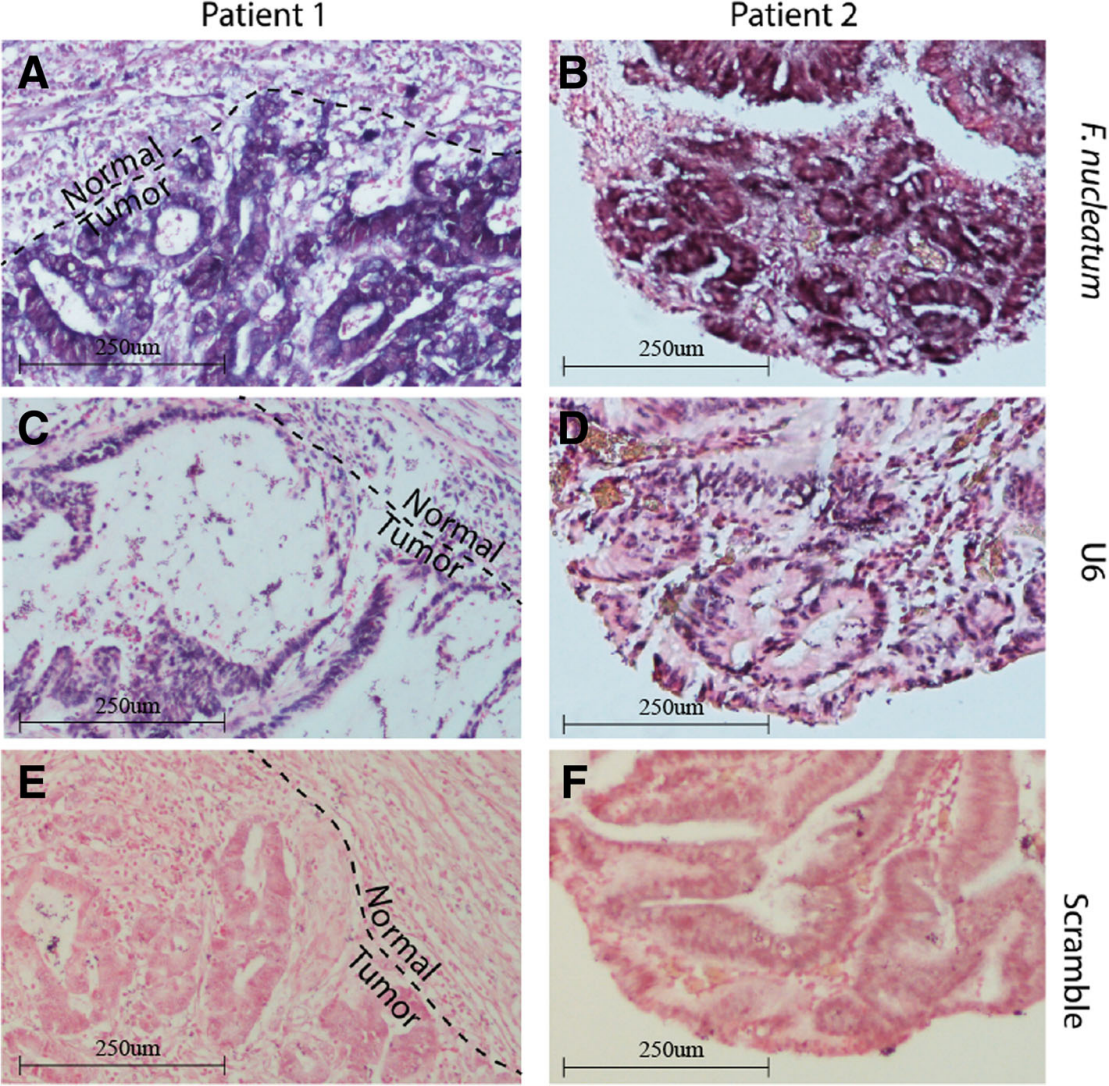
In situ hybridization (ISH) of CRC formalin-fixed paraffin-embedded (FFPE) tissue from two patients (left and right). ISH staining of a small RNA fragment from (**a**, **b**) F. nucleatum, (**c**, **d**) U6 positive control, and (**e**, **f**) scramble negative control. For patient 1, part of the section included non-tumor cells, which is indicated by the dashed line

We validated the expression differences by designing custom reverse transcription quantitative PCR (RT-qPCR) probes against two *F. nucleatum* RNAs and two EBV miRNAs, ebv-miR-BART10-3p and ebv-miR-BART19-5p and analyzing eight paired tumor and normal samples excluded from the sequencing experiment. The two *F. nucleatum* RNAs were highly upregulated in the tumor samples (Fig. 4c). MicroRNA miR-BART19-5p was undetected in 7 of the 8 normal samples and detected in all but one tumor sample (*p* = 0.04, McNemar’s test), whereas miR-BART10-3p levels were significantly increased in the tumor samples (Fig. 4c). Furthermore, in situ hybridization against the *F. nucleatum* RNA in two additional patient samples revealed strong *F. nucleatum* signal in cells with malignant morphology and weaker staining in adjacent normal cells (Fig. 5).

Finally, we asked whether EBV and *F. nucleatum* expression were associated with clinical parameters in the Neerincx, TCGA, and our datasets. We found no associations for MSI and BRAF and inconsistent patterns for KRAS mutation and *F. nucleatum*, but tumor location for *F. nucleatum* and KRAS mutation for EBV showed consistent trends for all datasets and were significant in one dataset each (Additional file 7: Figure S5E-L).

## Discussion

We have comprehensively analyzed miRNAs and sRNAs in early stage colon cancers and verified the major findings in our cohort by meta-analysis across multiple small RNA datasets.

MicroRNAs are known to be dysregulated in cancer and we find that much of this dysregulation is likely due to transcriptional effects on regions that contain several miRNAs in clusters. These include previously identified tumor suppressor (miR-154) and oncogenic (miR-17-92) loci [34, 35]. Similarly, we find several clustered snoRNAs, which are co-transcribed as part of host primary transcripts [36, 37], to be dysregulated. The growth arrest specific 5 (GAS5) long noncoding RNA hosts several snoRNAs [38], one of which (SNORD78, URS00006CFC33) is significantly upregulated in tumors in our study. GAS5 is implicated in a variety of cancers [39], and is shown to affect proliferation and to be a predictable prognostic marker in CRC patients [40]. In contrast to snoRNAs, the distinct upregulation of snRNAs in tumors cannot be explained by dysregulation of a common host gene, as these RNAs are located in different genomic regions. Interestingly, the tri-small ribonucleoprotein (tri-snRNP) spliceosome complex that binds snRNAs is commonly over-expressed in human primary and metastatic colon cancer [41]. This could explain why snRNAs are generally upregulated in tumor tissue.

We investigated if miRNA expression correlated with clinical parameters and if miRNAs could predict tumor subtypes. Consistent with a previous study [3], we found that miR-615-3p is highly upregulated in right-sided tumors compared to left-sided tumors. When examining the normal tissue, however, we also found miR-615-3p to be highly upregulated in right compared to left colon, suggesting that the expression difference between left- and right-sided tumors for miR-615-3p is due to underlying biological differences between left and right colon. Indeed, we found a larger set of miRNAs that separates left and right colon than separates left- and right-sided tumors. These include miR-196b and miR-10b, which are upregulated in right normal colon and, along with mir-615, are located within or in close proximity to the homeobox genes. The expression of these miRNAs is known to depend on these homeobox genes [42, 43]. The homeobox genes play a role in gut development [44], and because the right and left colon develop from the hindgut and midgut, respectively, differences in transcription patterns between the two regions could explain some of the region-related expression differences for these miRNAs. Notably, miR-196b, which was upregulated in right normal colon, was downregulated or unchanged in right tumor colon, indicating that additional mechanisms affect miR-196b expression in tumors.

Microsatellite instability is an important clinicopathological characteristic that determines treatment procedure and has prognostic value [45]. For instance, several recent studies have shown that MSI tumors respond well to immune therapy [46, 47] and have a nonbeneficial effect of adjuvant 5-FU-based chemotherapy [48]. We observed several miRNAs and isomiRs to be differentially expressed between MSI and MSS tumors – both in our and the TCGA cohort. Although few of these miRNAs were significant in both cohorts, the estimated expression differences for these miRNAs were highly reproducible (Fig. 3c). This high reproducibility suggests that the detected miRNAs do represent biological differences between MSI and MSS tumors. Indeed, whereas there was little overlap between significant MSI-related miRNAs in our and previous studies [2, 49–52], Earle et al., Lanza et al., Tang et al. and we detected miR-155 as upregulated in MSI tumors. Over-expression of miR-155 downregulates core DNA mismatch repair (MMR) genes and affects their protein expression [33]. We found that increased miR-155 levels correlate with increased mutation counts in tumors with intact MMR genes, suggesting a model where upregulation of miR-155 gives an increased mutational burden, possibly resulting in mutated MMR genes and MSI.

Bacteria have been associated with colorectal cancer, particularly the composition of the fecal and colon microbiome [53]. Sequencing of long RNAs and 16S DNA has detected *F. nucleatum* in tumor and normal colon tissue [11, 13]. Moreover, *F. nucleatum* is present in metastatic CRC tissue, indicating that the bacterium is transferred during metastasis. We show that small RNAs of *F. nucleatum* are present in colon tissue and that the expression is higher in tumor than in normal tissue. Bacterial small RNAs have been detected in other bacteria species and have a variety of regulatory mechanisms [54]. It remains to be investigated whether small RNAs from *F. nucleatum* possess regulatory properties that could affect tumor development. Several studies have detected viral DNA in colon cancer tumors, the most common types being Human papillomaviruses (HPV), Human polyomaviruses and Human herpesviruses [55]. Many of these viruses encode proteins that could potentially be oncogenic and affect proliferation of host cells [56]. The Human herpesvirus EBV contain viral miRNAs, most of which are encoded from the BART loci. Several of these miRNAs are shown to target host mRNAs and some EBV-miRNAs target genes involved in cancer development [57, 58]. Little is known about EBV-miRNAs in colon cancer, however, miR-BART19-3p is shown to target WIF1 [59], a gene important in colon cancer [60], and miR-BART1 is shown to target PSAT1 [61], a gene shown to promote proliferation of tumor cells [62]. However, none of these interactions have been shown in colon cancer cells. The EBV-miRNA miR-BART19-5p, which we found elevated in tumor tissue, is shown to target its own gene LMP1 [63], which is a functional homologue of tumor necrosis factor [64] that can downregulate host miR-146a. The EBV-miRNA miR-BART10-3p, which we found elevated in tumor tissue was previously found to directly targets *BTRC*, a gene found to be upregulated in colon cancer [65]. In summary, viral miRNAs could have significant impact on genes and cellular processes in the host and these interactions need further investigation, in particular in colon cancer.

## Conclusions

Small RNA profiling in colon cancer tissue revealed unique expression patterns in comparison with adjacent normal tissue. We detected several miRNAs that correlated with MSI and tumor location and show that miR-155 expression together with MMR deficiency explain much of the MSI-associated mutations in colon cancer. We show for the first time that small RNAs from *Fusobacterium nucleatum* are present in colon tissue, and that both *Fusobacterium nucleatum* and Epstein-Barr virus separate tumor and normal tissue.

## Additional files

Additional file 1: Table S1-S4. Differentially expression analysis and patient's characteristics. (DOCX 138 kb)

Additional file 2: Contains sequencing statistics results and additional methods description. (DOCX 49 kb)

Additional file 3: **Figure S1.** Sequencing statistics. **A)** Library size: Number of raw reads in the samples; Alignment: Number of reads that aligned to only one position in the genome (SingleAligned), multiple positions (MultiAligned) or not aligned (NotAligned); Features: Number of reads that aligned to miRBase, RNACentral database and to the calibrator RNAs; RNAs: Number of reads for the main RNA classes. **B)** Composition of RNAs in the samples shown as ratios. **C)** The sequences of the calibrator RNAs. (PDF 492 kb)

Additional file 4: **Figure S2.** MicroRNA clusters and isomiRs. **A)** A scatterplot of genomic clustered miRNAs showing log fold change values between tumor and normal (y-axis) for each individual miRNA within the clusters. The clusters are indicated with a unique ID on each facet of the plot. The figure only includes miRNAs that are significantly differentially expressed between tumor and normal. **B)** Illustrations of the main types of isomiRs analyzed in the current study, exemplified for hsa-miR-10b. **C)** Scatterplot comparing fold-change values for isomiRs between our dataset and the Neerincx, Röhr, and Sun datasets. (PDF 1751 kb)

Additional file 5: **Figure S3.** Classes of ncRNAs and snoRNA clusters. **A)** Boxplot showing expression of the sRNAs that differ significantly between tumor and normal samples, grouped by RNA classes. **B)** A scatterplot of genomic clustered snoRNAs showing log fold change values between tumor and normal (y-axis) for each individual snoRNA within the clusters. The clusters are indicated with a unique ID on each facet of the plot. The figure only includes snoRNAs that are significantly differentially expressed between tumor and normal. The *p*-values are calculated using a two-tailed student’s t-test. **C)** Log fold change values for snoRNAs that are not contained within a genomic cluster. **D)** Genome browser graphics of snoRNAs adjacent to the MEG3 gene. The graphics is from http://genome-euro.ucsc.edu. The miRNA clusters ID2 and ID3 are indicated with a red line below the graphics. **E)** Genome browser graphics of miR-10b, miR-615 and miR-196b and the adjacent RefSeq genes. (PDF 673 kb)

Additional file 6: **Figure S4.** MicroRNAs and isomiRs correlated with clinicopathological characteristics**. A)** Differentially expressed isomiRs in our dataset and the dataset of Neerincx et al. between right and left normal colon tissue. The comparison is Right-Left such that a positive fold change indicates that the corresponding miRNA is upregulated in right normal colon compared to left normal colon. **B)** Differentially expressed isomiRs in our data and the dataset of Neerincx et al. and TCGA comparing right and left tumor colon tissue. The statistical comparison is described in A). **C)** Differentially expressed miRNAs between right and left tumor tissue (x-axis) compared with differentially expressed miRNAs between right and left normal tissue (y-axis), for the datasets Mjelle et al. and Neerincx et al. **D)** Similar comparison as in C), for isomiRs. **E)** Differentially expressed isomiRs in our data and the dataset and TCGA comparing MSS and MSI. The comparison is MSS-MSI such that a positive fold change indicates that the corresponding isomiR is upregulated in MSS compared to MSI. **F-G)** Mutation counts for tumors grouped by miR-155 expression level and presence of somatic (F) loss-of-function or (G) missense mutations in MMR genes. The mean value is indicated with a cross. The *p*-values is the output from the linear model. (PDF 1150 kb)

Additional file 7: **Figure S5.** Expression of *F. nucleatum* and EBV miRNA *in* TCGA data and correlation with clinical parameters across data sets. The p-values in Fig. S5E-L are calculated using an unpaired two-tailed Student’s t-test. **A)** Expression of *F. nucleatum* in TCGA small RNA data. Left: All tumor samples compared to the eight normal samples. Right: The 8 paired tumor and normal samples. The p-values are calculated using an unpaired and paired two-tailed Student’s t-test for the left and right boxplot, respectively. **B)** Expression of *F. nucleatum* in TCGA colon samples. **C)** Expression of EBV miRNAs in TCGA colon samples. The figure shows the sum of all detected EBV miRNA for each sample as log2 and is normalized based on library sizes of mature miRNAs in the corresponding samples. **D)** Correlation between *F. nucleatum* and EBV miRNA in Neerincx et al., Sun et al., TCGA, and our datasets. The correlation is calculated using the cor() function in R with pearson correlation. **E)** *F. nucleatum* expression in MSI and MSS tumors in Mjelle et al. and TCGA. **F)** *F. nucleatum* expression in BRAF mutated and BRAF wild type (non-mutated) tumors in Mjelle et al. **G)** *F. nucleatum* expression in KRAS mutated and KRAS wild-type (non-mutated) tumors in Mjelle et al. and TCGA. **H)** *F. nucleatum* expression in right-and left-sided tumor tissue in Mjelle et al., Neerincx et al. and TCGA. **I)** EBV miRNA expression in right-and left-sided tumor tissue in Mjelle et al., Neerincx et al. and TCGA. **J)** EBV miRNA in BRAF mutated and BRAF wild type (non-mutated) tumors in Mjelle et al. **K)** EBV miRNA expression in MSI and MSS tumors in Mjelle et al. and TCGA. **L)** EBV miRNA expression in KRAS mutated and KRAS wild-type (non-mutated) tumors in Mjelle et al. and TCGA. (PDF 462 kb)

## Abbrevations

BARTs: BamHI A rightward transcripts
CRC: colorectal cancer
F. nucleatum: Fusobacterium nucleatum
Hox: Homeobox
HPV: Human Papilloma Virus
lncRNAs: long non-coding RNAs
logFC: log fold-change
miRNA: microRNA
MMR: DNA mismatch repair
MSI: microsatellite instability
PCR EBV: Epstein-Barr virus
RT-qPCR: Reverse transcription quantitative PCR
snoRNAs: small nucleolar RNAs
snRNAs: small nuclear RNA
sRNAs: small RNAs
SRPs: signal recognition particle RNAs
TCGA: The Cancer Genome Atlas
