# Supplementary material for: Small RNA expression from viruses, bacteria and human miRNAs in colon cancer tissue and its association with microsatellite instability and tumor location
Source: BMC Cancer. 2019 Feb 20;19:161. doi: 10.1186/s12885-019-5330-0 (PMC6381638; doi:10.1186/s12885-019-5330-0)
Supplement: Supplementary file 1 — Differentially expression analysis and patient's characteristics. (DOCX 138 kb) [file 12885_2019_5330_MOESM1_ESM.docx]

| **Table S1: Clinical and histopathological  characteristics of the investigated patient cohort.** | | |
| --- | --- | --- |
|  |  |  |
| **Gender** |  | **Number** |
|  | Female | 25 |
|  | Male | 23 |
| **Age at Diagnosis (years)** | |  |
|  | >90 | 4 |
|  | 80-90 | 17 |
|  | 70-80 | 14 |
|  | 60-70 | 5 |
|  | 50-60 | 6 |
|  | <34 | 2 |
| **MSI-Status** |  |  |
|  | MSI | 18 |
|  | MSS | 30 |
| **BRAF exon 15 (V600E)** | |  |
|  | Mutation | 17 |
|  | WT | 31 |
| **KRAS exon 2 and 3** | |  |
|  | Mutation | 9 |
|  | WT | 39 |
| **MLH1 Methylation** | |  |
|  | Methylation | 16 |
|  | WT | 32 |
| **Tumor Location*** | |  |
|  | 10=Rectum | 0 |
|  | 9= Rectosigmoid | 0 |
|  | 8=Sigmoideum | 14 |
|  | 7= Descendens | 0 |
|  | 6=Left flexur | 0 |
|  | 5=Transversum | 6 |
|  | 4=Right flexur | 5 |
|  | 3=Ascendens | 11 |
|  | 2=Coecum | 12 |
|  | 1=Appendix | 0 |
| MSI: Microsatellite instable tumour | |  |
| MSS: Microsatellite stable tumour | |  |
| * Location 1-5 is right sided cancer and location 6-10 is left sided cancer. | | |

| **Table S2:** Differentially expressed miRNA between tumor and normal tissue in our dataset, Sun et al. Röhr et al and Neerincx et al. The statistical comparison used was Tumor-Normal such that a positive Fold Change indicates that the corresponding miRNA is up-regulated in tumor compared to normal tissue. “miRNA” lists the canonical mature miRNA name (from miRBase 21.0); “Fold Change (Log2)” is the log2 fold change of the Tumor-Normal statistical comparison (corresponding to the log2 of the miRNAs's average expression in Tumor subtracting the average expression in Normal values computed by limma); “Average Expression” is the isomiRs average log2 cpm expression in the dataset (as computed by limma); “Adjusted P-value” is the Benjamini-Hochberg adjusted p-value. "Dataset" indicates the dataset analyzed. | | | | |
| --- | --- | --- | --- | --- |
| miRNA | Fold Change (Log2) | Average Expression | Adjusted P-value | Dataset |
| hsa-miR-21-5p | 1.313 | 15.411 | 4.282E-30 | Mjelle et al. |
| hsa-miR-21-3p | 1.868 | 10.847 | 4.774E-29 | Mjelle et al. |
| hsa-miR-30a-5p | -1.480 | 11.854 | 3.359E-24 | Mjelle et al. |
| hsa-miR-135b-5p | 4.096 | 3.763 | 1.383E-23 | Mjelle et al. |
| hsa-miR-139-5p | -2.255 | 4.014 | 3.371E-23 | Mjelle et al. |
| hsa-miR-9-5p | -2.129 | 4.764 | 2.149E-22 | Mjelle et al. |
| hsa-miR-195-5p | -1.873 | 8.886 | 1.883E-21 | Mjelle et al. |
| hsa-miR-30a-3p | -1.629 | 5.924 | 6.102E-21 | Mjelle et al. |
| hsa-miR-133a-3p | -2.846 | 6.534 | 8.438E-21 | Mjelle et al. |
| hsa-miR-378a-5p | -1.580 | 5.727 | 2.276E-20 | Mjelle et al. |
| hsa-miR-129-5p | -2.716 | 0.341 | 3.776E-20 | Mjelle et al. |
| hsa-miR-378a-3p | -1.255 | 12.078 | 1.230E-19 | Mjelle et al. |
| hsa-miR-584-5p | 2.169 | 4.113 | 3.025E-19 | Mjelle et al. |
| hsa-miR-183-5p | 1.847 | 9.024 | 4.087E-19 | Mjelle et al. |
| hsa-miR-497-5p | -1.348 | 7.481 | 1.957E-18 | Mjelle et al. |
| hsa-miR-195-3p | -1.595 | 3.095 | 2.623E-18 | Mjelle et al. |
| hsa-miR-1-3p | -2.563 | 3.295 | 1.116E-17 | Mjelle et al. |
| hsa-miR-29c-3p | -1.068 | 8.159 | 7.295E-17 | Mjelle et al. |
| hsa-miR-301b-3p | 1.361 | 5.215 | 3.009E-16 | Mjelle et al. |
| hsa-miR-145-5p | -1.761 | 10.008 | 3.212E-16 | Mjelle et al. |
| hsa-miR-133b | -2.685 | 1.442 | 4.025E-16 | Mjelle et al. |
| hsa-miR-183-3p | 2.146 | 1.295 | 4.740E-16 | Mjelle et al. |
| hsa-miR-28-5p | -0.701 | 8.312 | 4.977E-16 | Mjelle et al. |
| hsa-miR-10b-5p | -1.308 | 16.307 | 1.084E-15 | Mjelle et al. |
| hsa-miR-182-5p | 1.556 | 12.019 | 1.122E-15 | Mjelle et al. |
| hsa-miR-145-3p | -1.486 | 7.721 | 1.518E-15 | Mjelle et al. |
| hsa-miR-378c | -1.050 | 7.458 | 1.757E-15 | Mjelle et al. |
| hsa-miR-30e-3p | -0.694 | 7.792 | 3.742E-15 | Mjelle et al. |
| hsa-miR-31-5p | 3.785 | 4.352 | 1.936E-14 | Mjelle et al. |
| hsa-miR-125a-5p | -0.896 | 11.171 | 1.011E-13 | Mjelle et al. |
| hsa-miR-335-3p | 1.005 | 8.439 | 1.324E-13 | Mjelle et al. |
| hsa-miR-30e-5p | -0.554 | 12.387 | 1.391E-13 | Mjelle et al. |
| hsa-miR-218-1-3p | -2.239 | 1.455 | 1.525E-13 | Mjelle et al. |
| hsa-miR-92a-3p | 0.813 | 12.734 | 2.248E-13 | Mjelle et al. |
| hsa-miR-29c-5p | -0.945 | 3.767 | 3.430E-13 | Mjelle et al. |
| hsa-miR-188-5p | 1.012 | 2.769 | 4.753E-13 | Mjelle et al. |
| hsa-miR-378d | -1.010 | 4.948 | 5.011E-13 | Mjelle et al. |
| hsa-miR-218-5p | -1.548 | 4.749 | 5.101E-13 | Mjelle et al. |
| hsa-miR-338-3p | -1.411 | 6.577 | 5.246E-13 | Mjelle et al. |
| hsa-miR-26a-5p | -0.997 | 15.632 | 8.039E-13 | Mjelle et al. |
| hsa-miR-27a-3p | 0.591 | 11.005 | 9.497E-13 | Mjelle et al. |
| hsa-miR-7974 | 2.512 | 3.166 | 9.497E-13 | Mjelle et al. |
| hsa-miR-28-3p | -0.697 | 11.097 | 9.497E-13 | Mjelle et al. |
| hsa-miR-4662a-5p | -1.531 | 2.088 | 1.128E-12 | Mjelle et al. |
| hsa-miR-381-3p | -0.924 | 7.379 | 1.490E-12 | Mjelle et al. |
| hsa-miR-26b-5p | -0.472 | 11.904 | 1.779E-12 | Mjelle et al. |
| hsa-miR-17-3p | 0.814 | 4.649 | 2.267E-12 | Mjelle et al. |
| hsa-miR-504-5p | -1.902 | -0.124 | 2.594E-12 | Mjelle et al. |
| hsa-miR-30c-2-3p | -1.235 | 2.080 | 2.815E-12 | Mjelle et al. |
| hsa-miR-29a-5p | 0.924 | 2.709 | 2.815E-12 | Mjelle et al. |
| hsa-miR-204-5p | -2.514 | 4.215 | 4.191E-12 | Mjelle et al. |
| hsa-miR-96-5p | 1.371 | 5.267 | 9.813E-12 | Mjelle et al. |
| hsa-miR-17-5p | 1.059 | 8.674 | 1.146E-11 | Mjelle et al. |
| hsa-miR-10b-3p | -1.042 | 3.223 | 1.306E-11 | Mjelle et al. |
| hsa-miR-24-2-5p | 0.783 | 4.777 | 1.976E-11 | Mjelle et al. |
| hsa-miR-93-5p | 0.624 | 9.861 | 2.020E-11 | Mjelle et al. |
| hsa-miR-2467-5p | 1.137 | 2.676 | 2.034E-11 | Mjelle et al. |
| hsa-miR-92a-1-5p | 1.239 | 2.053 | 2.158E-11 | Mjelle et al. |
| hsa-miR-106b-5p | 0.580 | 7.310 | 4.087E-11 | Mjelle et al. |
| hsa-miR-424-3p | 1.129 | 2.627 | 4.689E-11 | Mjelle et al. |
| hsa-miR-138-5p | -1.399 | 3.419 | 8.250E-11 | Mjelle et al. |
| hsa-miR-221-5p | 0.836 | 5.453 | 1.410E-10 | Mjelle et al. |
| hsa-miR-19a-5p | 1.550 | 0.350 | 2.119E-10 | Mjelle et al. |
| hsa-miR-99a-5p | -1.614 | 6.096 | 2.119E-10 | Mjelle et al. |
| hsa-miR-224-5p | 1.375 | 5.957 | 2.119E-10 | Mjelle et al. |
| hsa-miR-181c-3p | 1.013 | 4.590 | 2.952E-10 | Mjelle et al. |
| hsa-let-7c-5p | -1.443 | 8.274 | 3.053E-10 | Mjelle et al. |
| hsa-miR-181d-5p | 0.942 | 5.613 | 3.084E-10 | Mjelle et al. |
| hsa-miR-328-3p | -1.001 | 2.256 | 3.233E-10 | Mjelle et al. |
| hsa-miR-450b-5p | 1.051 | 4.036 | 3.233E-10 | Mjelle et al. |
| hsa-miR-18a-3p | 1.300 | 2.197 | 3.756E-10 | Mjelle et al. |
| hsa-miR-25-5p | 1.259 | 1.818 | 4.761E-10 | Mjelle et al. |
| hsa-miR-23b-3p | -0.735 | 10.394 | 5.416E-10 | Mjelle et al. |
| hsa-miR-136-3p | -0.768 | 6.658 | 5.699E-10 | Mjelle et al. |
| hsa-miR-708-5p | 1.205 | 3.585 | 5.699E-10 | Mjelle et al. |
| hsa-miR-25-3p | 0.546 | 10.631 | 5.925E-10 | Mjelle et al. |
| hsa-miR-149-5p | -0.945 | 3.676 | 6.229E-10 | Mjelle et al. |
| hsa-miR-574-3p | -0.689 | 7.405 | 6.286E-10 | Mjelle et al. |
| hsa-miR-431-5p | 1.442 | 1.482 | 7.047E-10 | Mjelle et al. |
| hsa-miR-106a-5p | 0.989 | 4.802 | 7.748E-10 | Mjelle et al. |
| hsa-miR-130a-3p | -0.695 | 8.052 | 8.800E-10 | Mjelle et al. |
| hsa-miR-708-3p | 1.201 | 2.587 | 1.000E-09 | Mjelle et al. |
| hsa-miR-877-5p | 1.242 | 1.613 | 1.136E-09 | Mjelle et al. |
| hsa-miR-887-3p | -0.803 | 3.623 | 1.136E-09 | Mjelle et al. |
| hsa-miR-671-5p | 0.648 | 2.503 | 1.206E-09 | Mjelle et al. |
| hsa-miR-30c-5p | -0.577 | 10.110 | 1.336E-09 | Mjelle et al. |
| hsa-miR-136-5p | -0.839 | 5.192 | 1.712E-09 | Mjelle et al. |
| hsa-miR-19a-3p | 0.717 | 7.519 | 1.899E-09 | Mjelle et al. |
| hsa-miR-130b-3p | 0.639 | 5.833 | 2.000E-09 | Mjelle et al. |
| hsa-miR-186-5p | -0.377 | 11.942 | 2.596E-09 | Mjelle et al. |
| hsa-miR-147b | -1.437 | 5.437 | 2.609E-09 | Mjelle et al. |
| hsa-miR-7-5p | 1.464 | 4.944 | 3.000E-09 | Mjelle et al. |
| hsa-miR-29b-1-5p | 1.472 | 1.787 | 3.669E-09 | Mjelle et al. |
| hsa-miR-330-5p | 0.965 | 4.082 | 3.939E-09 | Mjelle et al. |
| hsa-miR-222-3p | 0.635 | 9.098 | 4.168E-09 | Mjelle et al. |
| hsa-miR-99a-3p | -1.689 | 1.329 | 5.628E-09 | Mjelle et al. |
| hsa-miR-215-5p | -2.247 | 12.445 | 6.330E-09 | Mjelle et al. |
| hsa-miR-301a-3p | 0.646 | 7.368 | 6.525E-09 | Mjelle et al. |
| hsa-miR-3176 | 1.433 | 1.341 | 7.738E-09 | Mjelle et al. |
| hsa-miR-345-5p | 0.691 | 6.500 | 7.830E-09 | Mjelle et al. |
| hsa-miR-7641 | 2.334 | 2.527 | 9.497E-09 | Mjelle et al. |
| hsa-miR-20a-3p | 0.857 | 1.607 | 1.005E-08 | Mjelle et al. |
| hsa-miR-223-3p | 1.290 | 8.217 | 1.120E-08 | Mjelle et al. |
| hsa-miR-3677-3p | 1.427 | 0.240 | 1.293E-08 | Mjelle et al. |
| hsa-miR-215-3p | -2.219 | 3.962 | 1.445E-08 | Mjelle et al. |
| hsa-miR-5701 | 0.861 | 1.612 | 1.508E-08 | Mjelle et al. |
| hsa-miR-92b-3p | 0.799 | 9.353 | 1.636E-08 | Mjelle et al. |
| hsa-miR-941 | 0.713 | 7.987 | 3.368E-08 | Mjelle et al. |
| hsa-miR-29a-3p | 0.430 | 10.600 | 4.039E-08 | Mjelle et al. |
| hsa-miR-493-5p | 0.839 | 3.642 | 4.439E-08 | Mjelle et al. |
| hsa-miR-18a-5p | 1.109 | 7.194 | 6.624E-08 | Mjelle et al. |
| hsa-miR-532-5p | 0.520 | 8.184 | 7.575E-08 | Mjelle et al. |
| hsa-miR-125b-2-3p | -1.337 | 4.246 | 7.660E-08 | Mjelle et al. |
| hsa-miR-101-3p | -0.408 | 10.685 | 8.188E-08 | Mjelle et al. |
| hsa-miR-20a-5p | 0.992 | 9.348 | 8.722E-08 | Mjelle et al. |
| hsa-miR-34a-3p | 1.240 | 0.504 | 9.759E-08 | Mjelle et al. |
| hsa-miR-2355-5p | 0.675 | 1.454 | 1.093E-07 | Mjelle et al. |
| hsa-miR-100-5p | -1.035 | 8.927 | 1.148E-07 | Mjelle et al. |
| hsa-miR-30d-5p | -0.363 | 12.504 | 1.314E-07 | Mjelle et al. |
| hsa-miR-4532 | -1.431 | 0.893 | 1.532E-07 | Mjelle et al. |
| hsa-miR-148b-5p | 0.554 | 5.627 | 1.847E-07 | Mjelle et al. |
| hsa-miR-34a-5p | 0.709 | 7.186 | 1.902E-07 | Mjelle et al. |
| hsa-miR-128-1-5p | 0.942 | 1.675 | 2.492E-07 | Mjelle et al. |
| hsa-miR-143-5p | -0.954 | 3.393 | 2.993E-07 | Mjelle et al. |
| hsa-miR-550a-3p | 0.703 | 2.337 | 3.199E-07 | Mjelle et al. |
| hsa-miR-589-3p | 0.978 | 1.491 | 4.087E-07 | Mjelle et al. |
| hsa-miR-190a-3p | -1.137 | 1.557 | 4.320E-07 | Mjelle et al. |
| hsa-miR-7706 | 0.600 | 4.205 | 4.624E-07 | Mjelle et al. |
| hsa-let-7e-3p | -0.875 | 1.374 | 4.757E-07 | Mjelle et al. |
| hsa-miR-490-3p | -2.364 | 0.106 | 5.065E-07 | Mjelle et al. |
| hsa-miR-450a-5p | 0.865 | 3.273 | 5.825E-07 | Mjelle et al. |
| hsa-miR-101-5p | -0.576 | 3.486 | 6.353E-07 | Mjelle et al. |
| hsa-let-7b-5p | -0.400 | 11.514 | 6.461E-07 | Mjelle et al. |
| hsa-miR-125b-5p | -0.941 | 8.392 | 7.861E-07 | Mjelle et al. |
| hsa-miR-421 | 0.584 | 5.518 | 1.063E-06 | Mjelle et al. |
| hsa-miR-642a-5p | -1.240 | 0.025 | 1.662E-06 | Mjelle et al. |
| hsa-miR-511-5p | -0.780 | 1.520 | 1.972E-06 | Mjelle et al. |
| hsa-miR-151a-3p | 0.291 | 10.818 | 2.323E-06 | Mjelle et al. |
| hsa-miR-337-3p | -0.792 | 2.154 | 2.547E-06 | Mjelle et al. |
| hsa-miR-375 | -1.302 | 12.194 | 3.113E-06 | Mjelle et al. |
| hsa-miR-501-3p | 0.478 | 5.495 | 3.921E-06 | Mjelle et al. |
| hsa-miR-769-5p | 0.408 | 7.579 | 3.970E-06 | Mjelle et al. |
| hsa-miR-592 | 1.445 | 3.840 | 4.366E-06 | Mjelle et al. |
| hsa-miR-27a-5p | 1.121 | 4.630 | 4.366E-06 | Mjelle et al. |
| hsa-miR-19b-3p | 0.535 | 9.531 | 5.501E-06 | Mjelle et al. |
| hsa-miR-493-3p | 0.869 | 1.915 | 5.724E-06 | Mjelle et al. |
| hsa-miR-338-5p | -0.945 | 2.764 | 7.521E-06 | Mjelle et al. |
| hsa-miR-550a-5p | 0.770 | 1.769 | 8.175E-06 | Mjelle et al. |
| hsa-miR-454-5p | 0.580 | 1.592 | 8.175E-06 | Mjelle et al. |
| hsa-miR-454-3p | 0.347 | 7.080 | 8.591E-06 | Mjelle et al. |
| hsa-miR-452-5p | 0.758 | 4.862 | 8.610E-06 | Mjelle et al. |
| hsa-miR-1301-3p | 0.499 | 2.532 | 8.779E-06 | Mjelle et al. |
| hsa-miR-455-5p | 0.542 | 5.322 | 9.651E-06 | Mjelle et al. |
| hsa-miR-3687 | 1.875 | 1.278 | 9.690E-06 | Mjelle et al. |
| hsa-miR-3150b-3p | -1.288 | -0.207 | 1.142E-05 | Mjelle et al. |
| hsa-miR-93-3p | 0.456 | 2.956 | 1.257E-05 | Mjelle et al. |
| hsa-miR-106b-3p | 0.450 | 6.625 | 1.344E-05 | Mjelle et al. |
| hsa-miR-582-3p | 0.595 | 4.965 | 1.408E-05 | Mjelle et al. |
| hsa-miR-128-3p | 0.342 | 6.213 | 1.557E-05 | Mjelle et al. |
| hsa-miR-331-3p | 0.440 | 5.100 | 1.867E-05 | Mjelle et al. |
| hsa-miR-501-5p | 0.723 | 2.167 | 2.006E-05 | Mjelle et al. |
| hsa-miR-1271-5p | -0.775 | 1.345 | 2.049E-05 | Mjelle et al. |
| hsa-miR-98-5p | 0.373 | 10.457 | 2.082E-05 | Mjelle et al. |
| hsa-miR-199b-5p | -0.576 | 7.975 | 2.438E-05 | Mjelle et al. |
| hsa-miR-15b-3p | 0.484 | 3.859 | 2.913E-05 | Mjelle et al. |
| hsa-miR-495-3p | -0.607 | 2.379 | 3.070E-05 | Mjelle et al. |
| hsa-miR-29b-3p | 0.497 | 7.606 | 3.113E-05 | Mjelle et al. |
| hsa-miR-487b-3p | -0.531 | 3.280 | 3.388E-05 | Mjelle et al. |
| hsa-miR-374b-3p | 0.465 | 3.423 | 3.403E-05 | Mjelle et al. |
| hsa-miR-3200-3p | 0.811 | 0.852 | 4.070E-05 | Mjelle et al. |
| hsa-miR-1468-5p | -0.665 | 2.997 | 4.612E-05 | Mjelle et al. |
| hsa-miR-181a-3p | 0.463 | 5.697 | 4.700E-05 | Mjelle et al. |
| hsa-miR-500b-5p | 0.533 | 2.677 | 6.006E-05 | Mjelle et al. |
| hsa-miR-500a-5p | 0.531 | 2.685 | 6.136E-05 | Mjelle et al. |
| hsa-miR-376c-3p | -0.587 | 2.618 | 6.780E-05 | Mjelle et al. |
| hsa-miR-1180-3p | 0.657 | 2.879 | 7.026E-05 | Mjelle et al. |
| hsa-miR-33a-5p | 0.557 | 3.543 | 7.300E-05 | Mjelle et al. |
| hsa-miR-221-3p | 0.365 | 9.742 | 7.300E-05 | Mjelle et al. |
| hsa-miR-7705 | 0.692 | 3.326 | 7.300E-05 | Mjelle et al. |
| hsa-miR-26b-3p | -0.364 | 4.131 | 7.341E-05 | Mjelle et al. |
| hsa-miR-625-5p | 0.652 | 3.465 | 7.984E-05 | Mjelle et al. |
| hsa-miR-625-3p | 0.684 | 3.021 | 8.317E-05 | Mjelle et al. |
| hsa-miR-203a-3p | 0.798 | 9.284 | 9.493E-05 | Mjelle et al. |
| hsa-miR-5571-3p | -1.027 | 1.064 | 9.640E-05 | Mjelle et al. |
| hsa-miR-374a-3p | 0.351 | 7.327 | 1.041E-04 | Mjelle et al. |
| hsa-let-7f-1-3p | 0.501 | 1.598 | 1.128E-04 | Mjelle et al. |
| hsa-miR-342-5p | -0.674 | 2.147 | 1.341E-04 | Mjelle et al. |
| hsa-miR-320c | 0.716 | 1.640 | 1.528E-04 | Mjelle et al. |
| hsa-miR-424-5p | 0.767 | 5.981 | 1.532E-04 | Mjelle et al. |
| hsa-miR-190a-5p | -0.896 | 7.648 | 1.666E-04 | Mjelle et al. |
| hsa-miR-411-5p | -0.539 | 7.756 | 1.701E-04 | Mjelle et al. |
| hsa-miR-148a-5p | 0.605 | 8.100 | 2.441E-04 | Mjelle et al. |
| hsa-miR-194-5p | -0.718 | 13.199 | 2.493E-04 | Mjelle et al. |
| hsa-miR-99b-5p | -0.392 | 10.053 | 2.622E-04 | Mjelle et al. |
| hsa-miR-542-3p | 0.592 | 3.183 | 2.805E-04 | Mjelle et al. |
| hsa-miR-192-5p | -0.681 | 17.672 | 2.878E-04 | Mjelle et al. |
| hsa-miR-1307-5p | 0.399 | 8.806 | 3.229E-04 | Mjelle et al. |
| hsa-miR-143-3p | -0.537 | 16.567 | 3.234E-04 | Mjelle et al. |
| hsa-miR-193a-3p | 0.494 | 4.593 | 3.303E-04 | Mjelle et al. |
| hsa-miR-342-3p | -0.485 | 8.035 | 4.168E-04 | Mjelle et al. |
| hsa-miR-331-5p | 0.439 | 1.826 | 4.183E-04 | Mjelle et al. |
| hsa-miR-3651 | 1.201 | 0.446 | 4.403E-04 | Mjelle et al. |
| hsa-miR-197-3p | -0.306 | 6.809 | 4.799E-04 | Mjelle et al. |
| hsa-miR-485-5p | -0.807 | 0.112 | 4.843E-04 | Mjelle et al. |
| hsa-miR-33b-5p | 0.525 | 4.610 | 5.090E-04 | Mjelle et al. |
| hsa-miR-1185-1-3p | 0.693 | 0.554 | 6.237E-04 | Mjelle et al. |
| hsa-miR-23a-3p | 0.239 | 9.794 | 6.485E-04 | Mjelle et al. |
| hsa-miR-335-5p | 0.346 | 6.617 | 6.559E-04 | Mjelle et al. |
| hsa-miR-29b-2-5p | -0.636 | 0.959 | 6.805E-04 | Mjelle et al. |
| hsa-miR-1247-5p | 1.196 | 3.314 | 6.893E-04 | Mjelle et al. |
| hsa-miR-199a-5p | -0.423 | 8.538 | 6.900E-04 | Mjelle et al. |
| hsa-miR-511-3p | -0.715 | 0.372 | 7.459E-04 | Mjelle et al. |
| hsa-miR-455-3p | 0.507 | 3.389 | 7.568E-04 | Mjelle et al. |
| hsa-miR-194-3p | -0.709 | 5.115 | 8.490E-04 | Mjelle et al. |
| hsa-miR-320a | -0.239 | 8.624 | 8.520E-04 | Mjelle et al. |
| hsa-miR-95-3p | 0.622 | 4.100 | 9.059E-04 | Mjelle et al. |
| hsa-miR-296-5p | -0.696 | 0.492 | 9.649E-04 | Mjelle et al. |
| hsa-miR-151b | -0.316 | 5.653 | 9.702E-04 | Mjelle et al. |
| hsa-miR-210-3p | 0.597 | 6.779 | 9.834E-04 | Mjelle et al. |
| hsa-miR-32-3p | 0.543 | 2.473 | 1.032E-03 | Mjelle et al. |
| hsa-miR-489-3p | -0.900 | 0.485 | 1.032E-03 | Mjelle et al. |
| hsa-miR-653-5p | -0.859 | 1.417 | 1.073E-03 | Mjelle et al. |
| hsa-miR-2277-5p | 0.679 | 0.263 | 1.095E-03 | Mjelle et al. |
| hsa-miR-320d | 0.695 | 1.120 | 1.100E-03 | Mjelle et al. |
| hsa-let-7g-5p | -0.294 | 12.200 | 1.109E-03 | Mjelle et al. |
| hsa-miR-3605-5p | 0.566 | 0.439 | 1.133E-03 | Mjelle et al. |
| hsa-miR-4449 | 1.141 | 0.982 | 1.133E-03 | Mjelle et al. |
| hsa-miR-339-5p | 0.347 | 4.833 | 1.178E-03 | Mjelle et al. |
| hsa-miR-103a-3p | 0.186 | 10.654 | 1.237E-03 | Mjelle et al. |
| hsa-miR-4636 | -0.750 | -0.008 | 1.240E-03 | Mjelle et al. |
| hsa-miR-212-3p | 0.405 | 3.646 | 1.402E-03 | Mjelle et al. |
| hsa-miR-33b-3p | 0.829 | 1.398 | 1.570E-03 | Mjelle et al. |
| hsa-miR-598-3p | -0.606 | 3.902 | 1.586E-03 | Mjelle et al. |
| hsa-miR-664a-3p | -0.379 | 4.648 | 1.722E-03 | Mjelle et al. |
| hsa-miR-652-5p | 0.505 | 2.202 | 1.751E-03 | Mjelle et al. |
| hsa-miR-192-3p | -0.669 | 5.010 | 1.815E-03 | Mjelle et al. |
| hsa-miR-1266-5p | 0.627 | 0.403 | 1.994E-03 | Mjelle et al. |
| hsa-miR-299-5p | -0.641 | 0.510 | 2.048E-03 | Mjelle et al. |
| hsa-miR-148a-3p | 0.457 | 14.622 | 2.201E-03 | Mjelle et al. |
| hsa-miR-181b-5p | 0.302 | 8.799 | 2.385E-03 | Mjelle et al. |
| hsa-miR-181c-5p | 0.336 | 9.229 | 2.414E-03 | Mjelle et al. |
| hsa-miR-590-3p | 0.252 | 4.566 | 2.673E-03 | Mjelle et al. |
| hsa-miR-363-3p | -0.663 | 5.179 | 2.720E-03 | Mjelle et al. |
| hsa-miR-425-5p | 0.329 | 8.929 | 2.730E-03 | Mjelle et al. |
| hsa-let-7i-5p | 0.304 | 11.112 | 2.839E-03 | Mjelle et al. |
| hsa-miR-3158-3p | 0.592 | 1.323 | 3.129E-03 | Mjelle et al. |
| hsa-miR-4677-3p | 0.335 | 3.102 | 3.138E-03 | Mjelle et al. |
| hsa-miR-212-5p | 0.413 | 1.612 | 3.321E-03 | Mjelle et al. |
| hsa-miR-577 | 0.567 | 7.804 | 3.334E-03 | Mjelle et al. |
| hsa-miR-30c-1-3p | -0.432 | 3.182 | 3.647E-03 | Mjelle et al. |
| hsa-miR-663b | 1.131 | 0.487 | 4.125E-03 | Mjelle et al. |
| hsa-miR-152-3p | -0.454 | 5.287 | 4.195E-03 | Mjelle et al. |
| hsa-miR-452-3p | 0.549 | 2.973 | 4.452E-03 | Mjelle et al. |
| hsa-miR-153-3p | -0.438 | 4.746 | 4.673E-03 | Mjelle et al. |
| hsa-miR-299-3p | -0.419 | 3.061 | 4.696E-03 | Mjelle et al. |
| hsa-miR-1307-3p | 0.341 | 6.012 | 5.106E-03 | Mjelle et al. |
| hsa-miR-654-3p | -0.385 | 6.307 | 5.127E-03 | Mjelle et al. |
| hsa-miR-203a-5p | 0.822 | 0.577 | 5.190E-03 | Mjelle et al. |
| hsa-miR-3615 | 0.380 | 2.989 | 5.208E-03 | Mjelle et al. |
| hsa-miR-185-5p | 0.281 | 3.860 | 5.726E-03 | Mjelle et al. |
| hsa-miR-140-3p | -0.239 | 8.463 | 5.743E-03 | Mjelle et al. |
| hsa-miR-628-3p | -0.338 | 1.799 | 5.759E-03 | Mjelle et al. |
| hsa-miR-1277-5p | 0.316 | 2.636 | 5.956E-03 | Mjelle et al. |
| hsa-miR-552-3p | 0.979 | 2.847 | 6.013E-03 | Mjelle et al. |
| hsa-miR-154-5p | -0.450 | 1.809 | 6.536E-03 | Mjelle et al. |
| hsa-miR-545-5p | 0.517 | 0.119 | 6.544E-03 | Mjelle et al. |
| hsa-miR-548o-3p | 0.388 | 3.312 | 6.706E-03 | Mjelle et al. |
| hsa-miR-130b-5p | 0.313 | 3.705 | 6.787E-03 | Mjelle et al. |
| hsa-miR-10a-3p | 0.434 | 5.418 | 7.482E-03 | Mjelle et al. |
| hsa-miR-615-3p | 1.451 | 0.916 | 7.756E-03 | Mjelle et al. |
| hsa-miR-320b | 0.310 | 4.427 | 8.006E-03 | Mjelle et al. |
| hsa-miR-374b-5p | -0.345 | 6.148 | 8.058E-03 | Mjelle et al. |
| hsa-miR-3653-3p | -0.851 | 2.882 | 8.058E-03 | Mjelle et al. |
| hsa-miR-425-3p | 0.226 | 4.208 | 8.294E-03 | Mjelle et al. |
| hsa-miR-4775 | 0.333 | 1.322 | 9.240E-03 | Mjelle et al. |
| hsa-miR-543 | -0.413 | 0.987 | 9.623E-03 | Mjelle et al. |
| hsa-miR-3609 | 0.537 | 3.568 | 9.826E-03 | Mjelle et al. |
| hsa-miR-451a | -0.616 | 10.886 | 1.026E-02 | Mjelle et al. |
| hsa-miR-589-5p | 0.257 | 4.765 | 1.047E-02 | Mjelle et al. |
| hsa-miR-3648 | 1.068 | 2.116 | 1.098E-02 | Mjelle et al. |
| hsa-miR-3909 | 0.333 | 2.148 | 1.130E-02 | Mjelle et al. |
| hsa-miR-485-3p | -0.459 | 1.852 | 1.134E-02 | Mjelle et al. |
| hsa-miR-146a-5p | 0.473 | 10.165 | 1.138E-02 | Mjelle et al. |
| hsa-miR-7704 | -0.444 | 2.336 | 1.247E-02 | Mjelle et al. |
| hsa-miR-193b-3p | -0.339 | 6.011 | 1.249E-02 | Mjelle et al. |
| hsa-miR-3912-3p | -0.285 | 1.814 | 1.336E-02 | Mjelle et al. |
| hsa-miR-1260b | 0.644 | 2.136 | 1.382E-02 | Mjelle et al. |
| hsa-miR-494-3p | 0.412 | 1.396 | 1.401E-02 | Mjelle et al. |
| hsa-miR-542-5p | 0.513 | 0.123 | 1.505E-02 | Mjelle et al. |
| hsa-miR-188-3p | 0.433 | 0.445 | 1.509E-02 | Mjelle et al. |
| hsa-miR-484 | -0.168 | 7.264 | 1.584E-02 | Mjelle et al. |
| hsa-miR-660-5p | 0.217 | 7.769 | 1.584E-02 | Mjelle et al. |
| hsa-miR-107 | 0.206 | 8.251 | 1.585E-02 | Mjelle et al. |
| hsa-miR-4772-5p | 0.517 | 0.549 | 1.667E-02 | Mjelle et al. |
| hsa-let-7b-3p | -0.225 | 3.770 | 1.733E-02 | Mjelle et al. |
| hsa-miR-95-5p | 0.519 | 0.635 | 1.745E-02 | Mjelle et al. |
| hsa-miR-5690 | -0.591 | 0.173 | 1.791E-02 | Mjelle et al. |
| hsa-miR-4746-5p | 0.559 | 0.444 | 1.817E-02 | Mjelle et al. |
| hsa-miR-582-5p | 0.306 | 4.616 | 1.822E-02 | Mjelle et al. |
| hsa-miR-502-5p | 0.316 | 1.607 | 1.865E-02 | Mjelle et al. |
| hsa-miR-377-3p | -0.335 | 2.292 | 1.871E-02 | Mjelle et al. |
| hsa-miR-6724-5p | 0.676 | 0.976 | 1.871E-02 | Mjelle et al. |
| hsa-miR-141-5p | 0.400 | 7.775 | 1.885E-02 | Mjelle et al. |
| hsa-miR-30d-3p | -0.239 | 5.708 | 2.018E-02 | Mjelle et al. |
| hsa-miR-223-5p | 0.460 | 2.812 | 2.025E-02 | Mjelle et al. |
| hsa-miR-150-5p | -0.449 | 8.314 | 2.123E-02 | Mjelle et al. |
| hsa-miR-641 | 0.322 | 2.006 | 2.290E-02 | Mjelle et al. |
| hsa-miR-1247-3p | 0.887 | 2.218 | 2.349E-02 | Mjelle et al. |
| hsa-miR-337-5p | -0.396 | 1.156 | 2.599E-02 | Mjelle et al. |
| hsa-miR-2110 | 0.308 | 1.267 | 2.599E-02 | Mjelle et al. |
| hsa-miR-379-5p | -0.367 | 1.720 | 2.845E-02 | Mjelle et al. |
| hsa-miR-126-5p | -0.191 | 12.409 | 2.862E-02 | Mjelle et al. |
| hsa-miR-200c-5p | 0.462 | 2.325 | 2.958E-02 | Mjelle et al. |
| hsa-miR-361-3p | -0.154 | 6.495 | 3.292E-02 | Mjelle et al. |
| hsa-miR-590-5p | 0.423 | 0.320 | 3.297E-02 | Mjelle et al. |
| hsa-miR-144-3p | -0.469 | 7.372 | 3.426E-02 | Mjelle et al. |
| hsa-miR-144-5p | -0.491 | 6.250 | 3.924E-02 | Mjelle et al. |
| hsa-miR-365a-3p | -0.256 | 4.854 | 3.945E-02 | Mjelle et al. |
| hsa-miR-365b-3p | -0.256 | 4.854 | 3.945E-02 | Mjelle et al. |
| hsa-miR-656-3p | -0.401 | 1.199 | 3.994E-02 | Mjelle et al. |
| hsa-miR-196b-5p | -0.432 | 8.480 | 4.244E-02 | Mjelle et al. |
| hsa-miR-369-5p | -0.324 | 1.888 | 4.290E-02 | Mjelle et al. |
| hsa-miR-664a-5p | -0.308 | 2.013 | 4.318E-02 | Mjelle et al. |
| hsa-miR-23a-5p | 0.784 | 2.623 | 4.318E-02 | Mjelle et al. |
| hsa-miR-500a-3p | 0.211 | 7.673 | 4.481E-02 | Mjelle et al. |
| hsa-miR-1296-5p | -0.248 | 4.294 | 4.617E-02 | Mjelle et al. |
| hsa-miR-142-3p | 0.284 | 8.375 | 4.659E-02 | Mjelle et al. |
| hsa-miR-4492 | 0.817 | 0.982 | 4.661E-02 | Mjelle et al. |
| hsa-miR-4485-3p | 0.970 | 2.602 | 4.808E-02 | Mjelle et al. |
| hsa-miR-27b-5p | 0.239 | 4.811 | 4.839E-02 | Mjelle et al. |
| hsa-miR-486-5p | -0.456 | 11.660 | 4.839E-02 | Mjelle et al. |
| hsa-miR-552-5p | 0.721 | 2.978 | 5.399E-02 | Mjelle et al. |
| hsa-miR-30b-5p | -0.193 | 10.069 | 5.454E-02 | Mjelle et al. |
| hsa-miR-191-5p | 0.203 | 14.424 | 5.601E-02 | Mjelle et al. |
| hsa-miR-127-3p | -0.237 | 10.390 | 5.606E-02 | Mjelle et al. |
| hsa-miR-382-5p | 0.252 | 2.306 | 5.696E-02 | Mjelle et al. |
| hsa-miR-548k | 0.192 | 4.351 | 5.909E-02 | Mjelle et al. |
| hsa-miR-671-3p | 0.238 | 4.252 | 6.054E-02 | Mjelle et al. |
| hsa-miR-126-3p | -0.320 | 10.078 | 6.342E-02 | Mjelle et al. |
| hsa-miR-4326 | 0.813 | 0.204 | 6.651E-02 | Mjelle et al. |
| hsa-miR-150-3p | -0.436 | 0.835 | 6.657E-02 | Mjelle et al. |
| hsa-let-7d-3p | -0.140 | 6.609 | 7.564E-02 | Mjelle et al. |
| hsa-miR-769-3p | 0.395 | 0.201 | 7.729E-02 | Mjelle et al. |
| hsa-miR-15b-5p | 0.156 | 7.631 | 7.729E-02 | Mjelle et al. |
| hsa-miR-185-3p | 0.268 | 0.604 | 7.961E-02 | Mjelle et al. |
| hsa-miR-203b-3p | 0.422 | 2.559 | 8.052E-02 | Mjelle et al. |
| hsa-miR-580-3p | 0.318 | 0.419 | 8.116E-02 | Mjelle et al. |
| hsa-miR-539-3p | -0.241 | 2.331 | 8.145E-02 | Mjelle et al. |
| hsa-miR-561-5p | -0.441 | 3.649 | 8.176E-02 | Mjelle et al. |
| hsa-miR-6087 | 0.937 | 6.977 | 8.296E-02 | Mjelle et al. |
| hsa-miR-340-5p | 0.103 | 8.309 | 8.624E-02 | Mjelle et al. |
| hsa-miR-627-3p | -0.295 | 0.606 | 8.665E-02 | Mjelle et al. |
| hsa-miR-20b-5p | -0.417 | 2.601 | 9.061E-02 | Mjelle et al. |
| hsa-miR-548e-5p | 0.184 | 2.043 | 9.128E-02 | Mjelle et al. |
| hsa-let-7g-3p | -0.231 | 1.619 | 9.299E-02 | Mjelle et al. |
| hsa-let-7i-3p | -0.223 | 4.370 | 1.004E-01 | Mjelle et al. |
| hsa-miR-196b-3p | -0.352 | 2.247 | 1.013E-01 | Mjelle et al. |
| hsa-miR-432-5p | -0.250 | 2.501 | 1.022E-01 | Mjelle et al. |
| hsa-miR-27b-3p | -0.160 | 14.514 | 1.069E-01 | Mjelle et al. |
| hsa-miR-155-5p | 0.261 | 8.420 | 1.112E-01 | Mjelle et al. |
| hsa-miR-1248 | -0.771 | 5.425 | 1.114E-01 | Mjelle et al. |
| hsa-let-7a-3p | 0.114 | 4.761 | 1.116E-01 | Mjelle et al. |
| hsa-miR-874-5p | 0.231 | 1.924 | 1.123E-01 | Mjelle et al. |
| hsa-miR-411-3p | -0.347 | 0.722 | 1.160E-01 | Mjelle et al. |
| hsa-miR-433-3p | -0.344 | 0.561 | 1.161E-01 | Mjelle et al. |
| hsa-miR-100-3p | -0.346 | 1.674 | 1.167E-01 | Mjelle et al. |
| hsa-miR-361-5p | -0.125 | 7.286 | 1.169E-01 | Mjelle et al. |
| hsa-miR-377-5p | -0.355 | 0.700 | 1.170E-01 | Mjelle et al. |
| hsa-miR-576-3p | 0.226 | 1.157 | 1.193E-01 | Mjelle et al. |
| hsa-miR-3934-5p | 0.341 | 0.577 | 1.207E-01 | Mjelle et al. |
| hsa-miR-4443 | 0.336 | 1.282 | 1.222E-01 | Mjelle et al. |
| hsa-miR-1246 | 0.881 | 2.526 | 1.237E-01 | Mjelle et al. |
| hsa-miR-431-3p | 0.334 | 0.763 | 1.292E-01 | Mjelle et al. |
| hsa-miR-3656 | 0.599 | 1.253 | 1.339E-01 | Mjelle et al. |
| hsa-miR-3613-5p | 0.207 | 4.368 | 1.361E-01 | Mjelle et al. |
| hsa-miR-487a-3p | 0.285 | 0.562 | 1.369E-01 | Mjelle et al. |
| hsa-miR-1303 | 0.582 | 0.283 | 1.408E-01 | Mjelle et al. |
| hsa-miR-196a-5p | -0.317 | 7.531 | 1.428E-01 | Mjelle et al. |
| hsa-miR-486-3p | -0.312 | 1.349 | 1.430E-01 | Mjelle et al. |
| hsa-let-7d-5p | 0.138 | 9.062 | 1.449E-01 | Mjelle et al. |
| hsa-miR-99b-3p | 0.212 | 2.276 | 1.479E-01 | Mjelle et al. |
| hsa-let-7a-5p | -0.147 | 14.555 | 1.488E-01 | Mjelle et al. |
| hsa-miR-548i | -0.674 | 1.008 | 1.488E-01 | Mjelle et al. |
| hsa-miR-30b-3p | -0.269 | 1.762 | 1.544E-01 | Mjelle et al. |
| hsa-miR-22-3p | -0.131 | 14.147 | 1.706E-01 | Mjelle et al. |
| hsa-miR-3116 | 0.233 | 0.601 | 1.760E-01 | Mjelle et al. |
| hsa-miR-410-3p | -0.171 | 5.592 | 1.809E-01 | Mjelle et al. |
| hsa-miR-132-5p | 0.170 | 2.139 | 1.819E-01 | Mjelle et al. |
| hsa-miR-629-5p | 0.151 | 3.187 | 1.974E-01 | Mjelle et al. |
| hsa-miR-10a-5p | 0.186 | 16.699 | 1.978E-01 | Mjelle et al. |
| hsa-miR-1287-5p | -0.217 | 1.277 | 2.016E-01 | Mjelle et al. |
| hsa-miR-4485-5p | 0.553 | 0.546 | 2.092E-01 | Mjelle et al. |
| hsa-miR-200a-5p | 0.210 | 6.923 | 2.139E-01 | Mjelle et al. |
| hsa-miR-3653-5p | -0.605 | 5.019 | 2.139E-01 | Mjelle et al. |
| hsa-miR-758-3p | 0.246 | 0.920 | 2.190E-01 | Mjelle et al. |
| hsa-miR-1275 | 0.364 | 2.619 | 2.193E-01 | Mjelle et al. |
| hsa-miR-665 | 0.247 | 0.914 | 2.194E-01 | Mjelle et al. |
| hsa-miR-652-3p | -0.104 | 4.886 | 2.197E-01 | Mjelle et al. |
| hsa-miR-214-3p | -0.190 | 6.011 | 2.208E-01 | Mjelle et al. |
| hsa-miR-210-5p | 0.297 | 1.878 | 2.238E-01 | Mjelle et al. |
| hsa-miR-532-3p | 0.121 | 3.865 | 2.249E-01 | Mjelle et al. |
| hsa-miR-24-1-5p | -0.163 | 2.006 | 2.350E-01 | Mjelle et al. |
| hsa-miR-142-5p | -0.167 | 12.476 | 2.372E-01 | Mjelle et al. |
| hsa-miR-1291 | 0.703 | 4.812 | 2.372E-01 | Mjelle et al. |
| hsa-miR-766-3p | 0.171 | 1.935 | 2.407E-01 | Mjelle et al. |
| hsa-miR-24-3p | 0.098 | 8.682 | 2.433E-01 | Mjelle et al. |
| hsa-miR-146b-5p | -0.151 | 11.868 | 2.474E-01 | Mjelle et al. |
| hsa-miR-942-5p | 0.217 | 0.986 | 2.612E-01 | Mjelle et al. |
| hsa-let-7e-5p | -0.150 | 9.107 | 2.728E-01 | Mjelle et al. |
| hsa-miR-628-5p | -0.152 | 2.136 | 2.735E-01 | Mjelle et al. |
| hsa-miR-744-5p | 0.108 | 5.274 | 2.752E-01 | Mjelle et al. |
| hsa-miR-362-3p | 0.170 | 1.421 | 2.768E-01 | Mjelle et al. |
| hsa-miR-3065-3p | -0.272 | 0.290 | 2.847E-01 | Mjelle et al. |
| hsa-miR-146b-3p | -0.154 | 5.237 | 2.866E-01 | Mjelle et al. |
| hsa-miR-181a-2-3p | 0.117 | 6.393 | 3.042E-01 | Mjelle et al. |
| hsa-miR-873-5p | -0.252 | 0.233 | 3.286E-01 | Mjelle et al. |
| hsa-miR-423-3p | -0.075 | 8.535 | 3.300E-01 | Mjelle et al. |
| hsa-miR-744-3p | -0.130 | 1.510 | 3.326E-01 | Mjelle et al. |
| hsa-miR-4516 | 0.482 | 2.372 | 3.433E-01 | Mjelle et al. |
| hsa-miR-323a-3p | -0.170 | 1.230 | 3.536E-01 | Mjelle et al. |
| hsa-miR-140-5p | -0.095 | 4.557 | 3.568E-01 | Mjelle et al. |
| hsa-miR-200b-3p | 0.152 | 11.537 | 3.597E-01 | Mjelle et al. |
| hsa-miR-200b-5p | -0.159 | 5.585 | 3.743E-01 | Mjelle et al. |
| hsa-miR-224-3p | -0.197 | 1.194 | 3.772E-01 | Mjelle et al. |
| hsa-miR-362-5p | 0.163 | 5.479 | 3.876E-01 | Mjelle et al. |
| hsa-miR-16-5p | -0.065 | 12.661 | 3.880E-01 | Mjelle et al. |
| hsa-miR-423-5p | 0.098 | 7.744 | 3.920E-01 | Mjelle et al. |
| hsa-miR-3131 | -0.284 | -0.170 | 3.999E-01 | Mjelle et al. |
| hsa-miR-3065-5p | 0.223 | 0.546 | 4.022E-01 | Mjelle et al. |
| hsa-miR-382-3p | 0.191 | 1.131 | 4.032E-01 | Mjelle et al. |
| hsa-miR-125a-3p | 0.134 | 1.719 | 4.112E-01 | Mjelle et al. |
| hsa-miR-574-5p | -0.449 | 3.206 | 4.143E-01 | Mjelle et al. |
| hsa-miR-409-3p | 0.101 | 6.388 | 4.143E-01 | Mjelle et al. |
| hsa-miR-429 | 0.141 | 10.192 | 4.195E-01 | Mjelle et al. |
| hsa-miR-1306-5p | -0.136 | 0.418 | 4.239E-01 | Mjelle et al. |
| hsa-miR-33a-3p | 0.117 | 1.877 | 4.303E-01 | Mjelle et al. |
| hsa-miR-324-5p | -0.088 | 5.033 | 4.384E-01 | Mjelle et al. |
| hsa-miR-34b-5p | 0.162 | 0.255 | 4.384E-01 | Mjelle et al. |
| hsa-miR-340-3p | 0.114 | 2.701 | 4.444E-01 | Mjelle et al. |
| hsa-miR-324-3p | 0.097 | 2.283 | 4.465E-01 | Mjelle et al. |
| hsa-miR-374a-5p | -0.073 | 7.199 | 4.530E-01 | Mjelle et al. |
| hsa-miR-370-3p | -0.129 | 1.223 | 4.930E-01 | Mjelle et al. |
| hsa-miR-874-3p | 0.102 | 4.465 | 5.044E-01 | Mjelle et al. |
| hsa-miR-3607-3p | -0.331 | 9.520 | 5.318E-01 | Mjelle et al. |
| hsa-miR-409-5p | 0.119 | 0.531 | 5.360E-01 | Mjelle et al. |
| hsa-miR-181a-5p | -0.060 | 13.411 | 5.365E-01 | Mjelle et al. |
| hsa-miR-127-5p | -0.094 | 2.587 | 5.381E-01 | Mjelle et al. |
| hsa-miR-132-3p | -0.076 | 6.690 | 5.493E-01 | Mjelle et al. |
| hsa-miR-191-3p | 0.130 | 1.619 | 5.581E-01 | Mjelle et al. |
| hsa-miR-1249-3p | 0.116 | 0.972 | 5.612E-01 | Mjelle et al. |
| hsa-miR-548e-3p | 0.069 | 2.078 | 5.656E-01 | Mjelle et al. |
| hsa-miR-339-3p | 0.053 | 5.611 | 6.041E-01 | Mjelle et al. |
| hsa-miR-3664-3p | 0.109 | 0.073 | 6.078E-01 | Mjelle et al. |
| hsa-miR-26a-1-3p | 0.123 | 0.846 | 6.136E-01 | Mjelle et al. |
| hsa-miR-219a-5p | 0.125 | 0.105 | 6.136E-01 | Mjelle et al. |
| hsa-miR-15a-5p | -0.037 | 8.406 | 6.164E-01 | Mjelle et al. |
| hsa-miR-200c-3p | -0.087 | 11.153 | 6.199E-01 | Mjelle et al. |
| hsa-miR-26a-2-3p | 0.065 | 3.524 | 6.199E-01 | Mjelle et al. |
| hsa-miR-193a-5p | -0.093 | 4.730 | 6.272E-01 | Mjelle et al. |
| hsa-miR-152-5p | 0.082 | 1.516 | 6.273E-01 | Mjelle et al. |
| hsa-miR-889-3p | -0.067 | 4.220 | 6.284E-01 | Mjelle et al. |
| hsa-miR-6125 | -0.116 | 0.205 | 6.285E-01 | Mjelle et al. |
| hsa-miR-323b-3p | 0.105 | 0.538 | 6.316E-01 | Mjelle et al. |
| hsa-miR-18b-5p | 0.114 | 1.056 | 6.344E-01 | Mjelle et al. |
| hsa-let-7f-2-3p | 0.102 | 0.254 | 6.360E-01 | Mjelle et al. |
| hsa-miR-199a-3p | -0.057 | 11.070 | 6.408E-01 | Mjelle et al. |
| hsa-miR-199b-3p | -0.057 | 11.069 | 6.423E-01 | Mjelle et al. |
| hsa-miR-1285-3p | 0.138 | 2.170 | 6.464E-01 | Mjelle et al. |
| hsa-miR-376b-3p | 0.094 | 0.460 | 6.621E-01 | Mjelle et al. |
| hsa-miR-655-3p | 0.090 | 0.303 | 6.849E-01 | Mjelle et al. |
| hsa-miR-34c-5p | 0.062 | 3.137 | 6.984E-01 | Mjelle et al. |
| hsa-miR-3913-5p | 0.060 | 0.756 | 6.994E-01 | Mjelle et al. |
| hsa-miR-502-3p | 0.030 | 4.414 | 7.057E-01 | Mjelle et al. |
| hsa-miR-3614-5p | -0.081 | 1.608 | 7.215E-01 | Mjelle et al. |
| hsa-miR-576-5p | -0.033 | 3.463 | 7.255E-01 | Mjelle et al. |
| hsa-miR-659-5p | 0.065 | 0.731 | 7.255E-01 | Mjelle et al. |
| hsa-miR-627-5p | 0.047 | 1.023 | 7.547E-01 | Mjelle et al. |
| hsa-miR-499a-5p | 0.079 | 1.296 | 7.646E-01 | Mjelle et al. |
| hsa-miR-1185-5p | -0.054 | 1.587 | 7.690E-01 | Mjelle et al. |
| hsa-miR-3605-3p | 0.058 | 0.313 | 7.894E-01 | Mjelle et al. |
| hsa-miR-134-5p | -0.046 | 2.914 | 7.918E-01 | Mjelle et al. |
| hsa-miR-330-3p | -0.042 | 1.630 | 7.928E-01 | Mjelle et al. |
| hsa-miR-664b-3p | 0.049 | 0.726 | 8.042E-01 | Mjelle et al. |
| hsa-miR-369-3p | 0.028 | 3.233 | 8.231E-01 | Mjelle et al. |
| hsa-miR-200a-3p | 0.038 | 10.293 | 8.339E-01 | Mjelle et al. |
| hsa-miR-125b-1-3p | 0.039 | 3.429 | 8.514E-01 | Mjelle et al. |
| hsa-miR-151a-5p | -0.018 | 10.763 | 8.514E-01 | Mjelle et al. |
| hsa-miR-651-5p | -0.025 | 2.706 | 8.651E-01 | Mjelle et al. |
| hsa-miR-22-5p | 0.015 | 4.302 | 8.807E-01 | Mjelle et al. |
| hsa-miR-376a-5p | -0.022 | 1.972 | 8.857E-01 | Mjelle et al. |
| hsa-miR-141-3p | 0.026 | 13.425 | 8.904E-01 | Mjelle et al. |
| hsa-miR-616-5p | -0.024 | 0.938 | 9.001E-01 | Mjelle et al. |
| hsa-miR-505-3p | -0.010 | 4.580 | 9.025E-01 | Mjelle et al. |
| hsa-miR-556-5p | 0.027 | 0.190 | 9.108E-01 | Mjelle et al. |
| hsa-miR-3613-3p | -0.017 | 1.201 | 9.303E-01 | Mjelle et al. |
| hsa-miR-7-1-3p | 0.006 | 3.998 | 9.652E-01 | Mjelle et al. |
| hsa-miR-32-5p | 0.002 | 5.620 | 9.821E-01 | Mjelle et al. |
| hsa-miR-148b-3p | 0.001 | 8.646 | 9.970E-01 | Mjelle et al. |
| hsa-let-7f-5p | 0.000 | 13.612 | 9.987E-01 | Mjelle et al. |
| hsa-miR-214-5p | -0.001 | 4.193 | 9.987E-01 | Mjelle et al. |
| hsa-miR-16-2-3p | 0.000 | 5.304 | 9.990E-01 | Mjelle et al. |
| hsa-miR-133b | -3.418 | 0.445 | 8.181E-02 | Röhr et al. |
| hsa-miR-31-5p | 4.392 | 6.057 | 9.283E-02 | Röhr et al. |
| hsa-miR-137 | -2.909 | -0.460 | 9.283E-02 | Röhr et al. |
| hsa-miR-1224-5p | -3.050 | 1.744 | 1.432E-01 | Röhr et al. |
| hsa-miR-1-3p | -2.762 | 13.315 | 1.860E-01 | Röhr et al. |
| hsa-miR-133a-3p | -2.556 | 3.667 | 2.486E-01 | Röhr et al. |
| hsa-miR-124-3p | -2.058 | 1.553 | 2.864E-01 | Röhr et al. |
| hsa-miR-338-3p | -1.458 | 3.775 | 2.864E-01 | Röhr et al. |
| hsa-miR-584-5p | 1.766 | 4.067 | 2.864E-01 | Röhr et al. |
| hsa-miR-135b-5p | 3.344 | 3.948 | 2.864E-01 | Röhr et al. |
| hsa-miR-183-5p | 1.975 | 4.208 | 3.101E-01 | Röhr et al. |
| hsa-miR-3117-3p | 1.572 | 1.291 | 3.101E-01 | Röhr et al. |
| hsa-miR-129-2-3p | -2.235 | 3.300 | 3.101E-01 | Röhr et al. |
| hsa-miR-663a | 1.381 | -0.016 | 3.101E-01 | Röhr et al. |
| hsa-miR-145-3p | -1.647 | 6.487 | 3.101E-01 | Röhr et al. |
| hsa-miR-21-5p | 1.107 | 16.313 | 3.101E-01 | Röhr et al. |
| hsa-miR-147b | -2.332 | 4.150 | 3.101E-01 | Röhr et al. |
| hsa-miR-29a-3p | 0.841 | 13.948 | 3.101E-01 | Röhr et al. |
| hsa-miR-873-5p | -1.414 | 0.452 | 3.101E-01 | Röhr et al. |
| hsa-miR-143-5p | -1.742 | 8.160 | 3.274E-01 | Röhr et al. |
| hsa-miR-17-3p | 1.016 | 7.591 | 3.288E-01 | Röhr et al. |
| hsa-miR-96-5p | 1.615 | 4.301 | 3.420E-01 | Röhr et al. |
| hsa-miR-143-3p | -1.129 | 12.825 | 3.420E-01 | Röhr et al. |
| hsa-miR-92a-1-5p | 1.350 | 4.717 | 3.420E-01 | Röhr et al. |
| hsa-miR-375 | -1.810 | 10.978 | 3.420E-01 | Röhr et al. |
| hsa-miR-224-5p | 1.375 | 7.241 | 3.420E-01 | Röhr et al. |
| hsa-miR-182-5p | 1.695 | 5.339 | 3.476E-01 | Röhr et al. |
| hsa-miR-338-5p | -1.651 | 1.358 | 3.476E-01 | Röhr et al. |
| hsa-miR-183-3p | 1.957 | 1.449 | 3.476E-01 | Röhr et al. |
| hsa-miR-7641 | 1.112 | 4.576 | 3.476E-01 | Röhr et al. |
| hsa-miR-23a-5p | 1.547 | 1.172 | 3.476E-01 | Röhr et al. |
| hsa-miR-642a-3p | -1.252 | 1.682 | 3.476E-01 | Röhr et al. |
| hsa-miR-129-1-3p | -2.464 | 1.495 | 3.476E-01 | Röhr et al. |
| hsa-miR-1304-5p | 0.986 | 1.092 | 3.476E-01 | Röhr et al. |
| hsa-miR-551b-3p | -1.623 | 0.738 | 3.476E-01 | Röhr et al. |
| hsa-miR-503-5p | 1.551 | 4.589 | 3.476E-01 | Röhr et al. |
| hsa-miR-6087 | 1.097 | 6.712 | 3.476E-01 | Röhr et al. |
| hsa-miR-139-3p | -1.342 | 2.129 | 3.476E-01 | Röhr et al. |
| hsa-miR-122-5p | 2.666 | 5.949 | 3.476E-01 | Röhr et al. |
| hsa-miR-145-5p | -1.095 | 12.318 | 3.476E-01 | Röhr et al. |
| hsa-miR-455-3p | 0.981 | 5.930 | 3.966E-01 | Röhr et al. |
| hsa-miR-378a-3p | -1.119 | 13.138 | 3.966E-01 | Röhr et al. |
| hsa-miR-133a-5p | -1.847 | 1.388 | 3.966E-01 | Röhr et al. |
| hsa-miR-497-5p | -1.239 | 7.919 | 3.966E-01 | Röhr et al. |
| hsa-miR-378b | -1.398 | 0.675 | 4.022E-01 | Röhr et al. |
| hsa-miR-3687 | 0.888 | 3.107 | 4.151E-01 | Röhr et al. |
| hsa-miR-129-5p | -1.519 | 0.425 | 4.151E-01 | Röhr et al. |
| hsa-miR-502-5p | 1.563 | 2.553 | 4.151E-01 | Röhr et al. |
| hsa-miR-452-5p | 0.885 | 8.340 | 4.151E-01 | Röhr et al. |
| hsa-miR-485-5p | -1.148 | 4.370 | 4.302E-01 | Röhr et al. |
| hsa-let-7a-5p | 0.646 | 16.293 | 4.736E-01 | Röhr et al. |
| hsa-miR-1185-2-3p | 0.985 | 0.845 | 4.736E-01 | Röhr et al. |
| hsa-miR-548ai | -1.043 | 0.223 | 4.736E-01 | Röhr et al. |
| hsa-miR-378c | -0.983 | 9.677 | 4.736E-01 | Röhr et al. |
| hsa-miR-192-5p | -0.882 | 17.400 | 4.736E-01 | Röhr et al. |
| hsa-miR-570-5p | -1.037 | 0.238 | 4.736E-01 | Röhr et al. |
| hsa-miR-1292-5p | 1.085 | 0.146 | 4.789E-01 | Röhr et al. |
| hsa-miR-421 | 0.887 | 5.086 | 4.789E-01 | Röhr et al. |
| hsa-miR-20a-3p | 0.857 | 2.139 | 5.105E-01 | Röhr et al. |
| hsa-miR-30a-5p | -0.882 | 9.884 | 5.105E-01 | Röhr et al. |
| hsa-miR-10b-3p | -1.194 | 2.152 | 5.105E-01 | Röhr et al. |
| hsa-miR-1254 | 0.972 | 0.731 | 5.105E-01 | Röhr et al. |
| hsa-miR-320d | 1.149 | 4.650 | 5.125E-01 | Röhr et al. |
| hsa-miR-1260b | 0.849 | 2.951 | 5.125E-01 | Röhr et al. |
| hsa-let-7d-5p | 0.739 | 12.640 | 5.125E-01 | Röhr et al. |
| hsa-miR-215-5p | -1.801 | 12.459 | 5.125E-01 | Röhr et al. |
| hsa-miR-219a-5p | 0.979 | 0.907 | 5.125E-01 | Röhr et al. |
| hsa-miR-665 | 0.955 | 0.446 | 5.125E-01 | Röhr et al. |
| hsa-miR-27b-5p | 0.741 | 6.695 | 5.125E-01 | Röhr et al. |
| hsa-miR-194-3p | -0.886 | 1.652 | 5.125E-01 | Röhr et al. |
| hsa-miR-378g | -1.148 | 1.233 | 5.125E-01 | Röhr et al. |
| hsa-miR-1185-1-3p | 0.770 | 2.616 | 5.125E-01 | Röhr et al. |
| hsa-miR-130b-5p | 0.813 | 2.124 | 5.125E-01 | Röhr et al. |
| hsa-miR-491-5p | 0.926 | 1.347 | 5.125E-01 | Röhr et al. |
| hsa-miR-511-3p | -0.824 | 5.087 | 5.125E-01 | Röhr et al. |
| hsa-miR-196b-5p | -1.493 | 8.714 | 5.125E-01 | Röhr et al. |
| hsa-miR-18a-3p | 0.964 | 1.587 | 5.125E-01 | Röhr et al. |
| hsa-miR-20a-5p | 1.174 | 7.123 | 5.125E-01 | Röhr et al. |
| hsa-miR-215-3p | -1.788 | 3.856 | 5.125E-01 | Röhr et al. |
| hsa-miR-23c | 1.166 | 2.808 | 5.125E-01 | Röhr et al. |
| hsa-miR-450b-5p | 0.839 | 1.180 | 5.125E-01 | Röhr et al. |
| hsa-miR-1266-5p | 0.977 | 2.047 | 5.125E-01 | Röhr et al. |
| hsa-miR-337-3p | -0.987 | 0.672 | 5.125E-01 | Röhr et al. |
| hsa-miR-92b-5p | 1.047 | 4.063 | 5.125E-01 | Röhr et al. |
| hsa-miR-27b-3p | 0.728 | 10.180 | 5.125E-01 | Röhr et al. |
| hsa-miR-548j-5p | 0.709 | 2.482 | 5.125E-01 | Röhr et al. |
| hsa-miR-3690 | -1.066 | 1.969 | 5.125E-01 | Röhr et al. |
| hsa-miR-181d-5p | 0.599 | 8.007 | 5.125E-01 | Röhr et al. |
| hsa-miR-21-3p | 0.660 | 7.020 | 5.125E-01 | Röhr et al. |
| hsa-miR-196a-5p | -1.070 | 8.583 | 5.163E-01 | Röhr et al. |
| hsa-miR-34a-3p | 0.776 | 2.714 | 5.222E-01 | Röhr et al. |
| hsa-miR-204-5p | -0.989 | 1.245 | 5.222E-01 | Röhr et al. |
| hsa-miR-6125 | -0.871 | 1.699 | 5.222E-01 | Röhr et al. |
| hsa-miR-28-3p | -0.784 | 7.531 | 5.222E-01 | Röhr et al. |
| hsa-miR-629-5p | 0.700 | 3.694 | 5.222E-01 | Röhr et al. |
| hsa-miR-643 | 0.718 | 0.473 | 5.222E-01 | Röhr et al. |
| hsa-miR-320c | 0.934 | 5.799 | 5.222E-01 | Röhr et al. |
| hsa-miR-10b-5p | -1.030 | 8.597 | 5.222E-01 | Röhr et al. |
| hsa-miR-217 | 0.896 | 2.410 | 5.222E-01 | Röhr et al. |
| hsa-miR-486-5p | -0.999 | 5.309 | 5.222E-01 | Röhr et al. |
| hsa-miR-542-5p | 0.993 | 0.698 | 5.222E-01 | Röhr et al. |
| hsa-miR-27a-3p | 0.754 | 10.011 | 5.222E-01 | Röhr et al. |
| hsa-miR-345-5p | 0.827 | 3.823 | 5.222E-01 | Röhr et al. |
| hsa-miR-877-5p | 0.883 | 3.474 | 5.222E-01 | Röhr et al. |
| hsa-miR-3610 | 0.719 | 1.136 | 5.222E-01 | Röhr et al. |
| hsa-miR-769-3p | 0.887 | 0.746 | 5.222E-01 | Röhr et al. |
| hsa-miR-24-3p | 0.692 | 9.822 | 5.222E-01 | Röhr et al. |
| hsa-miR-1246 | 1.363 | 6.179 | 5.222E-01 | Röhr et al. |
| hsa-miR-5187-5p | -0.832 | 0.201 | 5.222E-01 | Röhr et al. |
| hsa-miR-17-5p | 0.783 | 8.425 | 5.222E-01 | Röhr et al. |
| hsa-miR-24-2-5p | 0.639 | 4.656 | 5.222E-01 | Röhr et al. |
| hsa-miR-598-3p | -0.721 | 6.644 | 5.222E-01 | Röhr et al. |
| hsa-miR-500a-3p | 0.583 | 5.841 | 5.222E-01 | Röhr et al. |
| hsa-miR-296-5p | -0.999 | 0.612 | 5.222E-01 | Röhr et al. |
| hsa-miR-329-3p | -0.668 | 2.337 | 5.222E-01 | Röhr et al. |
| hsa-miR-193b-3p | -0.627 | 6.365 | 5.301E-01 | Röhr et al. |
| hsa-miR-1248 | 0.814 | 1.555 | 5.301E-01 | Röhr et al. |
| hsa-miR-501-5p | 0.868 | 1.463 | 5.301E-01 | Röhr et al. |
| hsa-miR-378d | -0.905 | 6.799 | 5.301E-01 | Röhr et al. |
| hsa-miR-29c-5p | -0.799 | 3.874 | 5.333E-01 | Röhr et al. |
| hsa-miR-9-3p | -0.996 | 2.820 | 5.339E-01 | Röhr et al. |
| hsa-miR-9-5p | -1.069 | 4.452 | 5.406E-01 | Röhr et al. |
| hsa-miR-92a-3p | 0.489 | 11.691 | 5.494E-01 | Röhr et al. |
| hsa-miR-455-5p | 0.605 | 5.196 | 5.501E-01 | Röhr et al. |
| hsa-miR-144-5p | -0.696 | 6.237 | 5.678E-01 | Röhr et al. |
| hsa-miR-214-5p | 0.870 | 0.585 | 5.678E-01 | Röhr et al. |
| hsa-miR-548a-3p | -0.712 | 1.750 | 5.678E-01 | Röhr et al. |
| hsa-miR-29b-3p | 0.769 | 10.222 | 5.717E-01 | Röhr et al. |
| hsa-miR-1306-3p | 0.640 | 1.406 | 5.773E-01 | Röhr et al. |
| hsa-miR-95-3p | 1.002 | 4.978 | 5.950E-01 | Röhr et al. |
| hsa-miR-502-3p | 0.544 | 6.060 | 5.952E-01 | Röhr et al. |
| hsa-miR-411-5p | -0.667 | 5.032 | 5.989E-01 | Röhr et al. |
| hsa-miR-138-5p | -0.888 | 4.078 | 5.989E-01 | Röhr et al. |
| hsa-miR-93-3p | 0.543 | 2.064 | 5.989E-01 | Röhr et al. |
| hsa-miR-335-5p | 0.705 | 6.850 | 5.989E-01 | Röhr et al. |
| hsa-miR-106b-5p | 0.888 | 8.771 | 5.989E-01 | Röhr et al. |
| hsa-miR-181b-3p | 0.674 | 0.743 | 5.989E-01 | Röhr et al. |
| hsa-miR-125a-5p | -0.799 | 7.924 | 5.989E-01 | Röhr et al. |
| hsa-miR-299-5p | 0.811 | 3.276 | 5.989E-01 | Röhr et al. |
| hsa-miR-99a-3p | -0.905 | 1.936 | 5.989E-01 | Röhr et al. |
| hsa-miR-181c-3p | 0.734 | 2.862 | 5.989E-01 | Röhr et al. |
| hsa-let-7b-5p | 0.554 | 14.745 | 5.989E-01 | Röhr et al. |
| hsa-miR-99b-3p | -0.913 | 3.072 | 5.989E-01 | Röhr et al. |
| hsa-miR-363-3p | -0.911 | 5.902 | 5.989E-01 | Röhr et al. |
| hsa-miR-219b-3p | 0.616 | 1.148 | 5.989E-01 | Röhr et al. |
| hsa-miR-25-5p | 0.687 | 6.166 | 5.989E-01 | Röhr et al. |
| hsa-miR-450a-5p | 0.973 | 2.318 | 5.989E-01 | Röhr et al. |
| hsa-miR-378f | -0.798 | 3.276 | 5.989E-01 | Röhr et al. |
| hsa-miR-450a-2-3p | 0.708 | 1.477 | 5.989E-01 | Röhr et al. |
| hsa-miR-30c-2-3p | -0.914 | 3.739 | 5.989E-01 | Röhr et al. |
| hsa-miR-451a | -0.835 | 9.828 | 5.989E-01 | Röhr et al. |
| hsa-miR-181a-2-3p | 0.635 | 6.403 | 6.028E-01 | Röhr et al. |
| hsa-miR-487b-3p | -0.544 | 5.397 | 6.028E-01 | Röhr et al. |
| hsa-let-7i-3p | 0.607 | 2.928 | 6.081E-01 | Röhr et al. |
| hsa-miR-1247-3p | -0.828 | 0.075 | 6.081E-01 | Röhr et al. |
| hsa-miR-378a-5p | -0.657 | 2.616 | 6.081E-01 | Röhr et al. |
| hsa-miR-324-5p | 0.645 | 4.740 | 6.081E-01 | Röhr et al. |
| hsa-miR-574-5p | 0.823 | 1.358 | 6.177E-01 | Röhr et al. |
| hsa-miR-328-3p | -0.750 | 1.423 | 6.201E-01 | Röhr et al. |
| hsa-miR-624-5p | 0.604 | 0.734 | 6.201E-01 | Röhr et al. |
| hsa-miR-887-3p | -0.685 | 3.706 | 6.234E-01 | Röhr et al. |
| hsa-miR-18a-5p | 1.060 | 4.693 | 6.293E-01 | Röhr et al. |
| hsa-miR-99b-5p | -0.722 | 8.677 | 6.293E-01 | Röhr et al. |
| hsa-miR-136-3p | -0.612 | 3.308 | 6.293E-01 | Röhr et al. |
| hsa-miR-1291 | -0.679 | 3.261 | 6.373E-01 | Röhr et al. |
| hsa-miR-27a-5p | 0.585 | 2.585 | 6.373E-01 | Röhr et al. |
| hsa-miR-3136-5p | 0.510 | 0.575 | 6.373E-01 | Röhr et al. |
| hsa-miR-542-3p | 0.638 | 3.255 | 6.409E-01 | Röhr et al. |
| hsa-miR-500a-5p | 1.231 | 2.429 | 6.431E-01 | Röhr et al. |
| hsa-miR-500b-5p | 1.231 | 2.429 | 6.431E-01 | Röhr et al. |
| hsa-miR-19a-3p | 0.790 | 1.875 | 6.677E-01 | Röhr et al. |
| hsa-miR-3131 | -0.844 | 2.069 | 6.677E-01 | Röhr et al. |
| hsa-miR-433-3p | -0.573 | 3.697 | 6.677E-01 | Röhr et al. |
| hsa-miR-501-3p | 0.467 | 3.159 | 6.677E-01 | Röhr et al. |
| hsa-miR-139-5p | -0.642 | 4.482 | 6.677E-01 | Röhr et al. |
| hsa-miR-210-3p | 0.622 | 7.178 | 6.677E-01 | Röhr et al. |
| hsa-miR-192-3p | -0.542 | 4.244 | 6.677E-01 | Röhr et al. |
| hsa-miR-23a-3p | 0.415 | 12.391 | 6.677E-01 | Röhr et al. |
| hsa-miR-4485-3p | 0.504 | 1.058 | 6.677E-01 | Röhr et al. |
| hsa-miR-29b-1-5p | 0.559 | 1.365 | 6.677E-01 | Röhr et al. |
| hsa-miR-25-3p | 0.406 | 12.404 | 6.677E-01 | Röhr et al. |
| hsa-miR-130b-3p | 0.450 | 6.782 | 6.677E-01 | Röhr et al. |
| hsa-miR-505-5p | 0.770 | 3.734 | 6.677E-01 | Röhr et al. |
| hsa-miR-374a-5p | 0.524 | 6.824 | 6.677E-01 | Röhr et al. |
| hsa-miR-130a-3p | -0.483 | 8.272 | 6.677E-01 | Röhr et al. |
| hsa-miR-374b-3p | 0.560 | 2.625 | 6.677E-01 | Röhr et al. |
| hsa-miR-320b | 0.580 | 7.832 | 6.677E-01 | Röhr et al. |
| hsa-miR-144-3p | -0.666 | 4.293 | 6.677E-01 | Röhr et al. |
| hsa-miR-106a-5p | 0.901 | 4.080 | 6.757E-01 | Röhr et al. |
| hsa-miR-212-5p | 0.503 | 2.773 | 6.757E-01 | Röhr et al. |
| hsa-miR-708-5p | 0.524 | 5.961 | 6.757E-01 | Röhr et al. |
| hsa-miR-142-5p | -0.568 | 10.117 | 6.757E-01 | Röhr et al. |
| hsa-miR-1247-5p | -0.778 | 1.656 | 6.796E-01 | Röhr et al. |
| hsa-miR-493-5p | 0.684 | 2.612 | 6.796E-01 | Röhr et al. |
| hsa-miR-625-5p | 0.508 | 4.242 | 6.796E-01 | Röhr et al. |
| hsa-miR-30a-3p | -0.574 | 6.679 | 6.796E-01 | Röhr et al. |
| hsa-miR-24-1-5p | 0.460 | 2.750 | 6.796E-01 | Röhr et al. |
| hsa-miR-577 | 0.704 | 4.157 | 6.796E-01 | Röhr et al. |
| hsa-miR-3648 | 0.596 | 1.355 | 6.796E-01 | Röhr et al. |
| hsa-miR-378e | 0.720 | 0.075 | 6.796E-01 | Röhr et al. |
| hsa-miR-504-5p | -0.720 | 3.465 | 6.796E-01 | Röhr et al. |
| hsa-miR-3614-5p | -0.514 | 0.344 | 6.796E-01 | Röhr et al. |
| hsa-miR-10a-5p | 0.427 | 13.607 | 6.796E-01 | Röhr et al. |
| hsa-miR-376c-3p | -0.547 | 5.441 | 6.796E-01 | Röhr et al. |
| hsa-miR-181c-5p | 0.455 | 5.879 | 6.796E-01 | Röhr et al. |
| hsa-miR-766-3p | -0.599 | 2.604 | 6.796E-01 | Röhr et al. |
| hsa-miR-23b-3p | 0.396 | 11.219 | 6.796E-01 | Röhr et al. |
| hsa-miR-582-3p | 0.542 | 2.273 | 6.796E-01 | Röhr et al. |
| hsa-miR-337-5p | -0.614 | 0.921 | 6.796E-01 | Röhr et al. |
| hsa-miR-195-5p | -0.661 | 7.985 | 6.796E-01 | Röhr et al. |
| hsa-miR-199b-5p | -0.538 | 5.715 | 6.796E-01 | Röhr et al. |
| hsa-miR-29a-5p | 0.542 | 3.782 | 6.796E-01 | Röhr et al. |
| hsa-miR-6882-5p | 0.451 | 0.904 | 6.796E-01 | Röhr et al. |
| hsa-miR-423-5p | 0.401 | 10.039 | 6.796E-01 | Röhr et al. |
| hsa-miR-1290 | 0.820 | 1.297 | 6.796E-01 | Röhr et al. |
| hsa-miR-1260a | -0.855 | 0.345 | 6.810E-01 | Röhr et al. |
| hsa-miR-20b-5p | -0.621 | 2.080 | 6.817E-01 | Röhr et al. |
| hsa-miR-7846-3p | -0.571 | 0.817 | 6.817E-01 | Röhr et al. |
| hsa-miR-8072 | 0.605 | 1.346 | 6.819E-01 | Röhr et al. |
| hsa-miR-184 | -0.673 | -0.119 | 6.827E-01 | Röhr et al. |
| hsa-miR-93-5p | 0.453 | 10.020 | 6.876E-01 | Röhr et al. |
| hsa-miR-196b-3p | -0.831 | 2.365 | 6.876E-01 | Röhr et al. |
| hsa-miR-1285-3p | 0.449 | 0.625 | 7.016E-01 | Röhr et al. |
| hsa-miR-151a-5p | 0.418 | 7.133 | 7.016E-01 | Röhr et al. |
| hsa-miR-487a-3p | 0.531 | 1.027 | 7.016E-01 | Röhr et al. |
| hsa-let-7a-3p | -0.486 | 2.796 | 7.054E-01 | Röhr et al. |
| hsa-miR-339-5p | 0.354 | 4.188 | 7.164E-01 | Röhr et al. |
| hsa-miR-6503-5p | -0.562 | 1.369 | 7.233E-01 | Röhr et al. |
| hsa-miR-511-5p | -0.551 | 0.426 | 7.233E-01 | Röhr et al. |
| hsa-miR-589-3p | 0.523 | 0.883 | 7.233E-01 | Röhr et al. |
| hsa-miR-493-3p | 0.580 | 0.665 | 7.233E-01 | Röhr et al. |
| hsa-miR-34a-5p | 0.498 | 5.073 | 7.293E-01 | Röhr et al. |
| hsa-miR-940 | 0.469 | 0.856 | 7.293E-01 | Röhr et al. |
| hsa-miR-486-3p | 0.536 | 0.743 | 7.293E-01 | Röhr et al. |
| hsa-miR-151a-3p | 0.339 | 8.037 | 7.293E-01 | Röhr et al. |
| hsa-let-7f-5p | 0.293 | 17.092 | 7.302E-01 | Röhr et al. |
| hsa-miR-4647 | 0.478 | 0.316 | 7.358E-01 | Röhr et al. |
| hsa-miR-454-3p | 0.564 | 1.803 | 7.384E-01 | Röhr et al. |
| hsa-miR-539-3p | -0.442 | 3.250 | 7.449E-01 | Röhr et al. |
| hsa-miR-140-3p | -0.437 | 13.080 | 7.449E-01 | Röhr et al. |
| hsa-miR-548n | 0.511 | 0.686 | 7.450E-01 | Röhr et al. |
| hsa-miR-659-5p | 0.423 | 1.004 | 7.450E-01 | Röhr et al. |
| hsa-miR-99a-5p | -0.618 | 8.288 | 7.450E-01 | Röhr et al. |
| hsa-miR-135a-5p | -0.460 | 0.388 | 7.450E-01 | Röhr et al. |
| hsa-miR-3614-3p | -0.524 | 1.622 | 7.450E-01 | Röhr et al. |
| hsa-miR-543 | -0.491 | 3.938 | 7.450E-01 | Röhr et al. |
| hsa-miR-4286 | 0.421 | 3.121 | 7.450E-01 | Röhr et al. |
| hsa-miR-190a-3p | -0.687 | 0.552 | 7.450E-01 | Röhr et al. |
| hsa-miR-199b-3p | -0.392 | 14.981 | 7.450E-01 | Röhr et al. |
| hsa-miR-199a-3p | -0.392 | 14.981 | 7.450E-01 | Röhr et al. |
| hsa-miR-1255b-5p | -0.468 | -0.016 | 7.450E-01 | Röhr et al. |
| hsa-miR-361-5p | 0.465 | 5.975 | 7.450E-01 | Röhr et al. |
| hsa-miR-15b-5p | 0.359 | 8.069 | 7.450E-01 | Röhr et al. |
| hsa-miR-221-5p | -0.312 | 8.549 | 7.455E-01 | Röhr et al. |
| hsa-miR-181a-3p | 0.424 | 4.972 | 7.455E-01 | Röhr et al. |
| hsa-miR-194-5p | -0.395 | 11.349 | 7.455E-01 | Röhr et al. |
| hsa-miR-671-5p | 0.387 | 2.135 | 7.487E-01 | Röhr et al. |
| hsa-miR-28-5p | -0.359 | 7.740 | 7.487E-01 | Röhr et al. |
| hsa-miR-1255a | 0.363 | 4.667 | 7.487E-01 | Röhr et al. |
| hsa-let-7b-3p | -0.404 | 2.796 | 7.516E-01 | Röhr et al. |
| hsa-miR-377-3p | 0.535 | 3.022 | 7.516E-01 | Röhr et al. |
| hsa-miR-410-3p | 0.385 | 3.679 | 7.516E-01 | Röhr et al. |
| hsa-miR-29c-3p | -0.410 | 10.973 | 7.530E-01 | Röhr et al. |
| hsa-miR-423-3p | 0.339 | 7.783 | 7.530E-01 | Röhr et al. |
| hsa-miR-150-5p | -0.689 | 3.526 | 7.732E-01 | Röhr et al. |
| hsa-miR-340-5p | 0.353 | 9.129 | 7.732E-01 | Röhr et al. |
| hsa-miR-494-3p | 0.453 | 3.455 | 7.797E-01 | Röhr et al. |
| hsa-miR-32-5p | 0.554 | 5.058 | 7.797E-01 | Röhr et al. |
| hsa-miR-148b-5p | 0.330 | 3.890 | 7.797E-01 | Röhr et al. |
| hsa-miR-548k | 0.287 | 3.721 | 7.867E-01 | Röhr et al. |
| hsa-miR-103a-2-5p | 0.393 | 1.325 | 7.867E-01 | Röhr et al. |
| hsa-miR-150-3p | -0.626 | 1.429 | 7.921E-01 | Röhr et al. |
| hsa-miR-676-3p | -0.454 | 0.501 | 8.024E-01 | Röhr et al. |
| hsa-miR-1278 | 0.303 | 3.596 | 8.038E-01 | Röhr et al. |
| hsa-miR-424-5p | 0.391 | 7.627 | 8.038E-01 | Röhr et al. |
| hsa-miR-125b-2-3p | -0.440 | 0.622 | 8.038E-01 | Röhr et al. |
| hsa-miR-4724-5p | 0.444 | 0.553 | 8.038E-01 | Röhr et al. |
| hsa-miR-125b-5p | -0.416 | 8.023 | 8.038E-01 | Röhr et al. |
| hsa-miR-140-5p | -0.353 | 2.553 | 8.038E-01 | Röhr et al. |
| hsa-let-7c-5p | 0.403 | 12.051 | 8.038E-01 | Röhr et al. |
| hsa-miR-3182 | -0.512 | 1.571 | 8.038E-01 | Röhr et al. |
| hsa-miR-655-3p | -0.337 | 2.782 | 8.038E-01 | Röhr et al. |
| hsa-miR-424-3p | 0.412 | 4.509 | 8.077E-01 | Röhr et al. |
| hsa-miR-376a-3p | -0.332 | 1.509 | 8.077E-01 | Röhr et al. |
| hsa-miR-708-3p | 0.361 | 1.550 | 8.096E-01 | Röhr et al. |
| hsa-miR-652-5p | 0.471 | 2.145 | 8.118E-01 | Röhr et al. |
| hsa-miR-10a-3p | 0.312 | 5.562 | 8.145E-01 | Röhr et al. |
| hsa-miR-4442 | 0.331 | 1.953 | 8.149E-01 | Röhr et al. |
| hsa-miR-34b-5p | 0.565 | 0.797 | 8.162E-01 | Röhr et al. |
| hsa-miR-3158-3p | 0.283 | 0.800 | 8.162E-01 | Röhr et al. |
| hsa-miR-16-5p | 0.314 | 10.513 | 8.162E-01 | Röhr et al. |
| hsa-miR-22-3p | 0.266 | 10.904 | 8.164E-01 | Röhr et al. |
| hsa-miR-4508 | -0.517 | 3.798 | 8.183E-01 | Röhr et al. |
| hsa-miR-221-3p | -0.302 | 11.868 | 8.277E-01 | Röhr et al. |
| hsa-miR-151b | 0.322 | 2.820 | 8.277E-01 | Röhr et al. |
| hsa-miR-3910 | -0.442 | 1.002 | 8.302E-01 | Röhr et al. |
| hsa-miR-641 | 0.328 | 3.101 | 8.302E-01 | Röhr et al. |
| hsa-miR-4791 | 0.326 | 2.074 | 8.302E-01 | Röhr et al. |
| hsa-miR-552-3p | -0.576 | 4.432 | 8.302E-01 | Röhr et al. |
| hsa-miR-3065-5p | -0.321 | 0.606 | 8.302E-01 | Röhr et al. |
| hsa-miR-191-3p | -0.280 | 1.680 | 8.302E-01 | Röhr et al. |
| hsa-miR-19b-3p | 0.464 | 6.660 | 8.314E-01 | Röhr et al. |
| hsa-miR-224-3p | 0.283 | 2.334 | 8.353E-01 | Röhr et al. |
| hsa-miR-664b-3p | 0.367 | 0.541 | 8.353E-01 | Röhr et al. |
| hsa-miR-98-5p | 0.321 | 7.737 | 8.353E-01 | Röhr et al. |
| hsa-miR-193a-3p | 0.332 | 7.223 | 8.398E-01 | Röhr et al. |
| hsa-miR-26a-5p | -0.258 | 12.157 | 8.398E-01 | Röhr et al. |
| hsa-let-7i-5p | 0.208 | 11.949 | 8.398E-01 | Röhr et al. |
| hsa-miR-592 | -0.542 | 0.672 | 8.398E-01 | Röhr et al. |
| hsa-miR-576-3p | -0.318 | 3.055 | 8.398E-01 | Röhr et al. |
| hsa-miR-200a-5p | -0.352 | 3.277 | 8.398E-01 | Röhr et al. |
| hsa-miR-324-3p | 0.250 | 0.851 | 8.398E-01 | Röhr et al. |
| hsa-miR-200a-3p | -0.290 | 10.910 | 8.398E-01 | Röhr et al. |
| hsa-miR-7-5p | 0.525 | 7.208 | 8.398E-01 | Röhr et al. |
| hsa-miR-339-3p | 0.266 | 3.494 | 8.398E-01 | Röhr et al. |
| hsa-miR-576-5p | -0.243 | 2.983 | 8.398E-01 | Röhr et al. |
| hsa-miR-3130-3p | -0.375 | 0.421 | 8.404E-01 | Röhr et al. |
| hsa-miR-193a-5p | -0.334 | 4.696 | 8.407E-01 | Röhr et al. |
| hsa-miR-4488 | 0.454 | 2.689 | 8.426E-01 | Röhr et al. |
| hsa-miR-331-5p | 0.279 | 0.748 | 8.426E-01 | Röhr et al. |
| hsa-miR-369-3p | 0.247 | 4.401 | 8.426E-01 | Röhr et al. |
| hsa-miR-362-3p | -0.306 | 4.353 | 8.426E-01 | Röhr et al. |
| hsa-miR-148b-3p | 0.281 | 6.343 | 8.426E-01 | Röhr et al. |
| hsa-miR-942-5p | -0.252 | 1.257 | 8.426E-01 | Röhr et al. |
| hsa-miR-532-3p | -0.282 | 3.767 | 8.426E-01 | Röhr et al. |
| hsa-miR-4430 | -0.389 | 1.389 | 8.426E-01 | Röhr et al. |
| hsa-miR-556-3p | -0.248 | 1.332 | 8.426E-01 | Röhr et al. |
| hsa-miR-126-3p | 0.263 | 5.420 | 8.426E-01 | Röhr et al. |
| hsa-miR-4510 | 0.251 | 1.299 | 8.426E-01 | Röhr et al. |
| hsa-miR-323a-3p | -0.343 | 3.586 | 8.426E-01 | Röhr et al. |
| hsa-miR-34c-5p | 0.293 | 6.442 | 8.426E-01 | Röhr et al. |
| hsa-miR-212-3p | 0.353 | 1.580 | 8.448E-01 | Röhr et al. |
| hsa-miR-342-3p | -0.282 | 7.307 | 8.448E-01 | Röhr et al. |
| hsa-miR-132-5p | -0.258 | 3.257 | 8.448E-01 | Röhr et al. |
| hsa-miR-200b-3p | -0.262 | 10.849 | 8.448E-01 | Röhr et al. |
| hsa-miR-377-5p | -0.264 | 0.866 | 8.451E-01 | Röhr et al. |
| hsa-miR-335-3p | 0.433 | 2.350 | 8.502E-01 | Röhr et al. |
| hsa-miR-106b-3p | 0.365 | 3.984 | 8.502E-01 | Röhr et al. |
| hsa-miR-4446-3p | 0.414 | 1.064 | 8.506E-01 | Röhr et al. |
| hsa-miR-484 | -0.284 | 3.188 | 8.506E-01 | Röhr et al. |
| hsa-miR-548e-5p | 0.293 | 0.240 | 8.506E-01 | Röhr et al. |
| hsa-miR-331-3p | 0.280 | 5.482 | 8.516E-01 | Röhr et al. |
| hsa-miR-6513-5p | 0.364 | 0.370 | 8.628E-01 | Röhr et al. |
| hsa-miR-146b-3p | -0.340 | 1.755 | 8.632E-01 | Röhr et al. |
| hsa-miR-362-5p | 0.246 | 5.241 | 8.787E-01 | Röhr et al. |
| hsa-miR-365b-5p | -0.278 | 3.445 | 8.787E-01 | Röhr et al. |
| hsa-miR-30e-3p | 0.218 | 7.497 | 8.787E-01 | Röhr et al. |
| hsa-miR-485-3p | -0.285 | 3.330 | 8.787E-01 | Röhr et al. |
| hsa-miR-372-3p | 0.245 | 0.565 | 8.787E-01 | Röhr et al. |
| hsa-miR-20b-3p | -0.420 | 0.605 | 8.787E-01 | Röhr et al. |
| hsa-miR-95-5p | 0.400 | 0.556 | 8.787E-01 | Röhr et al. |
| hsa-miR-4485-5p | 0.295 | 0.647 | 8.787E-01 | Röhr et al. |
| hsa-miR-2110 | -0.275 | 2.292 | 8.847E-01 | Röhr et al. |
| hsa-miR-6516-3p | 0.253 | 0.889 | 8.847E-01 | Röhr et al. |
| hsa-miR-342-5p | -0.315 | 4.044 | 8.847E-01 | Röhr et al. |
| hsa-miR-429 | -0.253 | 8.961 | 8.847E-01 | Röhr et al. |
| hsa-miR-126-5p | -0.208 | 6.381 | 8.857E-01 | Röhr et al. |
| hsa-miR-548t-5p | -0.259 | 1.344 | 8.857E-01 | Röhr et al. |
| hsa-miR-483-5p | 0.411 | 2.820 | 8.911E-01 | Röhr et al. |
| hsa-miR-191-5p | -0.172 | 12.657 | 8.911E-01 | Röhr et al. |
| hsa-let-7d-3p | -0.230 | 5.209 | 8.911E-01 | Röhr et al. |
| hsa-miR-15a-3p | 0.263 | 0.515 | 8.911E-01 | Röhr et al. |
| hsa-miR-660-5p | 0.238 | 7.023 | 8.911E-01 | Röhr et al. |
| hsa-miR-188-5p | 0.351 | 3.000 | 8.911E-01 | Röhr et al. |
| hsa-miR-625-3p | 0.248 | 4.277 | 8.911E-01 | Röhr et al. |
| hsa-miR-766-5p | -0.234 | 1.836 | 8.911E-01 | Röhr et al. |
| hsa-miR-155-5p | 0.227 | 7.521 | 8.911E-01 | Röhr et al. |
| hsa-miR-381-3p | -0.288 | 1.280 | 8.961E-01 | Röhr et al. |
| hsa-miR-548e-3p | -0.192 | 4.329 | 8.961E-01 | Röhr et al. |
| hsa-miR-190a-5p | -0.348 | 0.917 | 8.961E-01 | Röhr et al. |
| hsa-miR-16-2-3p | -0.202 | 3.088 | 8.961E-01 | Röhr et al. |
| hsa-miR-548l | 0.186 | 1.182 | 8.961E-01 | Röhr et al. |
| hsa-miR-30d-3p | 0.230 | 1.119 | 8.961E-01 | Röhr et al. |
| hsa-miR-425-5p | 0.241 | 8.703 | 8.961E-01 | Röhr et al. |
| hsa-miR-365a-5p | -0.194 | 3.147 | 8.961E-01 | Röhr et al. |
| hsa-miR-495-3p | -0.177 | 5.985 | 8.961E-01 | Röhr et al. |
| hsa-miR-1287-5p | 0.225 | 0.975 | 8.961E-01 | Röhr et al. |
| hsa-miR-4444 | -0.231 | 0.185 | 8.961E-01 | Röhr et al. |
| hsa-miR-1271-5p | 0.250 | 2.452 | 8.980E-01 | Röhr et al. |
| hsa-miR-152-3p | -0.164 | 9.388 | 8.980E-01 | Röhr et al. |
| hsa-miR-590-5p | 0.307 | 3.851 | 8.980E-01 | Röhr et al. |
| hsa-miR-301a-3p | -0.213 | 2.045 | 8.980E-01 | Röhr et al. |
| hsa-let-7g-5p | 0.153 | 13.834 | 8.980E-01 | Röhr et al. |
| hsa-miR-219a-1-3p | 0.210 | 1.329 | 8.980E-01 | Röhr et al. |
| hsa-miR-185-5p | 0.189 | 9.674 | 8.980E-01 | Röhr et al. |
| hsa-miR-3656 | 0.299 | 0.088 | 8.980E-01 | Röhr et al. |
| hsa-miR-654-3p | -0.220 | 2.887 | 9.032E-01 | Röhr et al. |
| hsa-miR-3917 | 0.214 | 0.724 | 9.032E-01 | Röhr et al. |
| hsa-miR-92b-3p | 0.180 | 7.265 | 9.032E-01 | Röhr et al. |
| hsa-miR-4516 | 0.194 | 4.393 | 9.124E-01 | Röhr et al. |
| hsa-miR-146a-5p | -0.221 | 8.199 | 9.124E-01 | Röhr et al. |
| hsa-miR-582-5p | 0.219 | 2.549 | 9.129E-01 | Röhr et al. |
| hsa-miR-2355-3p | 0.215 | 0.591 | 9.129E-01 | Röhr et al. |
| hsa-miR-1185-5p | -0.168 | 2.526 | 9.129E-01 | Röhr et al. |
| hsa-miR-200c-5p | -0.226 | 0.440 | 9.129E-01 | Röhr et al. |
| hsa-miR-382-3p | -0.190 | 5.282 | 9.129E-01 | Röhr et al. |
| hsa-miR-148a-3p | -0.205 | 10.468 | 9.129E-01 | Röhr et al. |
| hsa-miR-30e-5p | -0.150 | 9.083 | 9.131E-01 | Röhr et al. |
| hsa-miR-374b-5p | 0.154 | 7.884 | 9.131E-01 | Röhr et al. |
| hsa-miR-548o-3p | 0.218 | 0.580 | 9.131E-01 | Röhr et al. |
| hsa-miR-532-5p | 0.134 | 8.366 | 9.168E-01 | Röhr et al. |
| hsa-miR-223-3p | 0.202 | 7.587 | 9.172E-01 | Röhr et al. |
| hsa-miR-505-3p | -0.172 | 3.130 | 9.193E-01 | Röhr et al. |
| hsa-miR-769-5p | 0.204 | 1.630 | 9.193E-01 | Röhr et al. |
| hsa-miR-1301-3p | 0.227 | 2.533 | 9.203E-01 | Röhr et al. |
| hsa-miR-323b-3p | -0.299 | 0.135 | 9.203E-01 | Röhr et al. |
| hsa-miR-3129-5p | 0.155 | 0.385 | 9.203E-01 | Röhr et al. |
| hsa-miR-1307-3p | -0.176 | 6.833 | 9.203E-01 | Röhr et al. |
| hsa-miR-539-5p | -0.242 | -0.058 | 9.203E-01 | Röhr et al. |
| hsa-miR-889-3p | -0.184 | 5.307 | 9.203E-01 | Röhr et al. |
| hsa-miR-200b-5p | -0.177 | 8.759 | 9.203E-01 | Röhr et al. |
| hsa-miR-590-3p | 0.190 | 2.428 | 9.203E-01 | Röhr et al. |
| hsa-miR-656-3p | 0.178 | 0.327 | 9.245E-01 | Röhr et al. |
| hsa-miR-652-3p | 0.159 | 5.259 | 9.253E-01 | Röhr et al. |
| hsa-miR-29b-2-5p | -0.153 | 2.616 | 9.253E-01 | Röhr et al. |
| hsa-miR-7704 | -0.208 | 3.657 | 9.253E-01 | Röhr et al. |
| hsa-miR-125b-1-3p | -0.197 | 0.630 | 9.253E-01 | Röhr et al. |
| hsa-miR-382-5p | 0.181 | 5.967 | 9.253E-01 | Röhr et al. |
| hsa-miR-3127-5p | 0.181 | 1.826 | 9.253E-01 | Röhr et al. |
| hsa-miR-30c-5p | -0.138 | 7.222 | 9.253E-01 | Röhr et al. |
| hsa-miR-33a-3p | -0.160 | 2.255 | 9.253E-01 | Röhr et al. |
| hsa-miR-374a-3p | 0.142 | 7.711 | 9.253E-01 | Röhr et al. |
| hsa-miR-148a-5p | -0.165 | 3.942 | 9.253E-01 | Röhr et al. |
| hsa-miR-3605-5p | 0.160 | 2.183 | 9.253E-01 | Röhr et al. |
| hsa-miR-6503-3p | 0.161 | 0.682 | 9.253E-01 | Röhr et al. |
| hsa-miR-30c-1-3p | -0.146 | 2.659 | 9.253E-01 | Röhr et al. |
| hsa-miR-1273c | -0.152 | 0.309 | 9.253E-01 | Röhr et al. |
| hsa-miR-127-3p | -0.136 | 6.508 | 9.253E-01 | Röhr et al. |
| hsa-miR-3613-3p | -0.133 | 1.524 | 9.253E-01 | Röhr et al. |
| hsa-miR-1275 | 0.199 | 1.515 | 9.269E-01 | Röhr et al. |
| hsa-miR-185-3p | -0.150 | 1.680 | 9.284E-01 | Röhr et al. |
| hsa-miR-7-1-3p | -0.119 | 4.814 | 9.284E-01 | Röhr et al. |
| hsa-miR-146b-5p | -0.158 | 10.572 | 9.284E-01 | Röhr et al. |
| hsa-miR-23b-5p | 0.153 | 5.648 | 9.313E-01 | Röhr et al. |
| hsa-miR-944 | -0.187 | 0.995 | 9.331E-01 | Röhr et al. |
| hsa-miR-15a-5p | 0.151 | 8.080 | 9.331E-01 | Röhr et al. |
| hsa-miR-33a-5p | 0.112 | 8.114 | 9.331E-01 | Röhr et al. |
| hsa-miR-32-3p | -0.128 | 1.148 | 9.331E-01 | Röhr et al. |
| hsa-miR-154-3p | 0.145 | 1.800 | 9.331E-01 | Röhr et al. |
| hsa-miR-203a-3p | -0.163 | 9.169 | 9.331E-01 | Röhr et al. |
| hsa-miR-1269a | -0.373 | 0.986 | 9.394E-01 | Röhr et al. |
| hsa-miR-3202 | 0.135 | 0.894 | 9.394E-01 | Röhr et al. |
| hsa-miR-589-5p | -0.124 | 1.722 | 9.394E-01 | Röhr et al. |
| hsa-miR-432-5p | -0.136 | 5.321 | 9.455E-01 | Röhr et al. |
| hsa-miR-1262 | -0.144 | 3.097 | 9.455E-01 | Röhr et al. |
| hsa-miR-874-3p | 0.115 | 2.170 | 9.455E-01 | Röhr et al. |
| hsa-miR-379-5p | -0.112 | 3.718 | 9.455E-01 | Röhr et al. |
| hsa-miR-103a-3p | 0.087 | 14.858 | 9.455E-01 | Röhr et al. |
| hsa-miR-4492 | -0.162 | 1.588 | 9.459E-01 | Röhr et al. |
| hsa-miR-214-3p | -0.109 | 6.268 | 9.459E-01 | Röhr et al. |
| hsa-miR-3934-5p | 0.111 | 1.130 | 9.473E-01 | Röhr et al. |
| hsa-miR-197-3p | 0.095 | 4.331 | 9.473E-01 | Röhr et al. |
| hsa-miR-361-3p | 0.108 | 3.282 | 9.487E-01 | Röhr et al. |
| hsa-miR-200c-3p | -0.141 | 11.235 | 9.487E-01 | Röhr et al. |
| hsa-miR-132-3p | -0.101 | 3.285 | 9.487E-01 | Röhr et al. |
| hsa-miR-301a-5p | 0.112 | 2.807 | 9.487E-01 | Röhr et al. |
| hsa-miR-5091 | -0.101 | 3.416 | 9.487E-01 | Röhr et al. |
| hsa-miR-369-5p | 0.098 | 3.177 | 9.487E-01 | Röhr et al. |
| hsa-miR-30b-3p | -0.101 | 3.321 | 9.487E-01 | Röhr et al. |
| hsa-miR-664a-3p | -0.088 | 5.231 | 9.487E-01 | Röhr et al. |
| hsa-let-7e-3p | -0.088 | 1.963 | 9.488E-01 | Röhr et al. |
| hsa-miR-548h-5p | -0.138 | 0.060 | 9.488E-01 | Röhr et al. |
| hsa-miR-628-3p | -0.084 | -0.068 | 9.488E-01 | Röhr et al. |
| hsa-miR-550a-3p | 0.135 | 1.169 | 9.488E-01 | Röhr et al. |
| hsa-miR-409-5p | 0.143 | 2.792 | 9.488E-01 | Röhr et al. |
| hsa-miR-181b-5p | -0.096 | 9.381 | 9.488E-01 | Röhr et al. |
| hsa-miR-1294 | 0.103 | 0.653 | 9.488E-01 | Röhr et al. |
| hsa-miR-574-3p | 0.099 | 7.933 | 9.501E-01 | Röhr et al. |
| hsa-miR-127-5p | -0.082 | 3.090 | 9.501E-01 | Röhr et al. |
| hsa-miR-941 | 0.099 | 3.944 | 9.522E-01 | Röhr et al. |
| hsa-miR-223-5p | 0.088 | 3.452 | 9.522E-01 | Röhr et al. |
| hsa-miR-107 | 0.081 | 13.361 | 9.522E-01 | Röhr et al. |
| hsa-miR-6761-5p | -0.084 | 0.034 | 9.552E-01 | Röhr et al. |
| hsa-miR-660-3p | 0.095 | 2.283 | 9.552E-01 | Röhr et al. |
| hsa-miR-499a-5p | -0.113 | 5.305 | 9.552E-01 | Röhr et al. |
| hsa-miR-3200-5p | 0.089 | 0.435 | 9.571E-01 | Röhr et al. |
| hsa-miR-141-3p | 0.115 | 7.190 | 9.571E-01 | Röhr et al. |
| hsa-miR-125a-3p | -0.086 | 1.597 | 9.571E-01 | Röhr et al. |
| hsa-miR-628-5p | 0.068 | 2.716 | 9.571E-01 | Röhr et al. |
| hsa-miR-222-3p | -0.057 | 9.750 | 9.580E-01 | Röhr et al. |
| hsa-miR-22-5p | 0.061 | 6.436 | 9.580E-01 | Röhr et al. |
| hsa-miR-664a-5p | -0.094 | 6.288 | 9.580E-01 | Röhr et al. |
| hsa-miR-548v | 0.070 | 0.393 | 9.580E-01 | Röhr et al. |
| hsa-miR-1307-5p | 0.065 | 6.839 | 9.596E-01 | Röhr et al. |
| hsa-let-7e-5p | 0.063 | 11.643 | 9.596E-01 | Röhr et al. |
| hsa-miR-193b-5p | 0.125 | 3.434 | 9.635E-01 | Röhr et al. |
| hsa-miR-141-5p | 0.092 | 2.151 | 9.687E-01 | Röhr et al. |
| hsa-miR-218-1-3p | -0.071 | 0.540 | 9.687E-01 | Röhr et al. |
| hsa-miR-100-5p | -0.088 | 8.655 | 9.687E-01 | Röhr et al. |
| hsa-miR-1277-5p | -0.086 | 0.905 | 9.687E-01 | Röhr et al. |
| hsa-miR-651-5p | -0.050 | 1.443 | 9.687E-01 | Röhr et al. |
| hsa-miR-409-3p | 0.061 | 4.464 | 9.687E-01 | Röhr et al. |
| hsa-miR-1277-3p | -0.052 | 3.784 | 9.687E-01 | Röhr et al. |
| hsa-miR-365a-3p | -0.059 | 7.517 | 9.687E-01 | Röhr et al. |
| hsa-miR-365b-3p | -0.059 | 7.517 | 9.687E-01 | Röhr et al. |
| hsa-miR-5010-5p | -0.050 | 1.629 | 9.763E-01 | Röhr et al. |
| hsa-miR-4443 | 0.050 | 4.214 | 9.763E-01 | Röhr et al. |
| hsa-miR-548i | -0.098 | 0.534 | 9.763E-01 | Röhr et al. |
| hsa-miR-218-5p | -0.083 | 0.680 | 9.848E-01 | Röhr et al. |
| hsa-miR-136-5p | -0.041 | 4.796 | 9.848E-01 | Röhr et al. |
| hsa-miR-4497 | 0.059 | 3.101 | 9.848E-01 | Röhr et al. |
| hsa-miR-181a-5p | -0.044 | 11.185 | 9.848E-01 | Röhr et al. |
| hsa-miR-128-3p | -0.036 | 7.601 | 9.848E-01 | Röhr et al. |
| hsa-miR-4521 | 0.054 | 2.112 | 9.848E-01 | Röhr et al. |
| hsa-miR-664b-5p | 0.038 | 2.029 | 9.848E-01 | Röhr et al. |
| hsa-miR-1270 | -0.045 | 0.375 | 9.848E-01 | Röhr et al. |
| hsa-miR-376b-3p | -0.039 | 2.974 | 9.848E-01 | Röhr et al. |
| hsa-miR-411-3p | -0.039 | 0.515 | 9.848E-01 | Röhr et al. |
| hsa-miR-142-3p | 0.040 | 6.449 | 9.848E-01 | Röhr et al. |
| hsa-miR-425-3p | 0.026 | 5.448 | 9.848E-01 | Röhr et al. |
| hsa-miR-199a-5p | -0.039 | 6.105 | 9.848E-01 | Röhr et al. |
| hsa-miR-320e | 0.062 | 0.688 | 9.848E-01 | Röhr et al. |
| hsa-miR-3916 | -0.037 | 0.462 | 9.848E-01 | Röhr et al. |
| hsa-miR-2355-5p | 0.027 | 2.205 | 9.848E-01 | Röhr et al. |
| hsa-miR-26b-5p | 0.022 | 11.167 | 9.848E-01 | Röhr et al. |
| hsa-miR-154-5p | 0.031 | 2.057 | 9.848E-01 | Röhr et al. |
| hsa-miR-30b-5p | 0.024 | 6.444 | 9.848E-01 | Röhr et al. |
| hsa-miR-5588-5p | 0.036 | 1.280 | 9.848E-01 | Röhr et al. |
| hsa-miR-134-5p | 0.023 | 3.565 | 9.848E-01 | Röhr et al. |
| hsa-miR-15b-3p | -0.027 | 1.976 | 9.848E-01 | Röhr et al. |
| hsa-miR-30d-5p | -0.018 | 10.979 | 9.848E-01 | Röhr et al. |
| hsa-miR-320a | -0.016 | 13.308 | 9.848E-01 | Röhr et al. |
| hsa-miR-101-3p | 0.016 | 12.850 | 9.848E-01 | Röhr et al. |
| hsa-miR-3912-3p | -0.018 | 1.127 | 9.848E-01 | Röhr et al. |
| hsa-miR-186-5p | 0.015 | 9.232 | 9.848E-01 | Röhr et al. |
| hsa-miR-935 | 0.026 | 0.776 | 9.849E-01 | Röhr et al. |
| hsa-miR-3664-3p | 0.015 | 1.483 | 9.849E-01 | Röhr et al. |
| hsa-miR-371a-5p | 0.014 | 1.103 | 9.849E-01 | Röhr et al. |
| hsa-miR-744-5p | -0.016 | 4.952 | 9.849E-01 | Röhr et al. |
| hsa-miR-299-3p | 0.016 | 0.799 | 9.850E-01 | Röhr et al. |
| hsa-miR-33b-5p | 0.010 | 4.975 | 9.850E-01 | Röhr et al. |
| hsa-miR-3145-3p | 0.010 | 0.129 | 9.850E-01 | Röhr et al. |
| hsa-miR-330-3p | 0.006 | 7.548 | 9.945E-01 | Röhr et al. |
| hsa-miR-3928-3p | 0.004 | 2.902 | 9.945E-01 | Röhr et al. |
| hsa-miR-4419b | -0.003 | 0.230 | 9.954E-01 | Röhr et al. |
| hsa-miR-137 | -2.682 | 0.871 | 2.856E-12 | Sun et al. |
| hsa-miR-378c | -1.269 | 8.229 | 2.856E-12 | Sun et al. |
| hsa-miR-378a-3p | -1.402 | 11.868 | 1.033E-11 | Sun et al. |
| hsa-miR-135b-3p | 3.478 | 0.970 | 1.033E-11 | Sun et al. |
| hsa-miR-124-3p | -2.224 | 0.846 | 3.406E-11 | Sun et al. |
| hsa-miR-378a-5p | -1.415 | 5.703 | 8.733E-11 | Sun et al. |
| hsa-miR-378d | -1.253 | 5.398 | 2.902E-10 | Sun et al. |
| hsa-miR-503-5p | 1.881 | 3.104 | 3.374E-10 | Sun et al. |
| hsa-miR-31-5p | 2.960 | 4.195 | 6.231E-10 | Sun et al. |
| hsa-miR-135b-5p | 3.135 | 4.558 | 6.231E-10 | Sun et al. |
| hsa-miR-9-5p | -1.727 | 6.211 | 6.231E-10 | Sun et al. |
| hsa-miR-424-3p | 1.423 | 2.328 | 6.834E-10 | Sun et al. |
| hsa-miR-21-5p | 1.359 | 17.766 | 7.238E-10 | Sun et al. |
| hsa-miR-26a-5p | -0.751 | 13.663 | 1.389E-09 | Sun et al. |
| hsa-miR-9-3p | -1.511 | 4.949 | 1.442E-09 | Sun et al. |
| hsa-miR-30a-5p | -1.106 | 10.989 | 5.072E-09 | Sun et al. |
| hsa-miR-4517 | 1.947 | 0.277 | 1.835E-08 | Sun et al. |
| hsa-miR-708-5p | 1.311 | 6.230 | 1.835E-08 | Sun et al. |
| hsa-miR-493-5p | 1.234 | 2.825 | 4.732E-08 | Sun et al. |
| hsa-miR-139-5p | -1.683 | 6.989 | 8.779E-08 | Sun et al. |
| hsa-miR-224-5p | 1.386 | 6.011 | 9.900E-08 | Sun et al. |
| hsa-miR-129-5p | -2.255 | 1.279 | 1.348E-07 | Sun et al. |
| hsa-miR-139-3p | -1.475 | 1.029 | 2.363E-07 | Sun et al. |
| hsa-miR-497-5p | -1.021 | 6.981 | 4.305E-07 | Sun et al. |
| hsa-miR-450a-5p | 1.061 | 5.341 | 4.821E-07 | Sun et al. |
| hsa-miR-29b-1-5p | 1.130 | 0.842 | 5.347E-07 | Sun et al. |
| hsa-miR-138-5p | -1.128 | 2.903 | 6.755E-07 | Sun et al. |
| hsa-miR-195-5p | -0.937 | 9.024 | 6.927E-07 | Sun et al. |
| hsa-miR-450b-5p | 1.298 | 3.982 | 6.927E-07 | Sun et al. |
| hsa-miR-584-5p | 1.377 | 3.574 | 8.245E-07 | Sun et al. |
| hsa-miR-1185-1-3p | 1.020 | 1.560 | 8.245E-07 | Sun et al. |
| hsa-miR-424-5p | 1.211 | 7.045 | 8.245E-07 | Sun et al. |
| hsa-miR-29c-5p | -0.836 | 5.184 | 1.231E-06 | Sun et al. |
| hsa-miR-18a-3p | 0.928 | 1.845 | 1.485E-06 | Sun et al. |
| hsa-miR-10b-5p | -0.930 | 13.552 | 1.555E-06 | Sun et al. |
| hsa-miR-338-3p | -1.054 | 3.764 | 2.526E-06 | Sun et al. |
| hsa-miR-493-3p | 0.935 | 2.649 | 2.969E-06 | Sun et al. |
| hsa-miR-877-5p | 0.906 | 1.893 | 3.698E-06 | Sun et al. |
| hsa-miR-30c-2-3p | -1.080 | 3.760 | 3.698E-06 | Sun et al. |
| hsa-miR-21-3p | 1.110 | 7.540 | 4.451E-06 | Sun et al. |
| hsa-miR-708-3p | 1.101 | 2.510 | 8.869E-06 | Sun et al. |
| hsa-miR-30a-3p | -1.056 | 5.154 | 8.892E-06 | Sun et al. |
| hsa-miR-10b-3p | -0.871 | 4.824 | 8.950E-06 | Sun et al. |
| hsa-miR-26b-5p | -0.561 | 11.172 | 9.923E-06 | Sun et al. |
| hsa-miR-215-3p | -2.171 | 2.410 | 1.078E-05 | Sun et al. |
| hsa-miR-215-5p | -2.191 | 10.559 | 1.179E-05 | Sun et al. |
| hsa-miR-135a-5p | -1.619 | 2.125 | 1.457E-05 | Sun et al. |
| hsa-miR-335-3p | 0.971 | 6.173 | 1.687E-05 | Sun et al. |
| hsa-miR-338-5p | -1.185 | 5.133 | 2.006E-05 | Sun et al. |
| hsa-miR-182-5p | 1.410 | 8.581 | 2.392E-05 | Sun et al. |
| hsa-miR-26b-3p | -0.611 | 2.062 | 3.103E-05 | Sun et al. |
| hsa-miR-30e-3p | -0.644 | 6.838 | 3.146E-05 | Sun et al. |
| hsa-miR-129-2-3p | -1.591 | 0.531 | 3.344E-05 | Sun et al. |
| hsa-miR-183-5p | 1.472 | 7.283 | 4.302E-05 | Sun et al. |
| hsa-miR-30b-5p | -0.466 | 10.260 | 5.802E-05 | Sun et al. |
| hsa-miR-542-5p | 0.868 | 0.816 | 5.802E-05 | Sun et al. |
| hsa-miR-504-5p | -1.517 | 3.301 | 7.182E-05 | Sun et al. |
| hsa-miR-1-3p | -1.808 | 11.546 | 7.202E-05 | Sun et al. |
| hsa-let-7g-5p | -0.413 | 12.408 | 7.330E-05 | Sun et al. |
| hsa-miR-18a-5p | 1.147 | 7.852 | 7.763E-05 | Sun et al. |
| hsa-miR-29b-2-5p | -0.758 | 4.433 | 8.196E-05 | Sun et al. |
| hsa-miR-17-5p | 0.818 | 11.434 | 1.152E-04 | Sun et al. |
| hsa-miR-223-3p | 0.763 | 7.127 | 1.202E-04 | Sun et al. |
| hsa-miR-149-5p | -0.903 | 3.889 | 1.202E-04 | Sun et al. |
| hsa-miR-30c-5p | -0.644 | 10.618 | 1.327E-04 | Sun et al. |
| hsa-miR-92a-1-5p | 0.836 | 2.609 | 1.525E-04 | Sun et al. |
| hsa-miR-96-5p | 1.321 | 3.850 | 1.574E-04 | Sun et al. |
| hsa-miR-508-3p | 1.759 | 0.161 | 1.762E-04 | Sun et al. |
| hsa-miR-421 | 0.555 | 4.312 | 2.081E-04 | Sun et al. |
| hsa-miR-887-3p | -0.675 | 3.406 | 2.128E-04 | Sun et al. |
| hsa-miR-204-5p | -1.287 | 3.030 | 2.463E-04 | Sun et al. |
| hsa-miR-95-3p | 0.617 | 7.001 | 2.520E-04 | Sun et al. |
| hsa-miR-222-5p | 0.831 | 1.198 | 2.555E-04 | Sun et al. |
| hsa-miR-451a | -0.970 | 10.611 | 2.603E-04 | Sun et al. |
| hsa-miR-133a-5p | -1.585 | 2.603 | 2.867E-04 | Sun et al. |
| hsa-miR-133a-3p | -1.765 | 8.165 | 2.867E-04 | Sun et al. |
| hsa-miR-29c-3p | -0.821 | 7.340 | 3.405E-04 | Sun et al. |
| hsa-miR-134-5p | 0.589 | 3.863 | 3.534E-04 | Sun et al. |
| hsa-miR-542-3p | 1.018 | 7.354 | 3.601E-04 | Sun et al. |
| hsa-miR-30d-5p | -0.453 | 12.117 | 4.059E-04 | Sun et al. |
| hsa-miR-133b | -1.742 | 4.058 | 4.584E-04 | Sun et al. |
| hsa-miR-145-3p | -1.043 | 8.964 | 4.979E-04 | Sun et al. |
| hsa-miR-598-3p | -0.628 | 3.186 | 4.982E-04 | Sun et al. |
| hsa-miR-301b-3p | 1.071 | 0.542 | 5.278E-04 | Sun et al. |
| hsa-miR-28-3p | -0.691 | 9.667 | 5.278E-04 | Sun et al. |
| hsa-miR-25-5p | 0.794 | 1.458 | 5.735E-04 | Sun et al. |
| hsa-miR-217 | 1.049 | 3.046 | 6.077E-04 | Sun et al. |
| hsa-miR-181a-3p | 0.567 | 4.793 | 6.286E-04 | Sun et al. |
| hsa-miR-92a-3p | 0.503 | 11.027 | 1.061E-03 | Sun et al. |
| hsa-miR-3687 | 1.263 | 0.765 | 1.067E-03 | Sun et al. |
| hsa-miR-501-5p | 0.538 | 2.905 | 1.154E-03 | Sun et al. |
| hsa-miR-101-5p | -0.798 | 2.316 | 1.305E-03 | Sun et al. |
| hsa-miR-1185-2-3p | 0.736 | 0.413 | 1.347E-03 | Sun et al. |
| hsa-miR-145-5p | -1.329 | 12.553 | 1.392E-03 | Sun et al. |
| hsa-miR-6516-5p | 0.610 | 0.454 | 1.396E-03 | Sun et al. |
| hsa-miR-20a-5p | 0.841 | 12.291 | 1.426E-03 | Sun et al. |
| hsa-miR-34a-3p | 0.914 | 0.232 | 1.539E-03 | Sun et al. |
| hsa-miR-409-3p | 0.547 | 4.982 | 1.539E-03 | Sun et al. |
| hsa-miR-370-3p | 0.557 | 3.539 | 1.553E-03 | Sun et al. |
| hsa-miR-452-5p | 0.659 | 7.426 | 1.686E-03 | Sun et al. |
| hsa-miR-181d-5p | 0.585 | 4.392 | 1.974E-03 | Sun et al. |
| hsa-miR-186-5p | -0.260 | 10.994 | 2.110E-03 | Sun et al. |
| hsa-miR-758-3p | 0.602 | 2.399 | 2.161E-03 | Sun et al. |
| hsa-miR-452-3p | 0.793 | 3.164 | 2.208E-03 | Sun et al. |
| hsa-miR-106a-5p | 0.635 | 9.127 | 2.342E-03 | Sun et al. |
| hsa-miR-130a-3p | -0.445 | 6.629 | 2.821E-03 | Sun et al. |
| hsa-miR-1247-5p | 1.029 | 2.882 | 2.916E-03 | Sun et al. |
| hsa-miR-574-3p | -0.512 | 7.905 | 3.179E-03 | Sun et al. |
| hsa-miR-30b-3p | -0.437 | 1.585 | 3.834E-03 | Sun et al. |
| hsa-miR-190a-5p | -0.801 | 5.456 | 4.630E-03 | Sun et al. |
| hsa-miR-28-5p | -0.571 | 10.267 | 4.657E-03 | Sun et al. |
| hsa-miR-140-3p | -0.346 | 10.885 | 4.966E-03 | Sun et al. |
| hsa-miR-1275 | 0.935 | 1.364 | 4.966E-03 | Sun et al. |
| hsa-miR-195-3p | -0.786 | 1.171 | 4.966E-03 | Sun et al. |
| hsa-miR-30e-5p | -0.439 | 11.658 | 5.042E-03 | Sun et al. |
| hsa-miR-1-5p | -1.155 | -0.187 | 5.049E-03 | Sun et al. |
| hsa-miR-27a-3p | 0.334 | 10.377 | 5.486E-03 | Sun et al. |
| hsa-miR-27a-5p | 0.802 | 5.293 | 5.535E-03 | Sun et al. |
| hsa-miR-487a-3p | 0.599 | 1.503 | 5.596E-03 | Sun et al. |
| hsa-miR-7-5p | 0.961 | 10.551 | 5.605E-03 | Sun et al. |
| hsa-miR-320d | 0.612 | 2.644 | 6.112E-03 | Sun et al. |
| hsa-miR-218-5p | -0.737 | 7.483 | 6.638E-03 | Sun et al. |
| hsa-miR-188-5p | 0.892 | 1.186 | 6.788E-03 | Sun et al. |
| hsa-miR-4532 | -0.822 | 3.815 | 7.409E-03 | Sun et al. |
| hsa-miR-30c-1-3p | -0.420 | 1.939 | 7.409E-03 | Sun et al. |
| hsa-miR-618 | 0.869 | 1.639 | 7.462E-03 | Sun et al. |
| hsa-miR-143-3p | -0.933 | 17.773 | 8.061E-03 | Sun et al. |
| hsa-miR-106b-5p | 0.495 | 9.346 | 8.224E-03 | Sun et al. |
| hsa-miR-550a-3p | 0.508 | 1.300 | 8.224E-03 | Sun et al. |
| hsa-miR-5701 | 0.567 | 3.077 | 8.224E-03 | Sun et al. |
| hsa-miR-320c | 0.555 | 3.405 | 8.376E-03 | Sun et al. |
| hsa-miR-1301-3p | 0.371 | 3.504 | 8.643E-03 | Sun et al. |
| hsa-miR-222-3p | 0.327 | 8.662 | 9.120E-03 | Sun et al. |
| hsa-miR-34a-5p | 0.480 | 7.676 | 9.120E-03 | Sun et al. |
| hsa-miR-17-3p | 0.467 | 4.090 | 9.120E-03 | Sun et al. |
| hsa-miR-345-5p | 0.424 | 5.029 | 9.120E-03 | Sun et al. |
| hsa-miR-500a-5p | 0.398 | 4.124 | 9.235E-03 | Sun et al. |
| hsa-miR-146b-3p | 0.514 | 4.658 | 9.383E-03 | Sun et al. |
| hsa-miR-382-3p | 0.467 | 2.425 | 9.383E-03 | Sun et al. |
| hsa-miR-500b-5p | 0.394 | 4.122 | 9.814E-03 | Sun et al. |
| hsa-miR-154-3p | 0.486 | 3.435 | 1.075E-02 | Sun et al. |
| hsa-miR-665 | 0.591 | -0.118 | 1.163E-02 | Sun et al. |
| hsa-miR-142-3p | 0.839 | 9.621 | 1.178E-02 | Sun et al. |
| hsa-miR-486-5p | -0.718 | 6.790 | 1.178E-02 | Sun et al. |
| hsa-miR-106b-3p | 0.309 | 6.036 | 1.178E-02 | Sun et al. |
| hsa-miR-147b | -1.185 | 3.151 | 1.178E-02 | Sun et al. |
| hsa-miR-20a-3p | 0.574 | 3.638 | 1.188E-02 | Sun et al. |
| hsa-miR-181b-5p | 0.266 | 7.119 | 1.271E-02 | Sun et al. |
| hsa-miR-93-5p | 0.330 | 9.884 | 1.342E-02 | Sun et al. |
| hsa-miR-185-5p | 0.314 | 7.547 | 1.428E-02 | Sun et al. |
| hsa-miR-494-3p | 0.438 | 4.882 | 1.536E-02 | Sun et al. |
| hsa-miR-328-3p | -0.615 | 3.582 | 1.539E-02 | Sun et al. |
| hsa-miR-592 | 1.180 | 4.024 | 1.551E-02 | Sun et al. |
| hsa-miR-1306-5p | -0.400 | 1.858 | 1.625E-02 | Sun et al. |
| hsa-miR-374b-3p | 0.507 | 2.510 | 1.625E-02 | Sun et al. |
| hsa-miR-490-3p | -1.877 | 2.688 | 1.699E-02 | Sun et al. |
| hsa-miR-223-5p | 0.491 | 2.009 | 1.700E-02 | Sun et al. |
| hsa-miR-484 | -0.211 | 6.156 | 1.813E-02 | Sun et al. |
| hsa-miR-23b-3p | -0.478 | 11.457 | 1.813E-02 | Sun et al. |
| hsa-miR-552-5p | 1.108 | 4.904 | 1.837E-02 | Sun et al. |
| hsa-miR-1271-5p | -0.567 | 1.463 | 1.936E-02 | Sun et al. |
| hsa-miR-654-5p | 0.467 | 2.470 | 1.961E-02 | Sun et al. |
| hsa-miR-1296-5p | -0.361 | 4.205 | 1.986E-02 | Sun et al. |
| hsa-miR-381-3p | -0.402 | 5.310 | 1.996E-02 | Sun et al. |
| hsa-miR-181c-3p | 0.399 | 3.992 | 2.051E-02 | Sun et al. |
| hsa-miR-652-5p | 0.634 | 1.283 | 2.176E-02 | Sun et al. |
| hsa-miR-4443 | 0.713 | 0.929 | 2.189E-02 | Sun et al. |
| hsa-miR-99b-5p | -0.579 | 9.496 | 2.215E-02 | Sun et al. |
| hsa-miR-561-5p | -0.463 | 1.075 | 2.309E-02 | Sun et al. |
| hsa-miR-7705 | 0.763 | 0.780 | 2.334E-02 | Sun et al. |
| hsa-miR-625-5p | 0.498 | 3.055 | 2.399E-02 | Sun et al. |
| hsa-miR-143-5p | -0.735 | 11.217 | 2.564E-02 | Sun et al. |
| hsa-miR-221-5p | 0.296 | 5.469 | 2.589E-02 | Sun et al. |
| hsa-miR-552-3p | 1.075 | 5.697 | 2.592E-02 | Sun et al. |
| hsa-miR-151b | -0.349 | 5.421 | 2.635E-02 | Sun et al. |
| hsa-miR-483-3p | 0.941 | 0.987 | 2.643E-02 | Sun et al. |
| hsa-miR-642a-5p | -0.752 | 0.506 | 2.643E-02 | Sun et al. |
| hsa-miR-296-5p | -0.504 | 1.851 | 2.685E-02 | Sun et al. |
| hsa-miR-27b-3p | -0.353 | 12.590 | 2.728E-02 | Sun et al. |
| hsa-miR-146b-5p | 0.491 | 11.432 | 2.848E-02 | Sun et al. |
| hsa-miR-301a-3p | 0.505 | 2.402 | 2.859E-02 | Sun et al. |
| hsa-miR-34c-3p | 0.557 | 0.108 | 3.006E-02 | Sun et al. |
| hsa-miR-3648 | 0.885 | 0.930 | 3.067E-02 | Sun et al. |
| hsa-miR-550a-5p | 0.416 | 1.819 | 3.072E-02 | Sun et al. |
| hsa-miR-330-5p | 0.318 | 2.343 | 3.135E-02 | Sun et al. |
| hsa-let-7e-3p | -0.557 | 3.469 | 3.135E-02 | Sun et al. |
| hsa-miR-320b | 0.380 | 4.903 | 3.592E-02 | Sun et al. |
| hsa-miR-490-5p | -1.503 | 1.001 | 3.619E-02 | Sun et al. |
| hsa-miR-1246 | 1.266 | 3.197 | 3.685E-02 | Sun et al. |
| hsa-miR-16-2-3p | 0.276 | 4.037 | 3.832E-02 | Sun et al. |
| hsa-miR-192-3p | -0.883 | 3.420 | 3.931E-02 | Sun et al. |
| hsa-miR-1287-5p | -0.384 | 0.817 | 3.939E-02 | Sun et al. |
| hsa-miR-18b-5p | 0.432 | 2.055 | 4.109E-02 | Sun et al. |
| hsa-miR-130b-3p | 0.429 | 4.085 | 4.158E-02 | Sun et al. |
| hsa-miR-380-3p | 0.478 | 0.293 | 4.177E-02 | Sun et al. |
| hsa-let-7b-5p | -0.249 | 11.831 | 4.416E-02 | Sun et al. |
| hsa-miR-221-3p | 0.231 | 8.753 | 4.526E-02 | Sun et al. |
| hsa-miR-29a-5p | 0.353 | 3.123 | 4.786E-02 | Sun et al. |
| hsa-miR-7641 | 0.661 | 2.563 | 4.845E-02 | Sun et al. |
| hsa-miR-382-5p | 0.346 | 4.016 | 4.845E-02 | Sun et al. |
| hsa-miR-455-3p | 0.347 | 5.178 | 4.910E-02 | Sun et al. |
| hsa-miR-20b-5p | -0.827 | 4.297 | 4.910E-02 | Sun et al. |
| hsa-miR-409-5p | 0.327 | 3.588 | 5.039E-02 | Sun et al. |
| hsa-let-7d-3p | -0.271 | 6.222 | 5.039E-02 | Sun et al. |
| hsa-miR-423-5p | 0.317 | 8.412 | 5.179E-02 | Sun et al. |
| hsa-miR-2355-3p | 0.366 | 1.623 | 5.179E-02 | Sun et al. |
| hsa-miR-1277-5p | 0.660 | -0.158 | 5.227E-02 | Sun et al. |
| hsa-miR-339-5p | 0.279 | 7.039 | 5.227E-02 | Sun et al. |
| hsa-miR-19a-3p | 0.659 | 9.237 | 5.268E-02 | Sun et al. |
| hsa-miR-6511b-3p | -0.577 | 0.587 | 5.536E-02 | Sun et al. |
| hsa-miR-144-5p | -0.573 | 5.638 | 5.731E-02 | Sun et al. |
| hsa-miR-125a-5p | -0.476 | 11.101 | 5.747E-02 | Sun et al. |
| hsa-miR-548ba | -1.259 | 0.702 | 5.978E-02 | Sun et al. |
| hsa-miR-671-5p | 0.413 | 3.248 | 6.107E-02 | Sun et al. |
| hsa-miR-194-3p | -0.764 | 3.792 | 6.107E-02 | Sun et al. |
| hsa-let-7g-3p | -0.333 | 0.396 | 6.146E-02 | Sun et al. |
| hsa-miR-24-1-5p | -0.368 | 4.697 | 6.344E-02 | Sun et al. |
| hsa-miR-548o-3p | 0.369 | 0.895 | 6.478E-02 | Sun et al. |
| hsa-miR-532-5p | 0.258 | 7.076 | 6.480E-02 | Sun et al. |
| hsa-miR-203a-3p | 0.780 | 9.789 | 6.597E-02 | Sun et al. |
| hsa-miR-218-1-3p | -0.600 | 0.969 | 6.760E-02 | Sun et al. |
| hsa-miR-656-3p | 0.313 | 2.271 | 6.922E-02 | Sun et al. |
| hsa-miR-30d-3p | -0.449 | 0.647 | 6.976E-02 | Sun et al. |
| hsa-miR-27b-5p | -0.298 | 6.919 | 7.015E-02 | Sun et al. |
| hsa-miR-4326 | 0.667 | 0.953 | 7.081E-02 | Sun et al. |
| hsa-miR-130b-5p | 0.328 | 3.371 | 7.265E-02 | Sun et al. |
| hsa-miR-197-3p | -0.217 | 7.567 | 7.536E-02 | Sun et al. |
| hsa-miR-889-3p | 0.295 | 4.838 | 7.735E-02 | Sun et al. |
| hsa-miR-874-3p | 0.234 | 3.805 | 7.886E-02 | Sun et al. |
| hsa-miR-363-3p | -0.847 | 5.867 | 7.886E-02 | Sun et al. |
| hsa-miR-369-3p | 0.351 | 4.639 | 7.894E-02 | Sun et al. |
| hsa-miR-941 | 0.336 | 3.841 | 8.279E-02 | Sun et al. |
| hsa-miR-19b-1-5p | 0.474 | 0.242 | 8.291E-02 | Sun et al. |
| hsa-miR-3913-5p | 0.255 | 1.343 | 8.616E-02 | Sun et al. |
| hsa-miR-376b-3p | 0.484 | 0.941 | 8.696E-02 | Sun et al. |
| hsa-miR-375 | -0.848 | 8.932 | 8.715E-02 | Sun et al. |
| hsa-miR-148b-5p | 0.250 | 3.842 | 8.825E-02 | Sun et al. |
| hsa-miR-362-5p | 0.250 | 6.020 | 8.830E-02 | Sun et al. |
| hsa-let-7i-5p | 0.240 | 11.834 | 8.947E-02 | Sun et al. |
| hsa-miR-874-5p | 0.381 | 0.703 | 8.947E-02 | Sun et al. |
| hsa-miR-224-3p | 0.362 | 3.836 | 8.974E-02 | Sun et al. |
| hsa-miR-214-3p | 0.366 | 7.489 | 9.129E-02 | Sun et al. |
| hsa-miR-500a-3p | 0.201 | 5.992 | 9.416E-02 | Sun et al. |
| hsa-miR-203a-5p | 0.669 | 0.383 | 9.539E-02 | Sun et al. |
| hsa-miR-1248 | 0.411 | 4.722 | 9.539E-02 | Sun et al. |
| hsa-miR-1297 | -0.388 | 1.939 | 9.864E-02 | Sun et al. |
| hsa-miR-411-5p | 0.298 | 6.514 | 1.010E-01 | Sun et al. |
| hsa-miR-33a-3p | -0.294 | 1.425 | 1.219E-01 | Sun et al. |
| hsa-miR-19b-3p | 0.377 | 10.561 | 1.224E-01 | Sun et al. |
| hsa-miR-100-5p | -0.425 | 11.122 | 1.270E-01 | Sun et al. |
| hsa-miR-3065-3p | -0.352 | 0.849 | 1.296E-01 | Sun et al. |
| hsa-miR-365a-3p | -0.427 | 6.512 | 1.324E-01 | Sun et al. |
| hsa-miR-365b-3p | -0.426 | 6.513 | 1.324E-01 | Sun et al. |
| hsa-miR-769-5p | 0.141 | 7.312 | 1.354E-01 | Sun et al. |
| hsa-miR-543 | 0.291 | 1.687 | 1.376E-01 | Sun et al. |
| hsa-miR-548b-3p | -0.488 | 0.796 | 1.380E-01 | Sun et al. |
| hsa-miR-365a-5p | 0.405 | 0.731 | 1.452E-01 | Sun et al. |
| hsa-let-7a-3p | 0.198 | 4.287 | 1.478E-01 | Sun et al. |
| hsa-miR-3609 | 0.391 | 2.050 | 1.479E-01 | Sun et al. |
| hsa-miR-4286 | 0.448 | 1.320 | 1.501E-01 | Sun et al. |
| hsa-miR-455-5p | 0.275 | 7.882 | 1.501E-01 | Sun et al. |
| hsa-miR-655-3p | 0.251 | 3.180 | 1.506E-01 | Sun et al. |
| hsa-miR-580-3p | 0.414 | 0.390 | 1.524E-01 | Sun et al. |
| hsa-miR-126-3p | -0.221 | 10.409 | 1.524E-01 | Sun et al. |
| hsa-miR-140-5p | -0.212 | 8.064 | 1.524E-01 | Sun et al. |
| hsa-miR-501-3p | 0.212 | 4.248 | 1.524E-01 | Sun et al. |
| hsa-miR-3615 | 0.335 | 0.799 | 1.524E-01 | Sun et al. |
| hsa-miR-3656 | 0.583 | 1.103 | 1.524E-01 | Sun et al. |
| hsa-miR-127-5p | 0.259 | 5.084 | 1.573E-01 | Sun et al. |
| hsa-miR-34c-5p | 0.363 | 4.420 | 1.642E-01 | Sun et al. |
| hsa-miR-374c-5p | 0.240 | 3.155 | 1.676E-01 | Sun et al. |
| hsa-miR-146a-5p | 0.435 | 11.423 | 1.687E-01 | Sun et al. |
| hsa-miR-3651 | 0.486 | 0.128 | 1.732E-01 | Sun et al. |
| hsa-miR-539-3p | 0.345 | 1.931 | 1.928E-01 | Sun et al. |
| hsa-miR-33a-5p | 0.548 | 3.196 | 1.951E-01 | Sun et al. |
| hsa-miR-625-3p | 0.310 | 5.163 | 1.997E-01 | Sun et al. |
| hsa-miR-23b-5p | -0.229 | 1.620 | 2.022E-01 | Sun et al. |
| hsa-miR-202-5p | -0.440 | 0.248 | 2.022E-01 | Sun et al. |
| hsa-miR-1185-5p | 0.273 | 1.082 | 2.034E-01 | Sun et al. |
| hsa-miR-126-5p | -0.243 | 7.711 | 2.034E-01 | Sun et al. |
| hsa-miR-193b-3p | -0.251 | 6.497 | 2.053E-01 | Sun et al. |
| hsa-miR-652-3p | 0.193 | 6.925 | 2.055E-01 | Sun et al. |
| hsa-let-7a-5p | -0.155 | 12.198 | 2.106E-01 | Sun et al. |
| hsa-miR-26a-2-3p | -0.225 | 2.373 | 2.145E-01 | Sun et al. |
| hsa-miR-32-3p | 0.383 | 0.895 | 2.154E-01 | Sun et al. |
| hsa-miR-411-3p | 0.262 | 1.882 | 2.154E-01 | Sun et al. |
| hsa-miR-664a-3p | -0.217 | 6.153 | 2.154E-01 | Sun et al. |
| hsa-miR-194-5p | -0.555 | 13.925 | 2.154E-01 | Sun et al. |
| hsa-miR-331-3p | 0.169 | 4.683 | 2.154E-01 | Sun et al. |
| hsa-miR-654-3p | 0.234 | 3.852 | 2.160E-01 | Sun et al. |
| hsa-miR-3200-3p | 0.342 | 0.186 | 2.171E-01 | Sun et al. |
| hsa-miR-590-3p | 0.358 | 4.193 | 2.214E-01 | Sun et al. |
| hsa-miR-192-5p | -0.550 | 13.748 | 2.236E-01 | Sun et al. |
| hsa-miR-502-5p | 0.276 | 1.528 | 2.252E-01 | Sun et al. |
| hsa-miR-3613-5p | 0.346 | 6.746 | 2.273E-01 | Sun et al. |
| hsa-miR-99a-5p | -0.452 | 10.066 | 2.343E-01 | Sun et al. |
| hsa-miR-576-5p | -0.156 | 2.866 | 2.348E-01 | Sun et al. |
| hsa-miR-651-5p | 0.345 | 4.287 | 2.361E-01 | Sun et al. |
| hsa-miR-376a-3p | 0.253 | 2.281 | 2.482E-01 | Sun et al. |
| hsa-miR-425-5p | 0.221 | 10.910 | 2.528E-01 | Sun et al. |
| hsa-miR-214-5p | 0.226 | 4.701 | 2.528E-01 | Sun et al. |
| hsa-miR-2355-5p | 0.266 | 0.482 | 2.528E-01 | Sun et al. |
| hsa-miR-585-3p | -0.417 | 1.114 | 2.528E-01 | Sun et al. |
| hsa-miR-6516-3p | 0.333 | 1.980 | 2.586E-01 | Sun et al. |
| hsa-miR-362-3p | 0.294 | 2.809 | 2.612E-01 | Sun et al. |
| hsa-miR-323a-3p | -0.276 | 2.054 | 2.637E-01 | Sun et al. |
| hsa-miR-32-5p | 0.371 | 4.715 | 2.657E-01 | Sun et al. |
| hsa-miR-185-3p | 0.191 | 0.331 | 2.708E-01 | Sun et al. |
| hsa-miR-99a-3p | -0.397 | 1.777 | 2.732E-01 | Sun et al. |
| hsa-miR-340-5p | -0.115 | 8.572 | 2.814E-01 | Sun et al. |
| hsa-miR-628-5p | -0.178 | 2.352 | 2.847E-01 | Sun et al. |
| hsa-miR-582-5p | -0.228 | 5.002 | 2.922E-01 | Sun et al. |
| hsa-miR-92b-3p | 0.195 | 6.370 | 2.943E-01 | Sun et al. |
| hsa-miR-144-3p | -0.427 | 6.086 | 2.943E-01 | Sun et al. |
| hsa-miR-193a-3p | -0.228 | 4.168 | 2.949E-01 | Sun et al. |
| hsa-miR-432-5p | 0.199 | 2.584 | 3.014E-01 | Sun et al. |
| hsa-miR-142-5p | 0.267 | 8.651 | 3.073E-01 | Sun et al. |
| hsa-miR-412-5p | 0.391 | 0.279 | 3.134E-01 | Sun et al. |
| hsa-miR-361-5p | -0.126 | 8.064 | 3.134E-01 | Sun et al. |
| hsa-miR-548n | 0.315 | 0.360 | 3.244E-01 | Sun et al. |
| hsa-miR-629-5p | 0.177 | 4.247 | 3.305E-01 | Sun et al. |
| hsa-miR-744-3p | 0.204 | 0.914 | 3.305E-01 | Sun et al. |
| hsa-miR-29b-3p | -0.194 | 7.639 | 3.460E-01 | Sun et al. |
| hsa-miR-93-3p | 0.121 | 3.832 | 3.460E-01 | Sun et al. |
| hsa-miR-148a-5p | 0.257 | 6.278 | 3.477E-01 | Sun et al. |
| hsa-let-7f-5p | -0.138 | 12.850 | 3.477E-01 | Sun et al. |
| hsa-miR-6511a-3p | -0.284 | 0.558 | 3.547E-01 | Sun et al. |
| hsa-miR-330-3p | -0.185 | 2.697 | 3.579E-01 | Sun et al. |
| hsa-miR-4521 | -0.371 | 0.877 | 3.609E-01 | Sun et al. |
| hsa-miR-454-5p | 0.197 | 0.277 | 3.609E-01 | Sun et al. |
| hsa-miR-101-3p | -0.205 | 12.950 | 3.609E-01 | Sun et al. |
| hsa-miR-374b-5p | -0.117 | 8.120 | 3.609E-01 | Sun et al. |
| hsa-miR-125a-3p | -0.215 | 1.469 | 3.617E-01 | Sun et al. |
| hsa-miR-4485-3p | 0.357 | 2.209 | 3.617E-01 | Sun et al. |
| hsa-miR-193a-5p | -0.265 | 4.444 | 3.617E-01 | Sun et al. |
| hsa-miR-196b-5p | 0.246 | 8.823 | 3.637E-01 | Sun et al. |
| hsa-miR-22-3p | -0.125 | 11.712 | 3.733E-01 | Sun et al. |
| hsa-miR-511-5p | -0.205 | 1.551 | 3.843E-01 | Sun et al. |
| hsa-miR-499a-5p | 0.271 | 3.014 | 3.843E-01 | Sun et al. |
| hsa-let-7d-5p | -0.114 | 8.818 | 3.945E-01 | Sun et al. |
| hsa-miR-532-3p | 0.144 | 4.205 | 3.945E-01 | Sun et al. |
| hsa-miR-181a-2-3p | -0.148 | 5.084 | 3.996E-01 | Sun et al. |
| hsa-miR-324-3p | -0.118 | 1.813 | 4.032E-01 | Sun et al. |
| hsa-miR-4485-5p | 0.319 | 1.495 | 4.034E-01 | Sun et al. |
| hsa-miR-377-5p | 0.216 | 1.234 | 4.035E-01 | Sun et al. |
| hsa-miR-155-5p | 0.217 | 7.704 | 4.126E-01 | Sun et al. |
| hsa-miR-582-3p | -0.191 | 4.491 | 4.136E-01 | Sun et al. |
| hsa-miR-3617-5p | 0.343 | 0.097 | 4.163E-01 | Sun et al. |
| hsa-let-7e-5p | -0.203 | 8.398 | 4.163E-01 | Sun et al. |
| hsa-miR-154-5p | -0.143 | 2.880 | 4.236E-01 | Sun et al. |
| hsa-miR-379-3p | 0.204 | 2.291 | 4.247E-01 | Sun et al. |
| hsa-miR-627-5p | -0.198 | 1.673 | 4.275E-01 | Sun et al. |
| hsa-miR-203b-3p | 0.374 | 3.228 | 4.294E-01 | Sun et al. |
| hsa-miR-3653-3p | -0.194 | 3.927 | 4.296E-01 | Sun et al. |
| hsa-miR-3909 | 0.191 | 0.993 | 4.340E-01 | Sun et al. |
| hsa-miR-369-5p | 0.169 | 2.505 | 4.340E-01 | Sun et al. |
| hsa-miR-23a-3p | 0.116 | 12.427 | 4.347E-01 | Sun et al. |
| hsa-let-7c-5p | -0.301 | 8.919 | 4.354E-01 | Sun et al. |
| hsa-miR-629-3p | -0.170 | 0.404 | 4.354E-01 | Sun et al. |
| hsa-miR-454-3p | 0.126 | 5.825 | 4.354E-01 | Sun et al. |
| hsa-miR-766-3p | -0.207 | 2.779 | 4.375E-01 | Sun et al. |
| hsa-miR-548i | -0.450 | 0.512 | 4.385E-01 | Sun et al. |
| hsa-let-7f-2-3p | 0.217 | -0.220 | 4.385E-01 | Sun et al. |
| hsa-miR-361-3p | -0.094 | 7.059 | 4.516E-01 | Sun et al. |
| hsa-miR-25-3p | 0.100 | 8.969 | 4.552E-01 | Sun et al. |
| hsa-miR-429 | 0.377 | 9.251 | 4.552E-01 | Sun et al. |
| hsa-miR-664a-5p | -0.144 | 1.824 | 4.552E-01 | Sun et al. |
| hsa-let-7b-3p | -0.121 | 3.099 | 4.552E-01 | Sun et al. |
| hsa-miR-130a-5p | -0.203 | 0.153 | 4.564E-01 | Sun et al. |
| hsa-miR-1307-3p | 0.127 | 6.685 | 4.571E-01 | Sun et al. |
| hsa-miR-103a-2-5p | 0.107 | 1.478 | 4.576E-01 | Sun et al. |
| hsa-miR-210-5p | -0.243 | -0.179 | 4.576E-01 | Sun et al. |
| hsa-miR-769-3p | 0.155 | 1.117 | 4.576E-01 | Sun et al. |
| hsa-miR-7-1-3p | -0.129 | 3.517 | 4.622E-01 | Sun et al. |
| hsa-miR-132-5p | -0.119 | 3.514 | 4.622E-01 | Sun et al. |
| hsa-miR-99b-3p | -0.172 | 3.667 | 4.622E-01 | Sun et al. |
| hsa-miR-127-3p | 0.142 | 6.894 | 4.674E-01 | Sun et al. |
| hsa-miR-199b-3p | 0.136 | 12.245 | 4.674E-01 | Sun et al. |
| hsa-miR-199a-3p | 0.136 | 12.246 | 4.674E-01 | Sun et al. |
| hsa-miR-3182 | 0.283 | 4.332 | 4.674E-01 | Sun et al. |
| hsa-miR-148b-3p | 0.118 | 10.719 | 4.674E-01 | Sun et al. |
| hsa-miR-485-3p | 0.161 | 1.330 | 4.674E-01 | Sun et al. |
| hsa-miR-660-5p | 0.153 | 6.681 | 4.674E-01 | Sun et al. |
| hsa-miR-107 | -0.076 | 6.749 | 4.689E-01 | Sun et al. |
| hsa-miR-22-5p | 0.118 | 4.630 | 4.690E-01 | Sun et al. |
| hsa-miR-337-5p | 0.148 | 2.419 | 4.690E-01 | Sun et al. |
| hsa-miR-1306-3p | 0.148 | 0.175 | 4.721E-01 | Sun et al. |
| hsa-miR-15a-5p | 0.145 | 8.074 | 4.805E-01 | Sun et al. |
| hsa-miR-136-3p | -0.181 | 4.945 | 4.852E-01 | Sun et al. |
| hsa-miR-1307-5p | 0.213 | 2.051 | 4.858E-01 | Sun et al. |
| hsa-miR-590-5p | 0.215 | 1.401 | 5.011E-01 | Sun et al. |
| hsa-miR-1180-3p | 0.167 | 1.642 | 5.013E-01 | Sun et al. |
| hsa-miR-4516 | -0.315 | 1.288 | 5.035E-01 | Sun et al. |
| hsa-miR-24-2-5p | 0.137 | 6.646 | 5.035E-01 | Sun et al. |
| hsa-miR-152-3p | -0.119 | 9.874 | 5.035E-01 | Sun et al. |
| hsa-miR-671-3p | 0.150 | 1.009 | 5.035E-01 | Sun et al. |
| hsa-let-7a-2-3p | 0.211 | 0.428 | 5.144E-01 | Sun et al. |
| hsa-miR-505-3p | -0.070 | 5.991 | 5.163E-01 | Sun et al. |
| hsa-miR-511-3p | -0.179 | 2.245 | 5.229E-01 | Sun et al. |
| hsa-miR-151a-3p | 0.069 | 9.963 | 5.365E-01 | Sun et al. |
| hsa-miR-10a-5p | -0.104 | 13.358 | 5.367E-01 | Sun et al. |
| hsa-miR-660-3p | 0.136 | 0.955 | 5.375E-01 | Sun et al. |
| hsa-miR-574-5p | -0.281 | 3.700 | 5.376E-01 | Sun et al. |
| hsa-miR-301a-5p | 0.149 | 0.448 | 5.391E-01 | Sun et al. |
| hsa-miR-335-5p | 0.136 | 9.102 | 5.496E-01 | Sun et al. |
| hsa-miR-128-3p | 0.065 | 6.340 | 5.661E-01 | Sun et al. |
| hsa-miR-505-5p | 0.093 | 2.031 | 5.661E-01 | Sun et al. |
| hsa-miR-15b-3p | 0.087 | 3.427 | 5.661E-01 | Sun et al. |
| hsa-miR-1260b | 0.173 | 2.840 | 5.661E-01 | Sun et al. |
| hsa-miR-487b-3p | -0.095 | 5.443 | 5.664E-01 | Sun et al. |
| hsa-miR-212-3p | 0.096 | 2.807 | 5.704E-01 | Sun et al. |
| hsa-miR-29a-3p | 0.066 | 9.938 | 5.710E-01 | Sun et al. |
| hsa-miR-3065-5p | -0.136 | 2.212 | 5.728E-01 | Sun et al. |
| hsa-miR-7706 | 0.138 | 0.078 | 5.728E-01 | Sun et al. |
| hsa-miR-148a-3p | 0.168 | 15.057 | 5.798E-01 | Sun et al. |
| hsa-miR-548k | 0.119 | 1.209 | 5.798E-01 | Sun et al. |
| hsa-miR-379-5p | 0.101 | 7.014 | 5.798E-01 | Sun et al. |
| hsa-miR-16-5p | 0.071 | 10.943 | 5.859E-01 | Sun et al. |
| hsa-miR-151a-5p | -0.085 | 9.680 | 5.863E-01 | Sun et al. |
| hsa-miR-98-5p | 0.102 | 7.901 | 5.921E-01 | Sun et al. |
| hsa-miR-576-3p | -0.107 | 3.262 | 5.923E-01 | Sun et al. |
| hsa-miR-377-3p | 0.128 | 2.614 | 5.959E-01 | Sun et al. |
| hsa-miR-433-3p | 0.129 | 2.561 | 6.024E-01 | Sun et al. |
| hsa-miR-24-3p | 0.057 | 10.855 | 6.116E-01 | Sun et al. |
| hsa-miR-3653-5p | -0.129 | 2.983 | 6.116E-01 | Sun et al. |
| hsa-miR-329-3p | -0.101 | 2.552 | 6.190E-01 | Sun et al. |
| hsa-miR-539-5p | -0.108 | 0.831 | 6.218E-01 | Sun et al. |
| hsa-miR-3912-3p | 0.117 | 0.788 | 6.234E-01 | Sun et al. |
| hsa-miR-6087 | 0.187 | 5.635 | 6.364E-01 | Sun et al. |
| hsa-miR-125b-2-3p | -0.194 | 3.941 | 6.384E-01 | Sun et al. |
| hsa-let-7i-3p | 0.071 | 4.025 | 6.430E-01 | Sun et al. |
| hsa-miR-15b-5p | -0.075 | 8.312 | 6.485E-01 | Sun et al. |
| hsa-miR-181a-5p | 0.059 | 10.287 | 6.503E-01 | Sun et al. |
| hsa-miR-556-5p | -0.125 | 2.725 | 6.516E-01 | Sun et al. |
| hsa-miR-299-5p | -0.094 | 2.314 | 6.518E-01 | Sun et al. |
| hsa-miR-664b-3p | 0.081 | 3.077 | 6.532E-01 | Sun et al. |
| hsa-miR-200a-3p | 0.209 | 11.666 | 6.669E-01 | Sun et al. |
| hsa-miR-150-5p | -0.111 | 8.220 | 6.682E-01 | Sun et al. |
| hsa-miR-495-3p | 0.057 | 5.611 | 6.759E-01 | Sun et al. |
| hsa-miR-7704 | 0.122 | 1.085 | 6.800E-01 | Sun et al. |
| hsa-miR-653-5p | 0.123 | 0.907 | 6.800E-01 | Sun et al. |
| hsa-miR-337-3p | -0.076 | 3.619 | 6.953E-01 | Sun et al. |
| hsa-miR-210-3p | 0.137 | 5.201 | 6.967E-01 | Sun et al. |
| hsa-miR-125b-1-3p | 0.133 | 2.344 | 7.059E-01 | Sun et al. |
| hsa-miR-376c-3p | -0.086 | 5.048 | 7.103E-01 | Sun et al. |
| hsa-miR-3613-3p | 0.074 | 1.640 | 7.142E-01 | Sun et al. |
| hsa-miR-374a-3p | 0.097 | 6.296 | 7.303E-01 | Sun et al. |
| hsa-miR-196b-3p | 0.133 | 0.066 | 7.355E-01 | Sun et al. |
| hsa-miR-485-5p | -0.079 | 1.036 | 7.367E-01 | Sun et al. |
| hsa-miR-141-5p | 0.164 | 1.846 | 7.373E-01 | Sun et al. |
| hsa-miR-320a | -0.048 | 9.147 | 7.373E-01 | Sun et al. |
| hsa-miR-410-3p | 0.071 | 3.707 | 7.373E-01 | Sun et al. |
| hsa-miR-342-3p | -0.062 | 7.637 | 7.373E-01 | Sun et al. |
| hsa-miR-199b-5p | 0.061 | 9.812 | 7.428E-01 | Sun et al. |
| hsa-miR-491-5p | -0.053 | 1.668 | 7.527E-01 | Sun et al. |
| hsa-miR-942-5p | 0.085 | 1.643 | 7.527E-01 | Sun et al. |
| hsa-miR-1277-3p | 0.118 | -0.013 | 7.540E-01 | Sun et al. |
| hsa-miR-125b-5p | -0.110 | 11.357 | 7.717E-01 | Sun et al. |
| hsa-miR-199a-5p | 0.061 | 10.712 | 7.720E-01 | Sun et al. |
| hsa-miR-3607-5p | 0.076 | 2.813 | 7.755E-01 | Sun et al. |
| hsa-miR-4791 | 0.107 | 2.484 | 7.778E-01 | Sun et al. |
| hsa-miR-141-3p | 0.154 | 8.484 | 7.812E-01 | Sun et al. |
| hsa-miR-212-5p | 0.068 | 0.489 | 7.904E-01 | Sun et al. |
| hsa-miR-340-3p | -0.058 | 3.927 | 8.039E-01 | Sun et al. |
| hsa-miR-653-3p | 0.091 | 0.230 | 8.103E-01 | Sun et al. |
| hsa-miR-376b-5p | 0.083 | 0.336 | 8.106E-01 | Sun et al. |
| hsa-miR-1291 | 0.071 | 2.727 | 8.117E-01 | Sun et al. |
| hsa-miR-103a-3p | 0.023 | 11.947 | 8.117E-01 | Sun et al. |
| hsa-miR-339-3p | 0.033 | 5.629 | 8.117E-01 | Sun et al. |
| hsa-miR-132-3p | -0.041 | 6.575 | 8.117E-01 | Sun et al. |
| hsa-miR-4636 | -0.085 | 0.686 | 8.164E-01 | Sun et al. |
| hsa-miR-299-3p | 0.060 | 0.900 | 8.164E-01 | Sun et al. |
| hsa-miR-3158-3p | 0.051 | 1.696 | 8.184E-01 | Sun et al. |
| hsa-miR-502-3p | 0.026 | 5.646 | 8.184E-01 | Sun et al. |
| hsa-miR-641 | -0.053 | 0.923 | 8.232E-01 | Sun et al. |
| hsa-let-7f-1-3p | 0.049 | 1.166 | 8.234E-01 | Sun et al. |
| hsa-miR-577 | 0.111 | 4.181 | 8.251E-01 | Sun et al. |
| hsa-miR-331-5p | 0.036 | 2.627 | 8.287E-01 | Sun et al. |
| hsa-miR-200b-3p | 0.092 | 13.782 | 8.302E-01 | Sun et al. |
| hsa-miR-589-5p | -0.030 | 3.315 | 8.317E-01 | Sun et al. |
| hsa-miR-548e-3p | -0.042 | 3.553 | 8.317E-01 | Sun et al. |
| hsa-miR-324-5p | -0.030 | 6.283 | 8.317E-01 | Sun et al. |
| hsa-miR-153-3p | -0.086 | 1.110 | 8.338E-01 | Sun et al. |
| hsa-miR-376a-5p | -0.039 | 2.609 | 8.367E-01 | Sun et al. |
| hsa-miR-196a-5p | -0.087 | 6.766 | 8.371E-01 | Sun et al. |
| hsa-miR-342-5p | -0.036 | 3.622 | 8.403E-01 | Sun et al. |
| hsa-miR-10a-3p | -0.028 | 5.455 | 8.436E-01 | Sun et al. |
| hsa-miR-320e | 0.056 | 0.272 | 8.518E-01 | Sun et al. |
| hsa-miR-33b-5p | 0.057 | 0.241 | 8.772E-01 | Sun et al. |
| hsa-miR-4497 | 0.049 | 0.417 | 8.882E-01 | Sun et al. |
| hsa-miR-200c-5p | -0.053 | 0.573 | 8.925E-01 | Sun et al. |
| hsa-miR-200a-5p | 0.056 | 5.663 | 9.028E-01 | Sun et al. |
| hsa-miR-3607-3p | -0.025 | 6.947 | 9.028E-01 | Sun et al. |
| hsa-miR-2110 | 0.018 | 2.081 | 9.057E-01 | Sun et al. |
| hsa-miR-5571-3p | 0.043 | 1.521 | 9.242E-01 | Sun et al. |
| hsa-miR-16-1-3p | -0.036 | 0.797 | 9.257E-01 | Sun et al. |
| hsa-miR-136-5p | -0.021 | 4.071 | 9.394E-01 | Sun et al. |
| hsa-miR-193b-5p | -0.020 | 0.893 | 9.426E-01 | Sun et al. |
| hsa-miR-181c-5p | 0.009 | 5.628 | 9.501E-01 | Sun et al. |
| hsa-miR-376c-5p | 0.021 | 0.387 | 9.513E-01 | Sun et al. |
| hsa-miR-200c-3p | -0.020 | 13.230 | 9.652E-01 | Sun et al. |
| hsa-miR-374a-5p | 0.007 | 8.932 | 9.717E-01 | Sun et al. |
| hsa-miR-200b-5p | 0.014 | 6.079 | 9.762E-01 | Sun et al. |
| hsa-miR-191-5p | 0.003 | 10.305 | 9.851E-01 | Sun et al. |
| hsa-miR-423-3p | -0.003 | 9.317 | 9.851E-01 | Sun et al. |
| hsa-miR-744-5p | -0.003 | 5.237 | 9.854E-01 | Sun et al. |
| hsa-miR-425-3p | -0.001 | 4.932 | 9.943E-01 | Sun et al. |
| hsa-miR-1249-3p | -0.001 | 2.113 | 9.968E-01 | Sun et al. |
| hsa-miR-135b-3p | 3.994 | -0.174 | 1.330E-18 | Neerincx et al. |
| hsa-miR-135b-5p | 4.182 | 3.901 | 3.741E-17 | Neerincx et al. |
| hsa-miR-215-5p | -4.586 | 11.291 | 2.333E-14 | Neerincx et al. |
| hsa-miR-224-5p | 2.379 | 6.886 | 7.199E-14 | Neerincx et al. |
| hsa-miR-378a-3p | -2.128 | 11.980 | 1.151E-13 | Neerincx et al. |
| hsa-miR-378c | -2.041 | 7.618 | 1.559E-13 | Neerincx et al. |
| hsa-miR-139-5p | -2.264 | 3.497 | 7.644E-13 | Neerincx et al. |
| hsa-miR-378d | -2.076 | 5.364 | 1.245E-12 | Neerincx et al. |
| hsa-miR-215-3p | -4.379 | 2.169 | 3.041E-11 | Neerincx et al. |
| hsa-miR-378a-5p | -2.385 | 4.974 | 8.808E-11 | Neerincx et al. |
| hsa-miR-147b | -2.857 | 4.863 | 3.335E-10 | Neerincx et al. |
| hsa-miR-190a-5p | -2.194 | 6.858 | 1.090E-09 | Neerincx et al. |
| hsa-miR-30e-5p | -1.290 | 11.824 | 2.108E-09 | Neerincx et al. |
| hsa-miR-29c-5p | -1.391 | 3.241 | 2.108E-09 | Neerincx et al. |
| hsa-miR-30c-5p | -1.304 | 9.594 | 2.551E-09 | Neerincx et al. |
| hsa-miR-92a-3p | 1.346 | 13.538 | 3.776E-09 | Neerincx et al. |
| hsa-miR-195-3p | -1.457 | 3.275 | 4.171E-09 | Neerincx et al. |
| hsa-miR-195-5p | -1.746 | 8.268 | 8.536E-09 | Neerincx et al. |
| hsa-miR-29c-3p | -1.771 | 7.748 | 8.536E-09 | Neerincx et al. |
| hsa-miR-431-5p | 1.637 | 2.411 | 1.193E-08 | Neerincx et al. |
| hsa-miR-183-3p | 2.285 | 1.422 | 1.291E-08 | Neerincx et al. |
| hsa-miR-497-5p | -1.676 | 7.393 | 2.121E-08 | Neerincx et al. |
| hsa-miR-181d-5p | 1.386 | 6.174 | 2.121E-08 | Neerincx et al. |
| hsa-miR-493-3p | 1.459 | 2.482 | 2.370E-08 | Neerincx et al. |
| hsa-miR-31-5p | 3.754 | 4.715 | 2.370E-08 | Neerincx et al. |
| hsa-miR-584-5p | 2.179 | 4.645 | 2.370E-08 | Neerincx et al. |
| hsa-miR-26b-5p | -1.029 | 11.544 | 5.170E-08 | Neerincx et al. |
| hsa-miR-9-5p | -2.397 | 4.496 | 5.838E-08 | Neerincx et al. |
| hsa-miR-190a-3p | -1.908 | 0.814 | 1.105E-07 | Neerincx et al. |
| hsa-miR-30a-5p | -1.460 | 11.466 | 1.183E-07 | Neerincx et al. |
| hsa-miR-28-5p | -0.955 | 8.660 | 1.275E-07 | Neerincx et al. |
| hsa-miR-335-3p | 1.451 | 8.102 | 1.602E-07 | Neerincx et al. |
| hsa-miR-21-3p | 1.564 | 11.253 | 1.998E-07 | Neerincx et al. |
| hsa-miR-30e-3p | -1.243 | 7.503 | 2.466E-07 | Neerincx et al. |
| hsa-miR-7974 | 2.996 | 2.514 | 2.537E-07 | Neerincx et al. |
| hsa-miR-92a-1-5p | 1.613 | 2.900 | 3.481E-07 | Neerincx et al. |
| hsa-miR-133a-3p | -2.511 | 6.398 | 3.854E-07 | Neerincx et al. |
| hsa-miR-183-5p | 2.281 | 9.072 | 3.854E-07 | Neerincx et al. |
| hsa-miR-18a-3p | 1.289 | 2.367 | 8.414E-07 | Neerincx et al. |
| hsa-miR-30a-3p | -1.325 | 5.553 | 1.250E-06 | Neerincx et al. |
| hsa-miR-182-5p | 1.713 | 12.157 | 1.601E-06 | Neerincx et al. |
| hsa-miR-17-5p | 1.128 | 8.686 | 1.739E-06 | Neerincx et al. |
| hsa-miR-375 | -2.136 | 12.469 | 3.225E-06 | Neerincx et al. |
| hsa-miR-424-3p | 1.492 | 3.722 | 3.232E-06 | Neerincx et al. |
| hsa-miR-194-5p | -1.649 | 12.724 | 3.232E-06 | Neerincx et al. |
| hsa-miR-26a-5p | -1.107 | 15.064 | 3.413E-06 | Neerincx et al. |
| hsa-miR-7641 | 3.073 | 2.693 | 3.702E-06 | Neerincx et al. |
| hsa-miR-511-5p | -1.339 | 1.373 | 3.922E-06 | Neerincx et al. |
| hsa-miR-452-5p | 1.120 | 5.257 | 5.504E-06 | Neerincx et al. |
| hsa-miR-133b | -2.439 | 1.018 | 6.185E-06 | Neerincx et al. |
| hsa-miR-96-5p | 1.878 | 4.621 | 7.988E-06 | Neerincx et al. |
| hsa-miR-30c-1-3p | -1.206 | 3.058 | 9.995E-06 | Neerincx et al. |
| hsa-miR-138-5p | -2.191 | 2.793 | 1.132E-05 | Neerincx et al. |
| hsa-miR-708-5p | 1.910 | 3.764 | 1.855E-05 | Neerincx et al. |
| hsa-miR-181c-3p | 1.105 | 5.241 | 1.956E-05 | Neerincx et al. |
| hsa-miR-708-3p | 1.917 | 3.194 | 2.098E-05 | Neerincx et al. |
| hsa-miR-940 | 1.540 | 0.558 | 2.106E-05 | Neerincx et al. |
| hsa-miR-4662a-5p | -1.929 | 1.692 | 2.400E-05 | Neerincx et al. |
| hsa-miR-338-3p | -1.618 | 6.262 | 2.403E-05 | Neerincx et al. |
| hsa-miR-1-3p | -2.377 | 3.625 | 2.440E-05 | Neerincx et al. |
| hsa-miR-29b-2-5p | -0.960 | 0.761 | 2.507E-05 | Neerincx et al. |
| hsa-miR-25-3p | 0.696 | 10.835 | 3.396E-05 | Neerincx et al. |
| hsa-miR-3200-3p | 1.596 | 1.342 | 3.409E-05 | Neerincx et al. |
| hsa-miR-142-5p | -1.255 | 11.237 | 3.691E-05 | Neerincx et al. |
| hsa-miR-493-5p | 1.287 | 3.828 | 4.065E-05 | Neerincx et al. |
| hsa-miR-5571-3p | -2.236 | 0.339 | 4.646E-05 | Neerincx et al. |
| hsa-miR-5690 | -1.199 | 0.080 | 4.646E-05 | Neerincx et al. |
| hsa-miR-93-5p | 0.698 | 10.132 | 6.234E-05 | Neerincx et al. |
| hsa-miR-10b-3p | -1.322 | 2.511 | 6.511E-05 | Neerincx et al. |
| hsa-miR-30c-2-3p | -1.146 | 2.262 | 6.511E-05 | Neerincx et al. |
| hsa-miR-3687 | 2.120 | 2.971 | 6.511E-05 | Neerincx et al. |
| hsa-miR-21-5p | 0.966 | 15.574 | 6.511E-05 | Neerincx et al. |
| hsa-miR-186-5p | -0.797 | 11.255 | 6.511E-05 | Neerincx et al. |
| hsa-miR-363-3p | -1.606 | 4.818 | 6.511E-05 | Neerincx et al. |
| hsa-miR-342-5p | -1.128 | 1.915 | 7.009E-05 | Neerincx et al. |
| hsa-miR-192-5p | -1.356 | 17.315 | 7.222E-05 | Neerincx et al. |
| hsa-miR-26b-3p | -0.799 | 3.799 | 7.520E-05 | Neerincx et al. |
| hsa-miR-598-3p | -1.239 | 3.719 | 7.829E-05 | Neerincx et al. |
| hsa-miR-20a-5p | 1.031 | 9.205 | 8.157E-05 | Neerincx et al. |
| hsa-miR-6125 | -1.239 | 0.702 | 1.492E-04 | Neerincx et al. |
| hsa-miR-192-3p | -1.224 | 4.387 | 1.548E-04 | Neerincx et al. |
| hsa-miR-218-5p | -1.405 | 4.643 | 1.695E-04 | Neerincx et al. |
| hsa-miR-30b-5p | -0.970 | 9.838 | 1.695E-04 | Neerincx et al. |
| hsa-miR-144-5p | -1.364 | 4.968 | 2.049E-04 | Neerincx et al. |
| hsa-miR-22-3p | -0.695 | 14.357 | 2.185E-04 | Neerincx et al. |
| hsa-miR-1247-5p | 1.886 | 4.182 | 2.363E-04 | Neerincx et al. |
| hsa-miR-28-3p | -0.883 | 11.574 | 2.396E-04 | Neerincx et al. |
| hsa-miR-628-5p | -0.925 | 2.216 | 2.421E-04 | Neerincx et al. |
| hsa-miR-887-5p | -1.006 | 0.180 | 3.300E-04 | Neerincx et al. |
| hsa-miR-381-3p | -0.872 | 7.420 | 3.930E-04 | Neerincx et al. |
| hsa-miR-338-5p | -1.265 | 2.443 | 4.037E-04 | Neerincx et al. |
| hsa-miR-3176 | 1.565 | 1.415 | 4.471E-04 | Neerincx et al. |
| hsa-miR-877-5p | 1.229 | 2.094 | 5.239E-04 | Neerincx et al. |
| hsa-miR-451a | -1.395 | 9.027 | 5.385E-04 | Neerincx et al. |
| hsa-miR-19a-5p | 1.242 | 0.836 | 5.385E-04 | Neerincx et al. |
| hsa-miR-887-3p | -0.761 | 3.496 | 5.492E-04 | Neerincx et al. |
| hsa-miR-106b-3p | 0.585 | 7.146 | 5.643E-04 | Neerincx et al. |
| hsa-miR-450b-5p | 1.030 | 4.188 | 5.643E-04 | Neerincx et al. |
| hsa-miR-95-3p | 0.952 | 4.537 | 6.518E-04 | Neerincx et al. |
| hsa-miR-194-3p | -1.276 | 4.721 | 7.254E-04 | Neerincx et al. |
| hsa-miR-3651 | 1.644 | 0.537 | 7.404E-04 | Neerincx et al. |
| hsa-miR-628-3p | -0.821 | 1.581 | 7.454E-04 | Neerincx et al. |
| hsa-miR-1468-5p | -0.938 | 3.495 | 7.454E-04 | Neerincx et al. |
| hsa-miR-145-5p | -1.293 | 9.071 | 7.498E-04 | Neerincx et al. |
| hsa-miR-25-5p | 0.953 | 2.683 | 7.563E-04 | Neerincx et al. |
| hsa-miR-18a-5p | 1.070 | 5.769 | 7.622E-04 | Neerincx et al. |
| hsa-miR-136-5p | -0.990 | 5.030 | 7.755E-04 | Neerincx et al. |
| hsa-miR-574-3p | -0.674 | 7.306 | 8.525E-04 | Neerincx et al. |
| hsa-miR-3912-3p | -0.762 | 1.744 | 9.526E-04 | Neerincx et al. |
| hsa-miR-20b-5p | -1.548 | 1.604 | 9.575E-04 | Neerincx et al. |
| hsa-miR-144-3p | -1.287 | 6.104 | 9.620E-04 | Neerincx et al. |
| hsa-miR-421 | 0.755 | 6.021 | 9.942E-04 | Neerincx et al. |
| hsa-miR-671-5p | 0.658 | 2.374 | 1.053E-03 | Neerincx et al. |
| hsa-miR-150-5p | -1.335 | 7.486 | 1.136E-03 | Neerincx et al. |
| hsa-miR-16-5p | -0.630 | 12.467 | 1.136E-03 | Neerincx et al. |
| hsa-miR-424-5p | 1.294 | 5.786 | 1.373E-03 | Neerincx et al. |
| hsa-miR-200a-3p | -0.682 | 10.473 | 1.417E-03 | Neerincx et al. |
| hsa-miR-15a-5p | -0.631 | 8.449 | 1.426E-03 | Neerincx et al. |
| hsa-miR-320c | 0.914 | 2.846 | 1.464E-03 | Neerincx et al. |
| hsa-miR-592 | 1.676 | 4.932 | 1.505E-03 | Neerincx et al. |
| hsa-miR-504-5p | -1.270 | 1.065 | 1.520E-03 | Neerincx et al. |
| hsa-miR-550a-5p | 0.854 | 2.071 | 1.708E-03 | Neerincx et al. |
| hsa-miR-218-1-3p | -1.375 | 1.916 | 1.733E-03 | Neerincx et al. |
| hsa-miR-3913-5p | 0.815 | 1.224 | 1.836E-03 | Neerincx et al. |
| hsa-miR-143-5p | -1.086 | 3.519 | 2.022E-03 | Neerincx et al. |
| hsa-miR-7-5p | 1.350 | 5.405 | 2.062E-03 | Neerincx et al. |
| hsa-miR-4532 | -1.471 | 0.690 | 2.142E-03 | Neerincx et al. |
| hsa-miR-382-5p | 0.704 | 2.496 | 2.166E-03 | Neerincx et al. |
| hsa-miR-2467-5p | 0.826 | 2.207 | 2.194E-03 | Neerincx et al. |
| hsa-miR-542-3p | 0.952 | 3.725 | 2.232E-03 | Neerincx et al. |
| hsa-miR-197-3p | -0.497 | 6.983 | 2.497E-03 | Neerincx et al. |
| hsa-miR-450a-5p | 0.965 | 3.227 | 2.565E-03 | Neerincx et al. |
| hsa-miR-7704 | -0.824 | 2.995 | 2.565E-03 | Neerincx et al. |
| hsa-let-7g-5p | -0.588 | 12.229 | 2.865E-03 | Neerincx et al. |
| hsa-miR-181c-5p | 0.646 | 9.633 | 3.132E-03 | Neerincx et al. |
| hsa-miR-663b | 1.552 | 0.848 | 3.489E-03 | Neerincx et al. |
| hsa-miR-10b-5p | -1.196 | 15.443 | 3.489E-03 | Neerincx et al. |
| hsa-miR-130b-3p | 0.582 | 6.105 | 3.632E-03 | Neerincx et al. |
| hsa-miR-101-5p | -0.755 | 3.307 | 3.796E-03 | Neerincx et al. |
| hsa-miR-1185-1-3p | 0.924 | 1.060 | 3.796E-03 | Neerincx et al. |
| hsa-miR-490-3p | -2.507 | 0.740 | 3.874E-03 | Neerincx et al. |
| hsa-miR-140-5p | -0.785 | 4.057 | 3.970E-03 | Neerincx et al. |
| hsa-miR-361-3p | -0.518 | 6.521 | 4.030E-03 | Neerincx et al. |
| hsa-miR-328-3p | -0.586 | 2.649 | 4.073E-03 | Neerincx et al. |
| hsa-miR-30d-5p | -0.538 | 12.533 | 4.160E-03 | Neerincx et al. |
| hsa-miR-627-5p | -0.858 | 0.645 | 4.262E-03 | Neerincx et al. |
| hsa-miR-330-3p | -0.596 | 1.814 | 4.399E-03 | Neerincx et al. |
| hsa-miR-376c-3p | -0.765 | 2.209 | 4.431E-03 | Neerincx et al. |
| hsa-miR-1275 | 1.276 | 3.685 | 4.437E-03 | Neerincx et al. |
| hsa-miR-362-3p | -0.872 | 1.022 | 4.587E-03 | Neerincx et al. |
| hsa-miR-342-3p | -0.755 | 7.095 | 4.587E-03 | Neerincx et al. |
| hsa-miR-340-3p | -0.726 | 2.474 | 4.693E-03 | Neerincx et al. |
| hsa-let-7i-5p | 0.489 | 11.154 | 4.730E-03 | Neerincx et al. |
| hsa-miR-30b-3p | -0.764 | 1.967 | 4.990E-03 | Neerincx et al. |
| hsa-miR-140-3p | -0.525 | 8.457 | 4.993E-03 | Neerincx et al. |
| hsa-let-7g-3p | -0.614 | 1.602 | 5.106E-03 | Neerincx et al. |
| hsa-miR-95-5p | 1.159 | 0.707 | 5.287E-03 | Neerincx et al. |
| hsa-miR-339-5p | 0.503 | 5.155 | 5.939E-03 | Neerincx et al. |
| hsa-miR-17-3p | 0.644 | 5.117 | 6.601E-03 | Neerincx et al. |
| hsa-miR-429 | -0.609 | 10.018 | 6.601E-03 | Neerincx et al. |
| hsa-miR-22-5p | -0.612 | 4.058 | 6.721E-03 | Neerincx et al. |
| hsa-miR-145-3p | -1.055 | 7.239 | 7.056E-03 | Neerincx et al. |
| hsa-miR-4485-5p | 1.489 | 1.066 | 7.210E-03 | Neerincx et al. |
| hsa-miR-3609 | 1.201 | 2.819 | 7.262E-03 | Neerincx et al. |
| hsa-miR-4485-3p | 1.852 | 4.154 | 7.421E-03 | Neerincx et al. |
| hsa-miR-150-3p | -1.089 | 0.776 | 8.083E-03 | Neerincx et al. |
| hsa-miR-3677-3p | 1.021 | 0.096 | 8.376E-03 | Neerincx et al. |
| hsa-miR-552-3p | 1.549 | 4.077 | 8.433E-03 | Neerincx et al. |
| hsa-miR-616-5p | -0.812 | 0.702 | 9.745E-03 | Neerincx et al. |
| hsa-miR-495-3p | -0.685 | 1.709 | 1.019E-02 | Neerincx et al. |
| hsa-miR-326 | -0.813 | 0.208 | 1.029E-02 | Neerincx et al. |
| hsa-miR-452-3p | 0.817 | 3.276 | 1.043E-02 | Neerincx et al. |
| hsa-miR-26a-2-3p | -0.705 | 2.992 | 1.043E-02 | Neerincx et al. |
| hsa-miR-20a-3p | 0.727 | 1.558 | 1.072E-02 | Neerincx et al. |
| hsa-miR-134-5p | 0.682 | 3.230 | 1.093E-02 | Neerincx et al. |
| hsa-miR-320a | -0.449 | 8.816 | 1.137E-02 | Neerincx et al. |
| hsa-miR-19b-3p | 0.661 | 9.115 | 1.150E-02 | Neerincx et al. |
| hsa-miR-33b-5p | -0.677 | 4.774 | 1.171E-02 | Neerincx et al. |
| hsa-miR-7-1-3p | -0.618 | 3.184 | 1.205E-02 | Neerincx et al. |
| hsa-miR-30d-3p | -0.629 | 5.422 | 1.205E-02 | Neerincx et al. |
| hsa-miR-210-5p | -0.886 | 2.568 | 1.207E-02 | Neerincx et al. |
| hsa-miR-320d | 0.770 | 2.409 | 1.267E-02 | Neerincx et al. |
| hsa-miR-23b-3p | -0.602 | 10.043 | 1.285E-02 | Neerincx et al. |
| hsa-miR-142-3p | -0.905 | 7.521 | 1.316E-02 | Neerincx et al. |
| hsa-miR-409-3p | 0.498 | 6.865 | 1.325E-02 | Neerincx et al. |
| hsa-miR-378e | -1.601 | 0.541 | 1.380E-02 | Neerincx et al. |
| hsa-miR-301b-3p | 1.000 | 4.970 | 1.421E-02 | Neerincx et al. |
| hsa-miR-204-5p | -1.737 | 4.187 | 1.421E-02 | Neerincx et al. |
| hsa-miR-155-5p | -0.538 | 7.701 | 1.421E-02 | Neerincx et al. |
| hsa-miR-1285-3p | 1.101 | 2.006 | 1.445E-02 | Neerincx et al. |
| hsa-miR-132-5p | -0.494 | 2.576 | 1.472E-02 | Neerincx et al. |
| hsa-miR-576-5p | -0.570 | 3.063 | 1.547E-02 | Neerincx et al. |
| hsa-miR-92b-3p | 0.678 | 9.573 | 1.683E-02 | Neerincx et al. |
| hsa-miR-214-3p | 0.644 | 6.148 | 1.712E-02 | Neerincx et al. |
| hsa-miR-455-3p | 0.627 | 3.789 | 1.933E-02 | Neerincx et al. |
| hsa-miR-32-5p | -0.574 | 5.493 | 1.933E-02 | Neerincx et al. |
| hsa-miR-24-1-5p | -0.590 | 1.846 | 1.991E-02 | Neerincx et al. |
| hsa-miR-181b-5p | 0.407 | 9.276 | 1.991E-02 | Neerincx et al. |
| hsa-miR-149-5p | -0.683 | 4.021 | 2.182E-02 | Neerincx et al. |
| hsa-miR-16-2-3p | -0.493 | 5.143 | 2.375E-02 | Neerincx et al. |
| hsa-miR-7705 | 0.574 | 3.290 | 2.506E-02 | Neerincx et al. |
| hsa-miR-532-5p | 0.519 | 8.679 | 2.558E-02 | Neerincx et al. |
| hsa-miR-653-5p | -0.914 | 1.525 | 2.814E-02 | Neerincx et al. |
| hsa-miR-345-5p | 0.509 | 7.102 | 3.148E-02 | Neerincx et al. |
| hsa-miR-489-3p | -0.943 | 0.879 | 3.306E-02 | Neerincx et al. |
| hsa-miR-3648 | 1.298 | 2.449 | 3.452E-02 | Neerincx et al. |
| hsa-miR-23a-3p | 0.327 | 9.587 | 3.567E-02 | Neerincx et al. |
| hsa-miR-542-5p | 0.630 | 0.442 | 3.727E-02 | Neerincx et al. |
| hsa-miR-19a-3p | 0.637 | 7.037 | 3.858E-02 | Neerincx et al. |
| hsa-miR-1291 | 0.759 | 1.194 | 3.934E-02 | Neerincx et al. |
| hsa-miR-561-5p | -0.729 | 3.432 | 3.966E-02 | Neerincx et al. |
| hsa-miR-431-3p | 0.575 | 1.432 | 3.966E-02 | Neerincx et al. |
| hsa-miR-627-3p | -0.574 | 0.429 | 3.966E-02 | Neerincx et al. |
| hsa-miR-222-3p | 0.471 | 9.472 | 3.966E-02 | Neerincx et al. |
| hsa-miR-548e-3p | -0.533 | 1.979 | 4.172E-02 | Neerincx et al. |
| hsa-miR-501-5p | 0.557 | 1.967 | 4.400E-02 | Neerincx et al. |
| hsa-miR-153-3p | -0.758 | 4.312 | 4.512E-02 | Neerincx et al. |
| hsa-miR-582-5p | -0.627 | 4.043 | 4.522E-02 | Neerincx et al. |
| hsa-miR-136-3p | -0.532 | 6.662 | 4.572E-02 | Neerincx et al. |
| hsa-miR-98-5p | 0.497 | 10.315 | 4.651E-02 | Neerincx et al. |
| hsa-miR-29a-5p | 0.564 | 2.489 | 4.705E-02 | Neerincx et al. |
| hsa-miR-92b-5p | 0.714 | 0.213 | 4.705E-02 | Neerincx et al. |
| hsa-miR-324-3p | 0.426 | 2.945 | 4.842E-02 | Neerincx et al. |
| hsa-miR-4746-5p | 0.580 | 0.514 | 4.929E-02 | Neerincx et al. |
| hsa-miR-200b-5p | -0.431 | 5.515 | 4.969E-02 | Neerincx et al. |
| hsa-miR-374a-5p | -0.553 | 6.579 | 5.046E-02 | Neerincx et al. |
| hsa-miR-29b-1-5p | 0.652 | 1.498 | 5.082E-02 | Neerincx et al. |
| hsa-miR-340-5p | -0.414 | 8.177 | 5.089E-02 | Neerincx et al. |
| hsa-miR-1307-5p | -0.488 | 9.274 | 5.089E-02 | Neerincx et al. |
| hsa-miR-126-3p | -0.541 | 9.879 | 5.367E-02 | Neerincx et al. |
| hsa-miR-552-5p | 1.110 | 3.452 | 5.618E-02 | Neerincx et al. |
| hsa-miR-200b-3p | -0.408 | 11.809 | 5.885E-02 | Neerincx et al. |
| hsa-miR-34a-5p | 0.451 | 7.322 | 5.923E-02 | Neerincx et al. |
| hsa-miR-143-3p | -0.678 | 16.853 | 5.923E-02 | Neerincx et al. |
| hsa-miR-101-3p | -0.503 | 10.995 | 5.973E-02 | Neerincx et al. |
| hsa-miR-99b-3p | 0.543 | 2.835 | 6.138E-02 | Neerincx et al. |
| hsa-miR-744-3p | -0.397 | 1.728 | 6.703E-02 | Neerincx et al. |
| hsa-miR-4449 | 1.067 | 1.041 | 6.848E-02 | Neerincx et al. |
| hsa-miR-484 | -0.364 | 7.307 | 6.996E-02 | Neerincx et al. |
| hsa-miR-34a-3p | 0.538 | 0.213 | 7.375E-02 | Neerincx et al. |
| hsa-miR-10a-3p | 0.407 | 5.611 | 7.375E-02 | Neerincx et al. |
| hsa-miR-411-5p | -0.457 | 8.119 | 7.613E-02 | Neerincx et al. |
| hsa-miR-93-3p | 0.372 | 3.107 | 7.677E-02 | Neerincx et al. |
| hsa-miR-941 | 0.629 | 8.056 | 7.949E-02 | Neerincx et al. |
| hsa-miR-221-5p | 0.553 | 4.763 | 8.140E-02 | Neerincx et al. |
| hsa-miR-382-3p | 0.559 | 1.447 | 8.508E-02 | Neerincx et al. |
| hsa-miR-6087 | 1.105 | 4.906 | 8.779E-02 | Neerincx et al. |
| hsa-miR-126-5p | -0.476 | 11.704 | 8.792E-02 | Neerincx et al. |
| hsa-let-7i-3p | -0.415 | 4.638 | 8.826E-02 | Neerincx et al. |
| hsa-miR-376a-5p | -0.881 | 1.729 | 8.826E-02 | Neerincx et al. |
| hsa-miR-125b-1-3p | 0.554 | 3.895 | 8.826E-02 | Neerincx et al. |
| hsa-miR-486-5p | -0.759 | 10.525 | 8.826E-02 | Neerincx et al. |
| hsa-miR-151a-3p | 0.382 | 10.995 | 8.826E-02 | Neerincx et al. |
| hsa-miR-320b | 0.431 | 5.214 | 9.070E-02 | Neerincx et al. |
| hsa-miR-27b-3p | -0.325 | 14.587 | 9.072E-02 | Neerincx et al. |
| hsa-miR-34c-5p | 0.508 | 3.773 | 9.271E-02 | Neerincx et al. |
| hsa-miR-24-3p | 0.437 | 9.006 | 9.578E-02 | Neerincx et al. |
| hsa-miR-3614-5p | -0.687 | 1.431 | 9.592E-02 | Neerincx et al. |
| hsa-miR-659-5p | -0.406 | 1.022 | 9.617E-02 | Neerincx et al. |
| hsa-miR-1248 | 1.181 | 1.919 | 9.699E-02 | Neerincx et al. |
| hsa-miR-664a-5p | -0.419 | 2.195 | 1.012E-01 | Neerincx et al. |
| hsa-let-7f-5p | -0.333 | 13.687 | 1.012E-01 | Neerincx et al. |
| hsa-miR-409-5p | 0.929 | 0.858 | 1.053E-01 | Neerincx et al. |
| hsa-miR-125a-5p | -0.519 | 10.800 | 1.091E-01 | Neerincx et al. |
| hsa-miR-1287-5p | -0.388 | 1.641 | 1.105E-01 | Neerincx et al. |
| hsa-miR-106b-5p | 0.315 | 7.180 | 1.105E-01 | Neerincx et al. |
| hsa-miR-500b-5p | 0.378 | 2.257 | 1.108E-01 | Neerincx et al. |
| hsa-miR-200a-5p | -0.391 | 6.807 | 1.116E-01 | Neerincx et al. |
| hsa-miR-548e-5p | -0.393 | 1.279 | 1.156E-01 | Neerincx et al. |
| hsa-miR-873-5p | -0.617 | 0.310 | 1.200E-01 | Neerincx et al. |
| hsa-miR-224-3p | 0.437 | 1.567 | 1.200E-01 | Neerincx et al. |
| hsa-miR-1260b | 0.718 | 2.809 | 1.202E-01 | Neerincx et al. |
| hsa-miR-500a-5p | 0.373 | 2.274 | 1.207E-01 | Neerincx et al. |
| hsa-miR-652-3p | 0.317 | 5.144 | 1.224E-01 | Neerincx et al. |
| hsa-miR-3653-5p | -0.515 | 0.767 | 1.294E-01 | Neerincx et al. |
| hsa-miR-7706 | 0.548 | 4.417 | 1.319E-01 | Neerincx et al. |
| hsa-miR-18b-5p | 0.582 | 0.242 | 1.342E-01 | Neerincx et al. |
| hsa-miR-487b-3p | -0.395 | 3.543 | 1.350E-01 | Neerincx et al. |
| hsa-miR-769-5p | 0.292 | 7.721 | 1.361E-01 | Neerincx et al. |
| hsa-miR-10a-5p | 0.493 | 16.663 | 1.361E-01 | Neerincx et al. |
| hsa-miR-502-5p | -0.348 | 1.487 | 1.361E-01 | Neerincx et al. |
| hsa-miR-130a-3p | -0.357 | 8.030 | 1.455E-01 | Neerincx et al. |
| hsa-miR-548i | -0.920 | 0.192 | 1.474E-01 | Neerincx et al. |
| hsa-miR-331-5p | 0.273 | 1.837 | 1.482E-01 | Neerincx et al. |
| hsa-miR-221-3p | 0.281 | 9.895 | 1.578E-01 | Neerincx et al. |
| hsa-miR-556-5p | -0.441 | 0.306 | 1.588E-01 | Neerincx et al. |
| hsa-miR-107 | 0.310 | 8.866 | 1.656E-01 | Neerincx et al. |
| hsa-miR-744-5p | -0.263 | 5.711 | 1.670E-01 | Neerincx et al. |
| hsa-miR-1301-3p | 0.329 | 2.838 | 1.702E-01 | Neerincx et al. |
| hsa-miR-200c-3p | -0.315 | 10.896 | 1.708E-01 | Neerincx et al. |
| hsa-miR-24-2-5p | 0.344 | 4.649 | 1.722E-01 | Neerincx et al. |
| hsa-miR-1247-3p | 0.776 | 3.055 | 1.729E-01 | Neerincx et al. |
| hsa-miR-141-5p | -0.337 | 7.293 | 1.729E-01 | Neerincx et al. |
| hsa-miR-323b-3p | 0.439 | 0.938 | 1.736E-01 | Neerincx et al. |
| hsa-miR-548o-3p | 0.296 | 3.023 | 1.750E-01 | Neerincx et al. |
| hsa-miR-577 | -0.436 | 6.805 | 1.770E-01 | Neerincx et al. |
| hsa-miR-455-5p | 0.371 | 5.592 | 1.792E-01 | Neerincx et al. |
| hsa-miR-152-3p | -0.333 | 5.039 | 1.810E-01 | Neerincx et al. |
| hsa-miR-766-3p | -0.354 | 1.596 | 1.893E-01 | Neerincx et al. |
| hsa-miR-758-3p | 0.409 | 1.055 | 1.910E-01 | Neerincx et al. |
| hsa-miR-337-3p | -0.376 | 1.925 | 1.943E-01 | Neerincx et al. |
| hsa-miR-27a-5p | 0.468 | 4.923 | 1.951E-01 | Neerincx et al. |
| hsa-miR-32-3p | -0.289 | 2.299 | 1.951E-01 | Neerincx et al. |
| hsa-miR-223-5p | -0.462 | 1.698 | 1.960E-01 | Neerincx et al. |
| hsa-miR-454-5p | 0.357 | 1.660 | 2.215E-01 | Neerincx et al. |
| hsa-miR-3065-3p | -0.446 | 0.636 | 2.216E-01 | Neerincx et al. |
| hsa-miR-665 | 0.421 | 1.603 | 2.216E-01 | Neerincx et al. |
| hsa-let-7b-3p | -0.240 | 3.937 | 2.261E-01 | Neerincx et al. |
| hsa-miR-1277-5p | -0.337 | 2.406 | 2.300E-01 | Neerincx et al. |
| hsa-miR-122-5p | -0.926 | 2.389 | 2.423E-01 | Neerincx et al. |
| hsa-miR-1180-3p | 0.313 | 3.568 | 2.437E-01 | Neerincx et al. |
| hsa-miR-299-3p | -0.314 | 3.046 | 2.437E-01 | Neerincx et al. |
| hsa-miR-181a-3p | 0.310 | 6.016 | 2.453E-01 | Neerincx et al. |
| hsa-miR-339-3p | 0.219 | 6.072 | 2.453E-01 | Neerincx et al. |
| hsa-miR-196a-5p | -0.446 | 7.292 | 2.453E-01 | Neerincx et al. |
| hsa-miR-1249-3p | 0.314 | 0.934 | 2.481E-01 | Neerincx et al. |
| hsa-miR-141-3p | -0.367 | 13.199 | 2.493E-01 | Neerincx et al. |
| hsa-miR-664b-3p | 0.399 | 0.100 | 2.542E-01 | Neerincx et al. |
| hsa-miR-370-3p | 0.413 | 1.714 | 2.589E-01 | Neerincx et al. |
| hsa-miR-127-5p | 0.289 | 2.902 | 2.589E-01 | Neerincx et al. |
| hsa-miR-651-5p | -0.379 | 2.353 | 2.589E-01 | Neerincx et al. |
| hsa-miR-369-5p | -0.309 | 2.792 | 2.589E-01 | Neerincx et al. |
| hsa-miR-214-5p | 0.313 | 4.985 | 2.607E-01 | Neerincx et al. |
| hsa-miR-4775 | -0.363 | 0.791 | 2.644E-01 | Neerincx et al. |
| hsa-miR-590-3p | -0.311 | 3.976 | 2.708E-01 | Neerincx et al. |
| hsa-miR-188-5p | 0.335 | 2.764 | 2.729E-01 | Neerincx et al. |
| hsa-miR-425-3p | 0.242 | 4.336 | 2.794E-01 | Neerincx et al. |
| hsa-miR-27a-3p | 0.205 | 11.308 | 2.804E-01 | Neerincx et al. |
| hsa-miR-550a-3p | 0.310 | 2.333 | 2.827E-01 | Neerincx et al. |
| hsa-miR-874-3p | 0.406 | 4.626 | 2.957E-01 | Neerincx et al. |
| hsa-let-7a-5p | -0.284 | 14.545 | 2.976E-01 | Neerincx et al. |
| hsa-miR-33b-3p | -0.405 | 0.334 | 3.070E-01 | Neerincx et al. |
| hsa-miR-3615 | 0.279 | 3.452 | 3.082E-01 | Neerincx et al. |
| hsa-miR-548k | -0.259 | 3.941 | 3.160E-01 | Neerincx et al. |
| hsa-let-7b-5p | -0.200 | 11.609 | 3.167E-01 | Neerincx et al. |
| hsa-let-7e-3p | -0.317 | 1.452 | 3.206E-01 | Neerincx et al. |
| hsa-miR-125b-2-3p | 0.444 | 4.825 | 3.234E-01 | Neerincx et al. |
| hsa-miR-324-5p | 0.210 | 4.531 | 3.472E-01 | Neerincx et al. |
| hsa-miR-543 | -0.290 | 0.807 | 3.539E-01 | Neerincx et al. |
| hsa-miR-454-3p | -0.181 | 6.654 | 3.647E-01 | Neerincx et al. |
| hsa-miR-425-5p | 0.180 | 9.116 | 3.647E-01 | Neerincx et al. |
| hsa-miR-181a-5p | 0.185 | 13.858 | 3.677E-01 | Neerincx et al. |
| hsa-miR-185-3p | -0.259 | 0.802 | 3.699E-01 | Neerincx et al. |
| hsa-let-7d-3p | 0.161 | 6.890 | 3.725E-01 | Neerincx et al. |
| hsa-miR-146b-3p | -0.241 | 5.557 | 3.762E-01 | Neerincx et al. |
| hsa-miR-664a-3p | -0.236 | 3.349 | 3.764E-01 | Neerincx et al. |
| hsa-miR-486-3p | -0.293 | 1.376 | 3.872E-01 | Neerincx et al. |
| hsa-miR-2355-5p | 0.241 | 1.598 | 3.872E-01 | Neerincx et al. |
| hsa-miR-130b-5p | 0.200 | 4.114 | 3.872E-01 | Neerincx et al. |
| hsa-miR-499a-5p | 0.484 | 1.849 | 3.978E-01 | Neerincx et al. |
| hsa-miR-200c-5p | -0.295 | 1.305 | 4.074E-01 | Neerincx et al. |
| hsa-miR-3158-3p | 0.227 | 1.896 | 4.203E-01 | Neerincx et al. |
| hsa-miR-1306-5p | -0.239 | 0.618 | 4.327E-01 | Neerincx et al. |
| hsa-miR-411-3p | -0.236 | 0.881 | 4.608E-01 | Neerincx et al. |
| hsa-miR-377-3p | -0.250 | 1.787 | 4.626E-01 | Neerincx et al. |
| hsa-miR-203a-3p | 0.223 | 9.581 | 4.626E-01 | Neerincx et al. |
| hsa-miR-361-5p | 0.166 | 6.875 | 4.626E-01 | Neerincx et al. |
| hsa-miR-3909 | 0.163 | 2.717 | 4.626E-01 | Neerincx et al. |
| hsa-miR-3613-3p | -0.220 | 0.543 | 4.626E-01 | Neerincx et al. |
| hsa-miR-4791 | 0.297 | 0.668 | 4.626E-01 | Neerincx et al. |
| hsa-miR-5701 | 0.506 | 2.969 | 4.665E-01 | Neerincx et al. |
| hsa-miR-374b-5p | -0.180 | 5.685 | 4.731E-01 | Neerincx et al. |
| hsa-miR-337-5p | -0.247 | 1.326 | 4.731E-01 | Neerincx et al. |
| hsa-miR-3653-3p | -0.364 | 1.432 | 4.731E-01 | Neerincx et al. |
| hsa-miR-148b-3p | 0.226 | 8.022 | 4.731E-01 | Neerincx et al. |
| hsa-miR-377-5p | -0.204 | 1.141 | 4.746E-01 | Neerincx et al. |
| hsa-miR-154-5p | -0.235 | 1.535 | 4.865E-01 | Neerincx et al. |
| hsa-miR-502-3p | -0.140 | 4.880 | 5.064E-01 | Neerincx et al. |
| hsa-let-7d-5p | -0.168 | 9.544 | 5.068E-01 | Neerincx et al. |
| hsa-miR-223-3p | 0.282 | 7.628 | 5.071E-01 | Neerincx et al. |
| hsa-miR-656-3p | -0.277 | 0.999 | 5.091E-01 | Neerincx et al. |
| hsa-miR-629-5p | 0.184 | 3.901 | 5.091E-01 | Neerincx et al. |
| hsa-miR-301a-3p | 0.275 | 7.312 | 5.110E-01 | Neerincx et al. |
| hsa-miR-33a-3p | -0.194 | 1.096 | 5.220E-01 | Neerincx et al. |
| hsa-miR-487a-3p | 0.208 | 0.883 | 5.347E-01 | Neerincx et al. |
| hsa-miR-374a-3p | -0.209 | 7.148 | 5.359E-01 | Neerincx et al. |
| hsa-miR-589-5p | 0.157 | 5.002 | 5.396E-01 | Neerincx et al. |
| hsa-miR-296-5p | -0.339 | 1.189 | 5.400E-01 | Neerincx et al. |
| hsa-miR-1266-5p | -0.256 | 0.266 | 5.510E-01 | Neerincx et al. |
| hsa-miR-106a-5p | -0.283 | 2.813 | 5.510E-01 | Neerincx et al. |
| hsa-miR-3607-3p | -0.404 | 4.697 | 5.518E-01 | Neerincx et al. |
| hsa-miR-671-3p | -0.149 | 4.691 | 5.522E-01 | Neerincx et al. |
| hsa-let-7e-5p | -0.198 | 9.486 | 5.766E-01 | Neerincx et al. |
| hsa-miR-539-3p | -0.155 | 2.282 | 5.858E-01 | Neerincx et al. |
| hsa-miR-3613-5p | -0.164 | 3.676 | 5.890E-01 | Neerincx et al. |
| hsa-miR-127-3p | 0.175 | 10.690 | 6.022E-01 | Neerincx et al. |
| hsa-miR-4516 | -0.462 | 2.331 | 6.050E-01 | Neerincx et al. |
| hsa-miR-199b-3p | 0.164 | 11.669 | 6.130E-01 | Neerincx et al. |
| hsa-miR-199a-3p | 0.163 | 11.670 | 6.132E-01 | Neerincx et al. |
| hsa-miR-2116-3p | -0.195 | 0.115 | 6.137E-01 | Neerincx et al. |
| hsa-miR-660-5p | -0.131 | 7.585 | 6.137E-01 | Neerincx et al. |
| hsa-miR-27b-5p | -0.122 | 5.209 | 6.152E-01 | Neerincx et al. |
| hsa-miR-15b-3p | 0.134 | 3.802 | 6.204E-01 | Neerincx et al. |
| hsa-miR-2110 | -0.164 | 1.423 | 6.242E-01 | Neerincx et al. |
| hsa-miR-128-1-5p | 0.180 | 1.949 | 6.242E-01 | Neerincx et al. |
| hsa-miR-494-3p | 0.256 | 1.070 | 6.242E-01 | Neerincx et al. |
| hsa-miR-365a-3p | 0.126 | 4.524 | 6.263E-01 | Neerincx et al. |
| hsa-miR-1307-3p | 0.127 | 6.453 | 6.326E-01 | Neerincx et al. |
| hsa-miR-365b-3p | 0.123 | 4.525 | 6.338E-01 | Neerincx et al. |
| hsa-miR-576-3p | -0.188 | 1.002 | 6.338E-01 | Neerincx et al. |
| hsa-miR-654-5p | 0.195 | 0.583 | 6.419E-01 | Neerincx et al. |
| hsa-miR-423-5p | -0.134 | 8.311 | 6.452E-01 | Neerincx et al. |
| hsa-miR-4326 | 0.266 | 1.087 | 6.452E-01 | Neerincx et al. |
| hsa-miR-132-3p | -0.135 | 6.206 | 6.471E-01 | Neerincx et al. |
| hsa-miR-532-3p | -0.116 | 3.808 | 6.484E-01 | Neerincx et al. |
| hsa-miR-99a-3p | 0.225 | 1.836 | 6.497E-01 | Neerincx et al. |
| hsa-miR-6724-5p | 0.181 | 0.772 | 6.497E-01 | Neerincx et al. |
| hsa-miR-4488 | 0.381 | 0.731 | 6.497E-01 | Neerincx et al. |
| hsa-miR-3116 | -0.159 | 0.481 | 6.551E-01 | Neerincx et al. |
| hsa-miR-1296-5p | -0.114 | 4.217 | 6.772E-01 | Neerincx et al. |
| hsa-let-7f-2-3p | 0.158 | 0.165 | 6.785E-01 | Neerincx et al. |
| hsa-miR-128-3p | 0.073 | 6.634 | 6.837E-01 | Neerincx et al. |
| hsa-miR-485-5p | 0.124 | 1.188 | 6.855E-01 | Neerincx et al. |
| hsa-miR-193b-3p | 0.120 | 6.220 | 6.907E-01 | Neerincx et al. |
| hsa-miR-212-5p | 0.157 | 1.883 | 7.049E-01 | Neerincx et al. |
| hsa-miR-3065-5p | 0.211 | 0.529 | 7.066E-01 | Neerincx et al. |
| hsa-miR-942-5p | 0.121 | 1.167 | 7.128E-01 | Neerincx et al. |
| hsa-miR-369-3p | -0.134 | 3.093 | 7.189E-01 | Neerincx et al. |
| hsa-miR-199b-5p | -0.146 | 8.286 | 7.194E-01 | Neerincx et al. |
| hsa-miR-181a-2-3p | -0.104 | 6.853 | 7.194E-01 | Neerincx et al. |
| hsa-miR-376b-3p | 0.129 | 0.396 | 7.253E-01 | Neerincx et al. |
| hsa-miR-212-3p | -0.120 | 3.162 | 7.253E-01 | Neerincx et al. |
| hsa-miR-125b-5p | 0.158 | 8.479 | 7.334E-01 | Neerincx et al. |
| hsa-miR-152-5p | -0.101 | 1.874 | 7.375E-01 | Neerincx et al. |
| hsa-miR-4461 | -0.181 | 0.288 | 7.377E-01 | Neerincx et al. |
| hsa-miR-501-3p | 0.186 | 6.637 | 7.409E-01 | Neerincx et al. |
| hsa-miR-589-3p | -0.128 | 0.890 | 7.461E-01 | Neerincx et al. |
| hsa-miR-1185-5p | -0.157 | 1.569 | 7.461E-01 | Neerincx et al. |
| hsa-miR-196b-5p | 0.143 | 8.204 | 7.461E-01 | Neerincx et al. |
| hsa-miR-29b-3p | 0.116 | 7.052 | 7.465E-01 | Neerincx et al. |
| hsa-miR-146b-5p | -0.152 | 11.565 | 7.614E-01 | Neerincx et al. |
| hsa-miR-199a-5p | -0.101 | 8.819 | 7.760E-01 | Neerincx et al. |
| hsa-miR-100-5p | -0.152 | 9.534 | 7.760E-01 | Neerincx et al. |
| hsa-miR-582-3p | -0.129 | 5.173 | 7.760E-01 | Neerincx et al. |
| hsa-miR-219a-1-3p | 0.114 | 0.188 | 7.760E-01 | Neerincx et al. |
| hsa-miR-652-5p | -0.093 | 2.311 | 7.760E-01 | Neerincx et al. |
| hsa-miR-103a-3p | 0.054 | 10.962 | 7.803E-01 | Neerincx et al. |
| hsa-miR-505-3p | 0.066 | 4.385 | 7.847E-01 | Neerincx et al. |
| hsa-miR-185-5p | -0.071 | 3.634 | 7.928E-01 | Neerincx et al. |
| hsa-miR-203a-5p | -0.123 | 0.631 | 8.003E-01 | Neerincx et al. |
| hsa-miR-196b-3p | 0.103 | 2.334 | 8.054E-01 | Neerincx et al. |
| hsa-miR-148a-5p | 0.097 | 7.980 | 8.060E-01 | Neerincx et al. |
| hsa-miR-2277-5p | 0.103 | 0.785 | 8.131E-01 | Neerincx et al. |
| hsa-miR-574-5p | -0.195 | 3.282 | 8.210E-01 | Neerincx et al. |
| hsa-miR-889-3p | -0.073 | 4.415 | 8.210E-01 | Neerincx et al. |
| hsa-miR-151b | 0.067 | 6.782 | 8.210E-01 | Neerincx et al. |
| hsa-miR-3605-3p | -0.074 | 1.642 | 8.210E-01 | Neerincx et al. |
| hsa-miR-210-3p | -0.092 | 7.173 | 8.210E-01 | Neerincx et al. |
| hsa-miR-219a-5p | -0.097 | 0.170 | 8.220E-01 | Neerincx et al. |
| hsa-miR-3605-5p | 0.098 | 0.597 | 8.263E-01 | Neerincx et al. |
| hsa-miR-323a-3p | -0.086 | 1.684 | 8.263E-01 | Neerincx et al. |
| hsa-miR-374b-3p | 0.062 | 3.204 | 8.394E-01 | Neerincx et al. |
| hsa-miR-191-5p | -0.087 | 13.776 | 8.414E-01 | Neerincx et al. |
| hsa-miR-423-3p | 0.045 | 9.579 | 8.425E-01 | Neerincx et al. |
| hsa-miR-148a-3p | 0.080 | 14.564 | 8.492E-01 | Neerincx et al. |
| hsa-miR-625-5p | 0.072 | 3.140 | 8.492E-01 | Neerincx et al. |
| hsa-miR-505-5p | 0.078 | -0.063 | 8.553E-01 | Neerincx et al. |
| hsa-miR-3656 | -0.139 | 1.756 | 8.687E-01 | Neerincx et al. |
| hsa-miR-330-5p | 0.053 | 3.857 | 8.705E-01 | Neerincx et al. |
| hsa-miR-193a-5p | -0.066 | 5.423 | 8.749E-01 | Neerincx et al. |
| hsa-miR-34b-5p | 0.072 | 0.588 | 8.783E-01 | Neerincx et al. |
| hsa-miR-125a-3p | -0.053 | 2.318 | 8.783E-01 | Neerincx et al. |
| hsa-miR-410-3p | 0.042 | 5.870 | 8.805E-01 | Neerincx et al. |
| hsa-miR-146a-5p | 0.088 | 9.652 | 8.870E-01 | Neerincx et al. |
| hsa-miR-335-5p | 0.047 | 6.365 | 8.964E-01 | Neerincx et al. |
| hsa-miR-1303 | 0.099 | 0.061 | 8.964E-01 | Neerincx et al. |
| hsa-miR-29a-3p | 0.035 | 10.584 | 8.964E-01 | Neerincx et al. |
| hsa-miR-1271-5p | 0.098 | 1.225 | 8.964E-01 | Neerincx et al. |
| hsa-miR-379-5p | 0.049 | 2.365 | 8.964E-01 | Neerincx et al. |
| hsa-miR-485-3p | 0.040 | 1.488 | 8.964E-01 | Neerincx et al. |
| hsa-miR-874-5p | -0.051 | 2.313 | 8.964E-01 | Neerincx et al. |
| hsa-miR-331-3p | -0.045 | 4.628 | 8.969E-01 | Neerincx et al. |
| hsa-miR-433-3p | 0.043 | 1.207 | 8.970E-01 | Neerincx et al. |
| hsa-let-7a-3p | -0.040 | 4.249 | 9.015E-01 | Neerincx et al. |
| hsa-miR-1246 | 0.128 | 2.927 | 9.030E-01 | Neerincx et al. |
| hsa-miR-15b-5p | 0.033 | 7.361 | 9.030E-01 | Neerincx et al. |
| hsa-miR-148b-5p | -0.037 | 5.405 | 9.030E-01 | Neerincx et al. |
| hsa-miR-33a-5p | 0.047 | 4.089 | 9.030E-01 | Neerincx et al. |
| hsa-miR-6842-3p | -0.041 | 0.639 | 9.034E-01 | Neerincx et al. |
| hsa-miR-4443 | 0.047 | 3.189 | 9.066E-01 | Neerincx et al. |
| hsa-miR-23a-5p | 0.058 | 2.632 | 9.172E-01 | Neerincx et al. |
| hsa-miR-432-5p | -0.030 | 2.663 | 9.270E-01 | Neerincx et al. |
| hsa-miR-500a-3p | 0.028 | 7.846 | 9.346E-01 | Neerincx et al. |
| hsa-miR-203b-3p | -0.037 | 2.510 | 9.346E-01 | Neerincx et al. |
| hsa-let-7c-5p | 0.034 | 8.679 | 9.417E-01 | Neerincx et al. |
| hsa-miR-191-3p | -0.025 | 1.787 | 9.555E-01 | Neerincx et al. |
| hsa-miR-100-3p | -0.029 | 1.703 | 9.575E-01 | Neerincx et al. |
| hsa-miR-4492 | -0.040 | 1.664 | 9.618E-01 | Neerincx et al. |
| hsa-miR-4677-3p | -0.012 | 3.093 | 9.725E-01 | Neerincx et al. |
| hsa-miR-99b-5p | 0.022 | 11.081 | 9.738E-01 | Neerincx et al. |
| hsa-miR-654-3p | -0.011 | 6.554 | 9.738E-01 | Neerincx et al. |
| hsa-miR-299-5p | 0.012 | 0.533 | 9.738E-01 | Neerincx et al. |
| hsa-let-7f-1-3p | -0.009 | 1.414 | 9.738E-01 | Neerincx et al. |
| hsa-miR-193b-5p | -0.014 | 0.178 | 9.749E-01 | Neerincx et al. |
| hsa-miR-193a-3p | 0.019 | 4.828 | 9.749E-01 | Neerincx et al. |
| hsa-miR-5091 | -0.010 | 0.731 | 9.772E-01 | Neerincx et al. |
| hsa-miR-641 | -0.009 | 2.138 | 9.772E-01 | Neerincx et al. |
| hsa-miR-151a-5p | -0.006 | 11.047 | 9.811E-01 | Neerincx et al. |
| hsa-miR-362-5p | -0.008 | 4.859 | 9.841E-01 | Neerincx et al. |
| hsa-miR-625-3p | 0.004 | 2.733 | 9.897E-01 | Neerincx et al. |
| hsa-miR-99a-5p | -0.005 | 6.270 | 9.897E-01 | Neerincx et al. |

| **Table S3:** Differentially expressed miRNAs between left and right normal and tumor colon tissue in our data set, Neerincx et al. and TCGA. Shown are miRNAs that were significant in at least one of the datasets within either normal tissue or tumor tissue. The statistical comparison used was Right-Left such that a positive "Fold Change" indicates that the corresponding miRNA is up-regulated in right compared to left. "miRNA" lists the canonical mature miRNA name (from miRBase 21.0); "Fold Change (Log2)" is the log2 fold change of the Right-Left statistical comparison (corresponding to the log2 of the isomiR's average expression in right subtracted by its average expression in left values computed by limma); "Average Expression" is the miRNAs average log2 cpm expression in the dataset (as computed by limma); "P-value" is the non-adjusted p-value. "Adjusted P-value" is the Benjamini-Hochberg adjusted p-value. "Comparison" indicates the statistical comparison which is Right vs left in either normal tissue or tumor tissue. | | | | | | |
| --- | --- | --- | --- | --- | --- | --- |
| miRNA | Fold Change (Log2) | Average Expression | P-value | Adjusted P-value | Dataset | Comparison |
| hsa-miR-196b-5p | 1.08 | 8.56 | 2.28E-07 | 4.77E-04 | Mjelle et al. | Right vs Left  Normal tissue |
| hsa-miR-375 | -1.16 | 12.71 | 5.53E-06 | 5.78E-03 | Mjelle et al. | Right vs Left  Normal tissue |
| hsa-miR-196b-3p | 0.98 | 2.29 | 1.03E-05 | 7.17E-03 | Mjelle et al. | Right vs Left  Normal tissue |
| hsa-miR-615-3p | 2.80 | 0.05 | 2.15E-05 | 1.12E-02 | Mjelle et al. | Right vs Left  Normal tissue |
| hsa-miR-450b-5p | 0.98 | 3.38 | 3.54E-05 | 1.48E-02 | Mjelle et al. | Right vs Left  Normal tissue |
| hsa-miR-10b-3p | 0.71 | 3.61 | 7.08E-05 | 2.19E-02 | Mjelle et al. | Right vs Left  Normal tissue |
| hsa-miR-10b-5p | 0.54 | 16.82 | 7.62E-05 | 2.19E-02 | Mjelle et al. | Right vs Left  Normal tissue |
| hsa-miR-561-5p | 0.95 | 3.75 | 8.37E-05 | 2.19E-02 | Mjelle et al. | Right vs Left  Normal tissue |
| hsa-miR-542-3p | 0.94 | 2.76 | 1.19E-04 | 2.77E-02 | Mjelle et al. | Right vs Left  Normal tissue |
| hsa-miR-450a-5p | 0.99 | 2.71 | 2.18E-04 | 4.56E-02 | Mjelle et al. | Right vs Left  Normal tissue |
| hsa-miR-490-5p | 1.08 | -2.09 | 4.34E-02 | 7.52E-01 | Mjelle et al. | Right vs Left  Normal tissue |
| hsa-miR-142-3p | 0.09 | 8.10 | 6.70E-01 | 8.54E-01 | Mjelle et al. | Right vs Left  Normal tissue |
| hsa-miR-490-3p | 0.15 | 1.12 | 8.10E-01 | 9.14E-01 | Mjelle et al. | Right vs Left  Normal tissue |
| hsa-miR-615-3p | 3.47 | -0.27 | 7.70E-07 | 1.70E-03 | Neerincx et al. | Right vs Left  Normal tissue |
| hsa-miR-561-5p | 1.69 | 3.72 | 2.55E-06 | 2.82E-03 | Neerincx et al. | Right vs Left  Normal tissue |
| hsa-miR-490-3p | -3.50 | 1.87 | 4.44E-06 | 3.27E-03 | Neerincx et al. | Right vs Left  Normal tissue |
| hsa-miR-490-5p | -3.18 | -1.30 | 1.42E-05 | 7.57E-03 | Neerincx et al. | Right vs Left  Normal tissue |
| hsa-miR-196b-5p | 1.15 | 8.03 | 1.71E-05 | 7.57E-03 | Neerincx et al. | Right vs Left  Normal tissue |
| hsa-miR-142-3p | 1.25 | 8.05 | 2.78E-05 | 1.03E-02 | Neerincx et al. | Right vs Left  Normal tissue |
| hsa-miR-450b-5p | 1.21 | 3.59 | 6.17E-04 | 1.71E-01 | Neerincx et al. | Right vs Left  Normal tissue |
| hsa-miR-542-3p | 1.35 | 3.04 | 7.64E-04 | 1.88E-01 | Neerincx et al. | Right vs Left  Normal tissue |
| hsa-miR-196b-3p | 1.11 | 2.15 | 1.21E-03 | 2.06E-01 | Neerincx et al. | Right vs Left  Normal tissue |
| hsa-miR-450a-5p | 1.05 | 2.77 | 4.17E-03 | 4.45E-01 | Neerincx et al. | Right vs Left  Normal tissue |
| hsa-miR-375 | -0.63 | 13.52 | 3.31E-02 | 6.82E-01 | Neerincx et al. | Right vs Left  Normal tissue |
| hsa-miR-10b-3p | 0.42 | 3.34 | 2.22E-01 | 6.82E-01 | Neerincx et al. | Right vs Left  Normal tissue |
| hsa-miR-10b-5p | 0.24 | 15.93 | 5.54E-01 | 8.14E-01 | Neerincx et al. | Right vs Left  Normal tissue |
| hsa-miR-615-3p | 4.48 | 1.78 | 5.12E-08 | 1.07E-04 | Mjelle et al. | Right vs Left  Tumor tissue |
| hsa-miR-20a-3p | -0.95 | 2.17 | 4.15E-04 | 8.76E-02 | Mjelle et al. | Right vs Left  Tumor tissue |
| hsa-miR-362-5p | -1.03 | 5.70 | 5.82E-04 | 1.01E-01 | Mjelle et al. | Right vs Left  Tumor tissue |
| hsa-miR-19b-3p | -0.75 | 9.93 | 1.00E-03 | 1.33E-01 | Mjelle et al. | Right vs Left  Tumor tissue |
| hsa-miR-625-5p | 1.06 | 3.91 | 1.15E-03 | 1.41E-01 | Mjelle et al. | Right vs Left  Tumor tissue |
| hsa-miR-146a-5p | 0.98 | 10.53 | 2.09E-03 | 1.83E-01 | Mjelle et al. | Right vs Left  Tumor tissue |
| hsa-miR-92b-3p | 0.77 | 9.89 | 2.21E-03 | 1.84E-01 | Mjelle et al. | Right vs Left  Tumor tissue |
| hsa-miR-106a-5p | -0.82 | 5.44 | 7.84E-03 | 4.04E-01 | Mjelle et al. | Right vs Left  Tumor tissue |
| hsa-miR-625-3p | 0.88 | 3.50 | 8.21E-03 | 4.04E-01 | Mjelle et al. | Right vs Left  Tumor tissue |
| hsa-miR-10a-5p | 0.66 | 16.93 | 1.94E-02 | 6.35E-01 | Mjelle et al. | Right vs Left  Tumor tissue |
| hsa-miR-146b-5p | 0.48 | 11.93 | 2.71E-02 | 7.53E-01 | Mjelle et al. | Right vs Left  Tumor tissue |
| hsa-miR-19a-3p | -0.48 | 8.01 | 3.12E-02 | 8.03E-01 | Mjelle et al. | Right vs Left  Tumor tissue |
| hsa-miR-188-5p | -0.55 | 3.42 | 3.30E-02 | 8.16E-01 | Mjelle et al. | Right vs Left  Tumor tissue |
| hsa-miR-196b-5p | -0.73 | 8.40 | 7.35E-02 | 9.99E-01 | Mjelle et al. | Right vs Left  Tumor tissue |
| hsa-miR-10b-5p | 0.47 | 15.79 | 8.27E-02 | 9.99E-01 | Mjelle et al. | Right vs Left  Tumor tissue |
| hsa-miR-155-5p | 0.51 | 8.68 | 8.57E-02 | 9.99E-01 | Mjelle et al. | Right vs Left  Tumor tissue |
| hsa-miR-605-5p | -0.79 | -1.58 | 9.61E-02 | 9.99E-01 | Mjelle et al. | Right vs Left  Tumor tissue |
| hsa-miR-4446-3p | 0.81 | -0.28 | 1.16E-01 | 9.99E-01 | Mjelle et al. | Right vs Left  Tumor tissue |
| hsa-miR-455-3p | -0.45 | 3.78 | 1.70E-01 | 9.99E-01 | Mjelle et al. | Right vs Left  Tumor tissue |
| hsa-miR-146b-3p | 0.39 | 5.28 | 1.81E-01 | 9.99E-01 | Mjelle et al. | Right vs Left  Tumor tissue |
| hsa-miR-1275 | -0.68 | 2.95 | 2.12E-01 | 9.99E-01 | Mjelle et al. | Right vs Left  Tumor tissue |
| hsa-miR-6761-5p | -0.46 | -1.76 | 2.42E-01 | 9.99E-01 | Mjelle et al. | Right vs Left  Tumor tissue |
| hsa-miR-10b-3p | 0.31 | 2.83 | 2.95E-01 | 9.99E-01 | Mjelle et al. | Right vs Left  Tumor tissue |
| hsa-miR-182-5p | -0.29 | 12.93 | 3.34E-01 | 9.99E-01 | Mjelle et al. | Right vs Left  Tumor tissue |
| hsa-miR-155-3p | -0.43 | -1.82 | 3.42E-01 | 9.99E-01 | Mjelle et al. | Right vs Left  Tumor tissue |
| hsa-miR-342-3p | 0.23 | 7.93 | 4.06E-01 | 9.99E-01 | Mjelle et al. | Right vs Left  Tumor tissue |
| hsa-miR-33a-5p | -0.21 | 3.97 | 4.76E-01 | 9.99E-01 | Mjelle et al. | Right vs Left  Tumor tissue |
| hsa-miR-143-5p | -0.22 | 3.06 | 4.87E-01 | 9.99E-01 | Mjelle et al. | Right vs Left  Tumor tissue |
| hsa-miR-409-5p | -0.15 | 0.73 | 6.73E-01 | 9.99E-01 | Mjelle et al. | Right vs Left  Tumor tissue |
| hsa-miR-323b-3p | -0.18 | 0.70 | 6.76E-01 | 9.99E-01 | Mjelle et al. | Right vs Left  Tumor tissue |
| hsa-miR-127-5p | -0.10 | 2.67 | 7.61E-01 | 9.99E-01 | Mjelle et al. | Right vs Left  Tumor tissue |
| hsa-miR-188-3p | -0.05 | 0.80 | 8.98E-01 | 9.99E-01 | Mjelle et al. | Right vs Left  Tumor tissue |
| hsa-miR-3170 | -0.01 | -2.58 | 9.76E-01 | 9.99E-01 | Mjelle et al. | Right vs Left  Tumor tissue |
| hsa-miR-107 | 0.00 | 8.49 | 9.81E-01 | 9.99E-01 | Mjelle et al. | Right vs Left  Tumor tissue |
| hsa-miR-615-3p | 1.94 | 0.17 | 5.04E-26 | 1.15E-22 | TCGA | Right vs Left  Tumor tissue |
| hsa-miR-10b-5p | 0.88 | 15.88 | 1.11E-18 | 1.26E-15 | TCGA | Right vs Left  Tumor tissue |
| hsa-miR-10b-3p | 1.04 | 4.17 | 1.81E-16 | 1.37E-13 | TCGA | Right vs Left  Tumor tissue |
| hsa-miR-155-5p | 0.79 | 8.91 | 9.58E-12 | 5.45E-09 | TCGA | Right vs Left  Tumor tissue |
| hsa-miR-625-3p | 0.70 | 8.42 | 1.74E-10 | 7.90E-08 | TCGA | Right vs Left  Tumor tissue |
| hsa-miR-625-5p | 0.68 | 8.41 | 3.81E-10 | 1.45E-07 | TCGA | Right vs Left  Tumor tissue |
| hsa-miR-146a-5p | 0.68 | 8.37 | 1.32E-08 | 4.30E-06 | TCGA | Right vs Left  Tumor tissue |
| hsa-miR-92b-3p | 0.50 | 6.62 | 5.53E-07 | 1.57E-04 | TCGA | Right vs Left  Tumor tissue |
| hsa-miR-6761-5p | -0.62 | 1.64 | 8.36E-07 | 2.11E-04 | TCGA | Right vs Left  Tumor tissue |
| hsa-miR-20a-3p | -0.62 | 5.03 | 1.08E-06 | 2.45E-04 | TCGA | Right vs Left  Tumor tissue |
| hsa-miR-4446-3p | 0.59 | -0.68 | 3.03E-06 | 6.27E-04 | TCGA | Right vs Left  Tumor tissue |
| hsa-miR-1275 | -0.79 | 2.19 | 7.30E-06 | 1.38E-03 | TCGA | Right vs Left  Tumor tissue |
| hsa-miR-342-3p | 0.48 | 6.68 | 8.20E-06 | 1.40E-03 | TCGA | Right vs Left  Tumor tissue |
| hsa-miR-155-3p | 0.76 | 0.05 | 8.62E-06 | 1.40E-03 | TCGA | Right vs Left  Tumor tissue |
| hsa-miR-146b-3p | 0.51 | 6.37 | 1.07E-05 | 1.58E-03 | TCGA | Right vs Left  Tumor tissue |
| hsa-miR-10a-5p | 0.67 | 3.03 | 1.11E-05 | 1.58E-03 | TCGA | Right vs Left  Tumor tissue |
| hsa-miR-127-5p | -0.39 | 8.54 | 1.41E-05 | 1.88E-03 | TCGA | Right vs Left  Tumor tissue |
| hsa-miR-196b-5p | -0.80 | 0.01 | 1.67E-05 | 2.06E-03 | TCGA | Right vs Left  Tumor tissue |
| hsa-miR-455-3p | -0.51 | 8.34 | 1.72E-05 | 2.06E-03 | TCGA | Right vs Left  Tumor tissue |
| hsa-miR-146b-5p | 0.42 | 9.58 | 2.15E-05 | 2.45E-03 | TCGA | Right vs Left  Tumor tissue |
| hsa-miR-19b-3p | -0.42 | 7.64 | 2.37E-05 | 2.57E-03 | TCGA | Right vs Left  Tumor tissue |
| hsa-miR-106a-5p | -0.74 | 4.37 | 5.65E-05 | 5.81E-03 | TCGA | Right vs Left  Tumor tissue |
| hsa-miR-605-5p | -0.50 | 0.03 | 5.87E-05 | 5.81E-03 | TCGA | Right vs Left  Tumor tissue |
| hsa-miR-33a-5p | -0.56 | 6.06 | 1.26E-04 | 1.16E-02 | TCGA | Right vs Left  Tumor tissue |
| hsa-miR-3170 | -0.53 | 1.73 | 1.27E-04 | 1.16E-02 | TCGA | Right vs Left  Tumor tissue |
| hsa-miR-19a-3p | -0.50 | 6.51 | 1.54E-04 | 1.35E-02 | TCGA | Right vs Left  Tumor tissue |
| hsa-miR-362-5p | -0.48 | 4.86 | 2.28E-04 | 1.93E-02 | TCGA | Right vs Left  Tumor tissue |
| hsa-miR-107 | 0.30 | 4.66 | 2.53E-04 | 2.06E-02 | TCGA | Right vs Left  Tumor tissue |
| hsa-miR-409-5p | -0.39 | 4.01 | 3.65E-04 | 2.82E-02 | TCGA | Right vs Left  Tumor tissue |
| hsa-miR-323b-3p | -0.51 | 2.97 | 3.71E-04 | 2.82E-02 | TCGA | Right vs Left  Tumor tissue |
| hsa-miR-188-3p | -0.54 | 1.46 | 4.66E-04 | 3.42E-02 | TCGA | Right vs Left  Tumor tissue |
| hsa-miR-188-5p | -0.40 | 2.99 | 5.44E-04 | 3.87E-02 | TCGA | Right vs Left  Tumor tissue |
| hsa-miR-143-5p | -0.55 | 7.60 | 6.05E-04 | 4.17E-02 | TCGA | Right vs Left  Tumor tissue |
| hsa-miR-182-5p | 0.08 | 5.20 | 6.04E-01 | 1.00E+00 | TCGA | Right vs Left  Tumor tissue |
| hsa-miR-615-3p | 4.26 | 0.71 | 2.84E-08 | 6.29E-05 | Neerincx et al. | Right vs Left  Tumor tissue |
| hsa-miR-182-5p | 1.56 | 12.97 | 3.09E-05 | 3.42E-02 | Neerincx et al. | Right vs Left  Tumor tissue |
| hsa-miR-10b-5p | 1.62 | 15.21 | 8.10E-04 | 5.98E-01 | Neerincx et al. | Right vs Left  Tumor tissue |
| hsa-miR-10b-3p | 1.07 | 2.11 | 1.68E-02 | 7.74E-01 | Neerincx et al. | Right vs Left  Tumor tissue |
| hsa-miR-146a-5p | 1.16 | 9.71 | 2.67E-02 | 7.74E-01 | Neerincx et al. | Right vs Left  Tumor tissue |
| hsa-miR-10a-5p | 0.61 | 16.97 | 1.00E-01 | 7.74E-01 | Neerincx et al. | Right vs Left  Tumor tissue |
| hsa-miR-146b-5p | 0.68 | 11.59 | 1.07E-01 | 7.74E-01 | Neerincx et al. | Right vs Left  Tumor tissue |
| hsa-miR-1275 | -0.85 | 4.28 | 1.29E-01 | 7.74E-01 | Neerincx et al. | Right vs Left  Tumor tissue |
| hsa-miR-455-3p | -0.47 | 4.14 | 2.72E-01 | 7.74E-01 | Neerincx et al. | Right vs Left  Tumor tissue |
| hsa-miR-6761-5p | -0.44 | -1.52 | 2.83E-01 | 7.74E-01 | Neerincx et al. | Right vs Left  Tumor tissue |
| hsa-miR-92b-3p | 0.34 | 10.01 | 3.35E-01 | 7.74E-01 | Neerincx et al. | Right vs Left  Tumor tissue |
| hsa-miR-342-3p | -0.34 | 6.93 | 3.42E-01 | 7.74E-01 | Neerincx et al. | Right vs Left  Tumor tissue |
| hsa-miR-155-3p | -0.31 | -2.69 | 3.46E-01 | 7.74E-01 | Neerincx et al. | Right vs Left  Tumor tissue |
| hsa-miR-4446-3p | -0.53 | -1.12 | 3.61E-01 | 7.74E-01 | Neerincx et al. | Right vs Left  Tumor tissue |
| hsa-miR-19a-3p | 0.35 | 7.05 | 4.26E-01 | 7.74E-01 | Neerincx et al. | Right vs Left  Tumor tissue |
| hsa-miR-127-5p | -0.31 | 3.00 | 4.35E-01 | 7.80E-01 | Neerincx et al. | Right vs Left  Tumor tissue |
| hsa-miR-625-3p | 0.34 | 2.66 | 4.66E-01 | 8.00E-01 | Neerincx et al. | Right vs Left  Tumor tissue |
| hsa-miR-323b-3p | -0.23 | 1.10 | 5.26E-01 | 8.35E-01 | Neerincx et al. | Right vs Left  Tumor tissue |
| hsa-miR-146b-3p | 0.17 | 5.57 | 6.08E-01 | 8.53E-01 | Neerincx et al. | Right vs Left  Tumor tissue |
| hsa-miR-19b-3p | 0.19 | 9.27 | 6.20E-01 | 8.58E-01 | Neerincx et al. | Right vs Left  Tumor tissue |
| hsa-miR-625-5p | 0.17 | 3.24 | 6.82E-01 | 8.88E-01 | Neerincx et al. | Right vs Left  Tumor tissue |
| hsa-miR-155-5p | 0.13 | 7.49 | 6.89E-01 | 8.92E-01 | Neerincx et al. | Right vs Left  Tumor tissue |
| hsa-miR-143-5p | -0.15 | 3.21 | 7.20E-01 | 8.92E-01 | Neerincx et al. | Right vs Left  Tumor tissue |
| hsa-miR-106a-5p | -0.18 | 2.88 | 7.32E-01 | 8.95E-01 | Neerincx et al. | Right vs Left  Tumor tissue |
| hsa-miR-409-5p | -0.19 | 1.21 | 7.36E-01 | 8.97E-01 | Neerincx et al. | Right vs Left  Tumor tissue |
| hsa-miR-107 | 0.08 | 8.96 | 7.91E-01 | 9.23E-01 | Neerincx et al. | Right vs Left  Tumor tissue |
| hsa-miR-3170 | -0.08 | -2.60 | 8.14E-01 | 9.35E-01 | Neerincx et al. | Right vs Left  Tumor tissue |
| hsa-miR-362-5p | -0.10 | 5.27 | 8.14E-01 | 9.35E-01 | Neerincx et al. | Right vs Left  Tumor tissue |
| hsa-miR-196b-5p | 0.10 | 8.23 | 8.30E-01 | 9.38E-01 | Neerincx et al. | Right vs Left  Tumor tissue |
| hsa-miR-33a-5p | -0.08 | 4.06 | 8.66E-01 | 9.55E-01 | Neerincx et al. | Right vs Left  Tumor tissue |
| hsa-miR-188-3p | 0.07 | 0.47 | 8.70E-01 | 9.56E-01 | Neerincx et al. | Right vs Left  Tumor tissue |
| hsa-miR-605-5p | 0.05 | -1.82 | 9.15E-01 | 9.70E-01 | Neerincx et al. | Right vs Left  Tumor tissue |
| hsa-miR-188-5p | -0.05 | 3.01 | 9.17E-01 | 9.72E-01 | Neerincx et al. | Right vs Left  Tumor tissue |
| hsa-miR-20a-3p | -0.02 | 1.83 | 9.68E-01 | 9.87E-01 | Neerincx et al. | Right vs Left  Tumor tissue |

| **Table S4.** Differentially expressed miRNAs between MSS and MSI tumors in our dataset and TCGA. Shown is miRNAs that were significant in at least one of the datasets. The statistical comparison used was MSS-MSI such that a positive "Fold Change" indicates that the corresponding miRNA is up-regulated in MSS compared to MSI. "miRNA" lists the canonical mature miRNA name (from miRBase 21.0); "Fold Change (Log2)" is the log2 fold change of the MSS-MSI statistical comparison (corresponding to the log2 of the isomiR's average expression in MSS subtracted by its average expression in MSI values computed by limma); "Average Expression" is the miRNAs average log2 cpm expression in the dataset (as computed by limma); "P-value" is the non-adjusted p-value. "Adjusted P-value" is the Benjamini-Hochberg adjusted p-value. | | | | | |
| --- | --- | --- | --- | --- | --- |
| miRNA | Fold Change (Log2) | Average Expression | P-value | Adjusted P-value | Dataset |
| hsa-miR-7641 | -2.35 | 3.87 | 4.22E-06 | 8.82E-03 | Mjelle et al. |
| hsa-miR-335-5p | 0.77 | 6.92 | 5.99E-05 | 4.10E-02 | Mjelle et al. |
| hsa-miR-26a-5p | 0.75 | 15.27 | 7.73E-05 | 4.10E-02 | Mjelle et al. |
| hsa-miR-6087 | -1.54 | 7.60 | 2.79E-02 | 5.13E-01 | Mjelle et al. |
| hsa-miR-625-3p | -0.59 | 3.50 | 3.96E-02 | 5.89E-01 | Mjelle et al. |
| hsa-miR-155-5p | -0.48 | 8.68 | 6.31E-02 | 7.16E-01 | Mjelle et al. |
| hsa-miR-92b-3p | -0.31 | 9.89 | 1.53E-01 | 9.73E-01 | Mjelle et al. |
| hsa-miR-625-5p | -0.33 | 3.91 | 2.33E-01 | 9.83E-01 | Mjelle et al. |
| hsa-miR-21-3p | -0.18 | 11.92 | 3.42E-01 | 9.83E-01 | Mjelle et al. |
| hsa-miR-92b-3p | -1.46 | 6.76 | 1.80E-07 | 3.16E-05 | TCGA |
| hsa-miR-625-5p | -1.53 | 8.30 | 3.36E-06 | 2.68E-04 | TCGA |
| hsa-miR-625-3p | -1.55 | 8.31 | 4.60E-06 | 2.68E-04 | TCGA |
| hsa-miR-21-3p | -0.98 | 12.31 | 1.38E-05 | 6.03E-04 | TCGA |
| hsa-miR-155-5p | -1.02 | 8.94 | 2.88E-04 | 1.01E-02 | TCGA |
| hsa-miR-6087 | -1.57 | 6.20 | 1.60E-03 | 4.67E-02 | TCGA |
| hsa-miR-335-5p | 0.47 | 4.80 | 1.17E-01 | 3.13E-01 | TCGA |
| hsa-miR-26a-5p | 0.20 | 10.52 | 2.59E-01 | 5.04E-01 | TCGA |
