# Supplementary material for: Small RNA expression from viruses, bacteria and human miRNAs in colon cancer tissue and its association with microsatellite instability and tumor location
Source: BMC Cancer. 2019 Feb 20;19:161. doi: 10.1186/s12885-019-5330-0 (PMC6381638; doi:10.1186/s12885-019-5330-0)
Supplement: Supplementary file 2 — Contains sequencing statistics results and additional methods description. (DOCX 49 kb) [file 12885_2019_5330_MOESM2_ESM.docx]

## Sequencing statistics

On average, 11 mill. reads aligned to the human genome per sample (Additional File 3: Figure S1A). To determine the different RNA classes present in the samples we annotated the sequences to the miRNA database miRBase v21[1] and the RNA Central database of ncRNAs (<http://rnacentral.org>). The annotations revealed that most of the reads that aligned to the human genome mapped to either of the two databases (Additional File 3: Figure S1A). Further subdividing the RNAs into gene classes showed that the sequencing libraries were dominated by miRNAs followed by transfer RNAs (tRNAs), long non-coding RNAs (lncRNAs), ribosomal RNAs (rRNAs), as well as other ncRNAs (Additional File 3: Figure S1A-B). Four samples (S25, S26, S47 and S48 in Additional File 3: Figure S1A-B) had very few reads aligning to the human genome (average 1.3%). The majority (88%) of the unaligned reads in these samples mapped to the calibrator RNAs added to each sample prior to library construction (see methods and Additional File 3: Figure S1C), indicating technical issues with RNA isolation or library preparation. These samples were therefore removed from further sequence analysis.

## MicroRNA cluster definition

MicroRNA and snoRNA that is located within 10 000 nts were defined as being located within the same genomic cluster[2].

## Clustered miRNAs tend to be co-expressed

To investigate whether altered miRNA transcription could explain changes in miRNA expression, we analyzed pre-miRNAs that map to genomic clusters, since such clustered pre-miRNAs are likely to be processed from a common primary miRNA (pri-miRNA) transcript[2]. We detected eight clusters containing two or more significant miRNAs (Additional File 4: Figure S2A). Of these, five clusters (ID4, ID6, ID8, ID14, ID15) showed common expression of all miRNAs in the cluster, whereas three clusters contained miRNAs that were both up- and downregulated in tumor vs normal tissue. For example, the oncogenic miR-17-92 cluster (ID6) comprised eight significant miRNAs, all of which were upregulated in tumor vs normal tissue. In comparison, cluster ID3 contained seven significant miRNAs, of which four were downregulated and three were upregulated in tumor vs normal. Thus, altered transcription seems to explain some, but not all the changes in miRNA expression.

Some of the miRNA clusters have previously been associated with cancer. The cluster with most significant miRNAs, the miR-154 cluster (ID2), has been identified as a tumor suppressor locus that is dysregulated in many cancers. Some members of the miR-17-92 (ID6) and miR-144/451a (ID14) clusters are differentially expressed in CRC tumor tissue[3, 4] and the miR-17-92 cluster is among the most frequently altered miRNA cluster in cancer[5]. Epigenetic regulation of miRNA clusters has been observed in CRC and can explain changes in miRNA expression in tumor and normal tissue[6, 7].

Although different transcriptional programs can explain some of the changes in miRNA expression between tumor and normal tissue, changes in single miRNAs hint towards additional downstream regulatory mechanisms. Some clustered miRNAs show opposite expression (e.g. cluster ID3; Additional File 4: Figure S2A), indicating sequence specific regulation that could be related to preferences in the miRNA maturation process.

## Clustered sncRNAs

To determine whether the distinct dysregulation of the RNA classes could be due to common transcriptional changes, we mapped the genomic location of the significant RNAs. We used the same cluster definition as for miRNAs (see Experimental Procedures) and found that snoRNAs but no other ncRNA classes were often located in clusters. We detected 13 unique snoRNA clusters, whereby three clusters hosted most of the snoRNAs (Additional File 5: Figure S3B). For the clusters that contained more than two snoRNAs, we detected common regulation for all snoRNAs, indicating that that these snoRNAs are dysregulated due to transcriptional changes. A large number of snoRNAs were not located in genomic clusters, and here we did not observe any common regulation (Additional File 5: Figure S3C). Interestingly, miRNA clusters ID2 and ID3 and snoRNA-clusters ID12 and ID13 are all located on chromosome 14, in close proximity to the *MEG3* and *MEG8* genes (Additional File 5: Figure S3D).

## Processing of sequence data

## FastQC was used for quality control of the raw sequence data[8]. Trimming of sequence adapters from the 3’end of the raw sequences was performed by using cutadapt-1.2.1[9]. The cut sequences were collapsed with the fastx collapser tool into single unique reads along with their total read count and mapped to the human (hg38) genome using bowtie2, allowing for up to 10 alignments per read to account for reads from duplicated miRNA loci (bowtie2 – k10). Reads overlapping with mature miRNA loci were identified using htseq-count from the HTseq python package[10]. These reads were further filtered to identify those with perfect alignment to the genome, and the total read count for mature miRNAs were then computed by summing the total read count per sequence (isomiR) overlapping each mature miRNA locus. Mature miRNAs and non-coding RNAs were annotated using miRBase (Release 21, 2014)[11] and RNA Central (<http://rnacentral.org>) respectively. IsomiR variants were detected using SeqBuster[12] combined with a panel of in-house perl and R-scripts, available upon request. IsomiRs with mismatches to the genome were discarded from the analysis, as these could not be excluded as sequencing errors. However, isomiRs with non-templated addition at the 3’end were included in the analysis. Virus miRNAs and bacterial small RNAs were detected by first mapping the raw reads to the human genome, then using the reads that did not map to the human genome to map against the viral miRNAs in miRBase and the Ensembl bacteria genomes (<http://bacteria.ensembl.org/index.html>). Viral miRNAs were counted using SeqBuster and the miRBase virus reference. We used the canonical human miRNA library sizes and calcNormFactors from the DGEList-object to normalize the expression of virus miRNAs. The expression of *F.nucleatum* was normalized using counts per million within the bacteria matrix.

## Differential expression analysis and statistics

Differentially expressed miRNAs and isomiRs were identified using the Bioconductor package limma combined with voom transformation[13, 14]. To compare miRNA expression between samples, read counts were normalized using counts per million (cpm) normalization. For miRNAs, ncRNAs and isomiRs, we required expression of 1 cpm in at least 50% of the samples when comparing tumor vs normal. When analyzing tumor location, no expression filtering was performed due to the binary expression profile of miR-615 that would otherwise be missed.

All correlations analysis throughout the manuscript were calculated using Pearson correlation in R. The p-values in figure 4A-B were calculated from an ANOVA on a linear mixed-effects model using the *lmer* R package. The analysis considers age, sex and pairing of the subjects and uses the logFC values as input. The P-values in Figure 4C were calculated using a two-tailed paired student’s t-test. The P-values in Additional File 7 Figure A was calculated using a two-tailed paired student’s t-test for the “pairs” plot, and a two-tailed un-paired student’s t-test for the “All” plot. The P-values in Additional File 7 Figure E-L was calculated using a two-tailed un-paired student’s t-test.

## References

1. Griffiths-Jones S: **miRBase: the microRNA sequence database**. *Methods in molecular biology* 2006, **342**:129-138.

2. Baskerville S, Bartel DP: **Microarray profiling of microRNAs reveals frequent coexpression with neighboring miRNAs and host genes**. *Rna* 2005, **11**(3):241-247.

3. Meng WJ, Yang L, Ma Q, Zhang H, Adell G, Arbman G, Wang ZQ, Li Y, Zhou ZG, Sun XF: **MicroRNA Expression Profile Reveals miR-17-92 and miR-143-145 Cluster in Synchronous Colorectal Cancer**. *Medicine* 2015, **94**(32):e1297.

4. Arndt GM, Dossey L, Cullen LM, Lai A, Druker R, Eisbacher M, Zhang C, Tran N, Fan H, Retzlaff K *et al*: **Characterization of global microRNA expression reveals oncogenic potential of miR-145 in metastatic colorectal cancer**. *BMC cancer* 2009, **9**:374.

5. Hayashita Y, Osada H, Tatematsu Y, Yamada H, Yanagisawa K, Tomida S, Yatabe Y, Kawahara K, Sekido Y, Takahashi T: **A polycistronic microRNA cluster, miR-17-92, is overexpressed in human lung cancers and enhances cell proliferation**. *Cancer research* 2005, **65**(21):9628-9632.

6. Menigatti M, Staiano T, Manser CN, Bauerfeind P, Komljenovic A, Robinson M, Jiricny J, Buffoli F, Marra G: **Epigenetic silencing of monoallelically methylated miRNA loci in precancerous colorectal lesions**. *Oncogenesis* 2013, **2**:e56.

7. Chen WS, Leung CM, Pan HW, Hu LY, Li SC, Ho MR, Tsai KW: **Silencing of miR-1-1 and miR-133a-2 cluster expression by DNA hypermethylation in colorectal cancer**. *Oncology reports* 2012, **28**(3):1069-1076.

8. Andrews S: **FastQC: A quality control tool for high throughput sequencing data**. 2010.

9. Martin M: **Cutadapt-1.10.** [**https://cutadapt.readthedocs.org/**](https://cutadapt.readthedocs.org/)**. http//dx.doi.org/10.14806/ej.17.1.200**.

10. Anders S, Pyl PT, Huber W: **HTSeq--a Python framework to work with high-throughput sequencing data**. *Bioinformatics* 2015, **31**(2):166-169.

11. Griffiths-Jones S, Saini HK, van Dongen S, Enright AJ: **miRBase: tools for microRNA genomics**. *Nucleic acids research* 2008, **36**(Database issue):D154-158.

12. Pantano L, Estivill X, Marti E: **SeqBuster, a bioinformatic tool for the processing and analysis of small RNAs datasets, reveals ubiquitous miRNA modifications in human embryonic cells**. *Nucleic Acids Res* 2010, **38**(5):e34.

13. Law CW, Chen Y, Shi W, Smyth GK: **voom: Precision weights unlock linear model analysis tools for RNA-seq read counts**. *Genome Biol* 2014, **15**(2):R29.

14. Ritchie ME, Phipson B, Wu D, Hu Y, Law CW, Shi W, Smyth GK: **limma powers differential expression analyses for RNA-sequencing and microarray studies**. *Nucleic acids research* 2015, **43**(7):e47.
