# Supplementary material for: Small RNA expression from viruses, bacteria and human miRNAs in colon cancer tissue and its association with microsatellite instability and tumor location
Source: BMC Cancer. 2019 Feb 20;19:161. doi: 10.1186/s12885-019-5330-0 (PMC6381638; doi:10.1186/s12885-019-5330-0)
Supplement: Supplementary file 3 — Figure S1. Sequencing statistics. A) Library size: Number of raw reads in the samples; Alignment: Number of reads that aligned to only one position in the genome (SingleAligned), multiple positions (MultiAligned) or not aligned (NotAligned); Features: Number of reads that aligned to miRBase, RNACentral database and to the calibrator RNAs; RNAs: Number of reads for the main RNA classes. B) Composition of RNAs in the samples shown as ratios. C) The sequences of the calibrator RNAs. (PDF 492 kb) [file 12885_2019_5330_MOESM3_ESM.pdf]

A

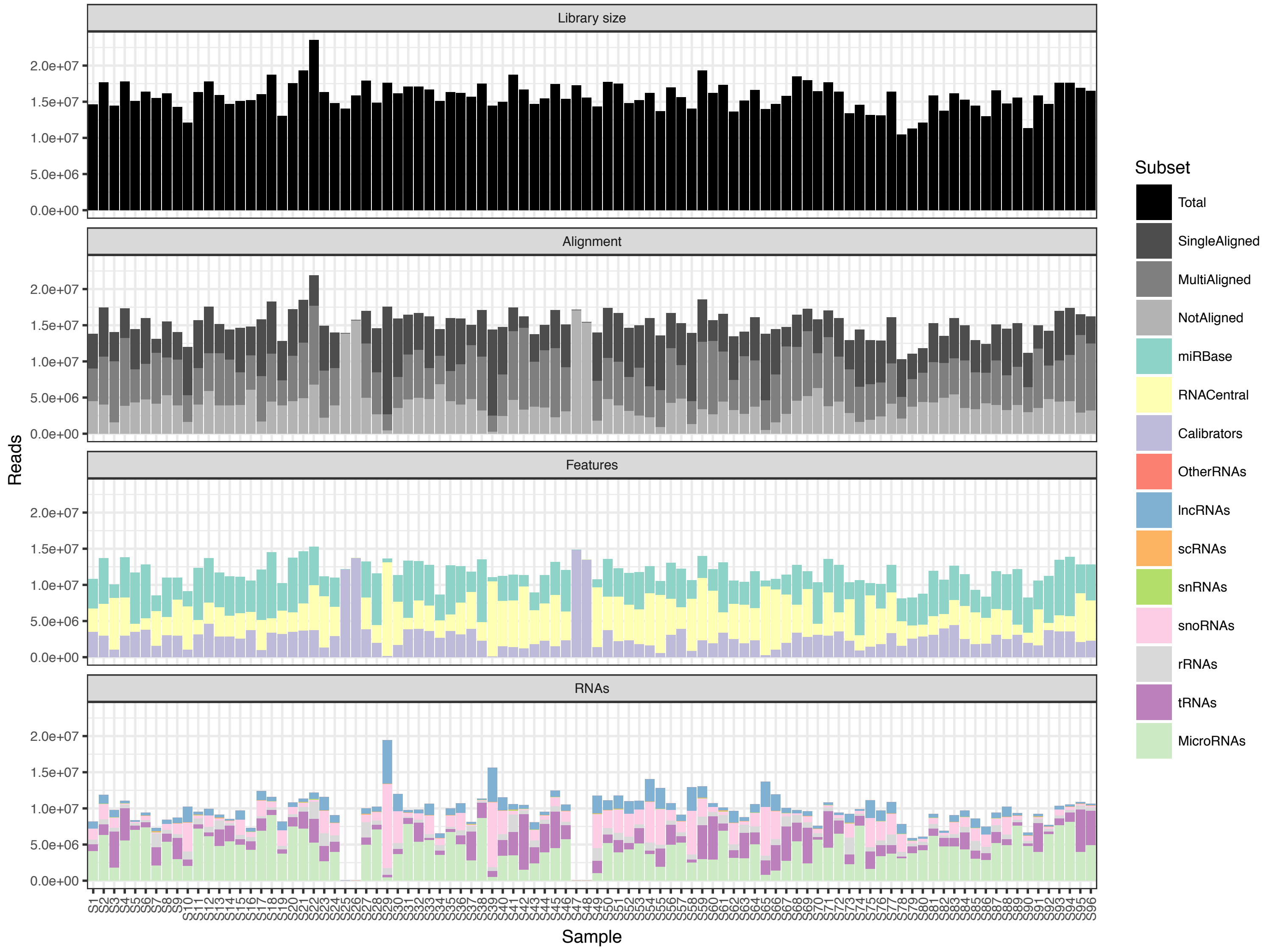

B

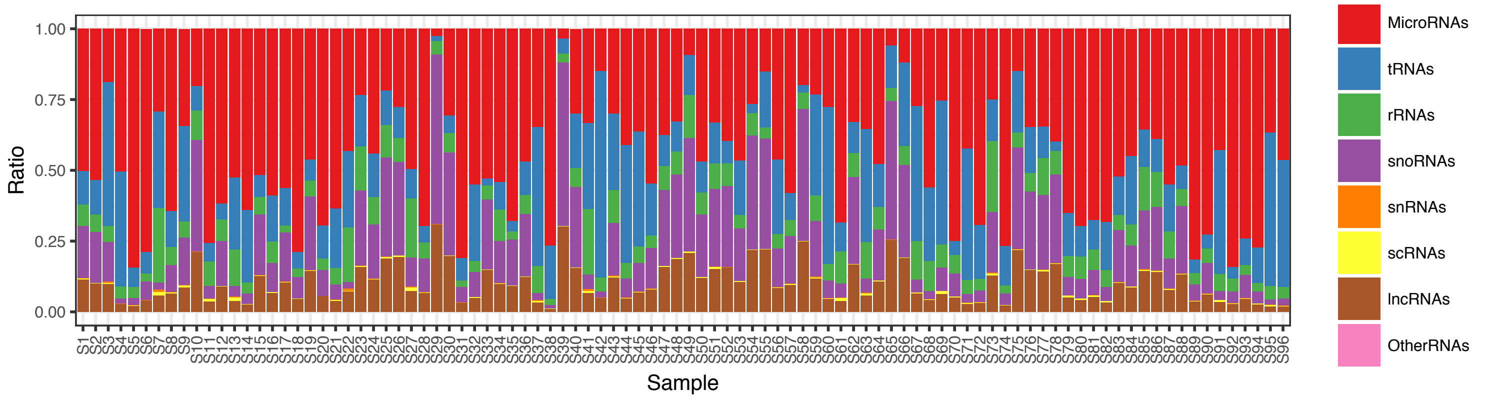

C

Calibrator ID	Sequence
Cal01	GTCCCACTCCGTAGATCTGTTC
Cal02	GATGTAACGAGTTGGAATGCAA
Cal03	TAGCATATCGAGCCTGAGAACA
Cal04	CATCGGTCGAACTTATGTGAAA
Cal05	GAAGCACATTCGCACATCATAT
Cal06	TCTTAACCCGGACCAGAAACTA
Cal07	AGGTTCCGGATAAGTAAGAGCC
Cal08	TAATCTCCTTAAGCGAATCTCGC
Cal09	AAAGTAGCATCCGAAATACGGA
Cal10	TGATACGGATGTTATACGCAGC
