# Supplementary material for: Small RNA expression from viruses, bacteria and human miRNAs in colon cancer tissue and its association with microsatellite instability and tumor location
Source: BMC Cancer. 2019 Feb 20;19:161. doi: 10.1186/s12885-019-5330-0 (PMC6381638; doi:10.1186/s12885-019-5330-0)

A

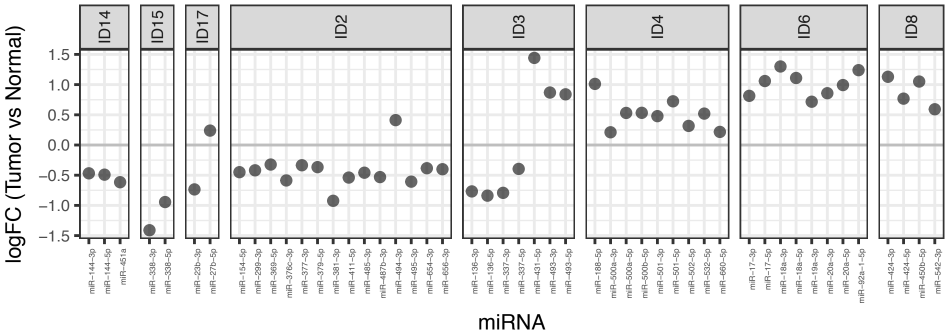

B

3' non-tempated addition TACCCTGTAGAACCGAATTTGTGTA
3' tailing TACCCTGTAGAACCGAATTTGTGTG
3' trimming TACCCTGTAGAACCGAATTTGTG
5' tailing ATACCCTGTAGAACCGAATTTGTGT
5' trimming ACCCTGTAGAACCGAATTTGTGT
canonical hsa-miR-10b-5p TACCCTGTAGAACCGAATTTGTGT
precursor hsa-miR-10b-5p TATATACCCTGTAGAACCGAATTTGTGTGGTATCCGTATAGTCACAGATTCGATTCTAGGGGAATATA

C

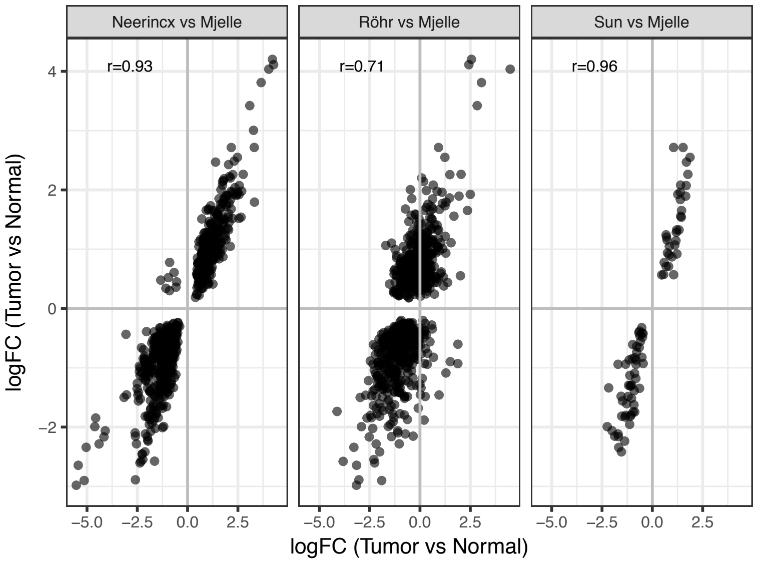

Supplement: Supplementary file 4 — Figure S2. MicroRNA clusters and isomiRs. A) A scatterplot of genomic clustered miRNAs showing log fold change values between tumor and normal (y-axis) for each individual miRNA within the clusters. The clusters are indicated with a unique ID on each facet of the plot. The figure only includes miRNAs that are significantly differentially expressed between tumor and normal. B) Illustrations of the main types of isomiRs analyzed in the current study, exemplified for hsa-miR-10b. C) Scatterplot comparing fold-change values for isomiRs between our dataset and the Neerincx, Röhr, and Sun datasets. (PDF 1751 kb) [file 12885_2019_5330_MOESM4_ESM.pdf]
