# Supplementary figures and images for: Small RNA expression from viruses, bacteria and human miRNAs in colon cancer tissue and its association with microsatellite instability and tumor location
Source: BMC Cancer. 2019 Feb 20;19:161. doi: 10.1186/s12885-019-5330-0 (PMC6381638; doi:10.1186/s12885-019-5330-0)

A

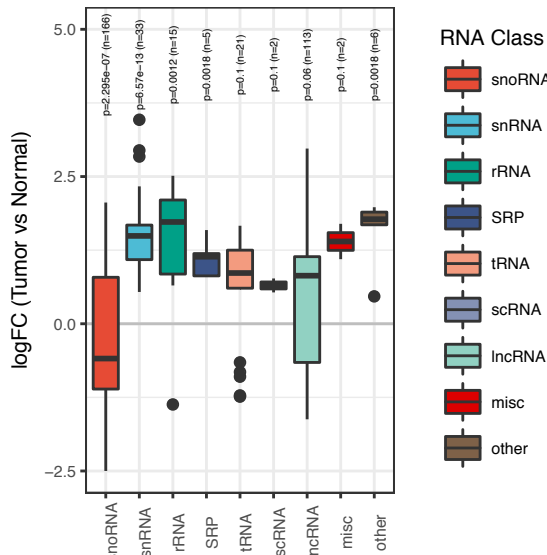

B

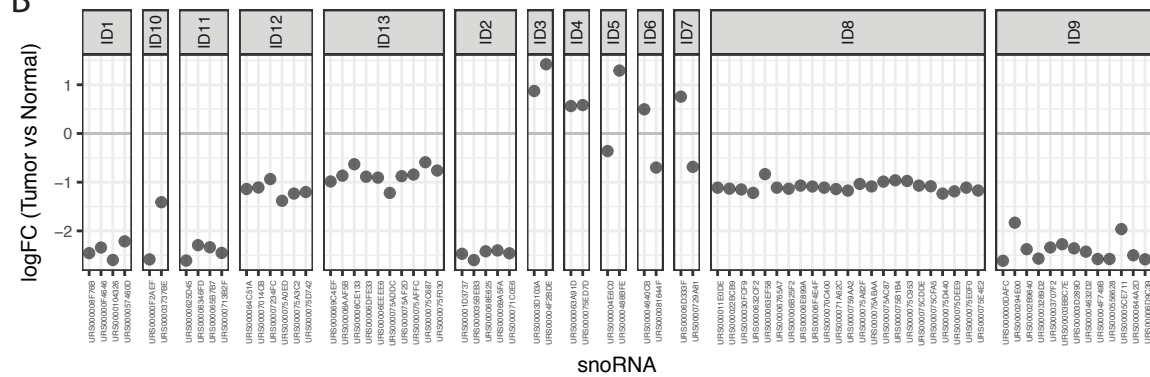

C

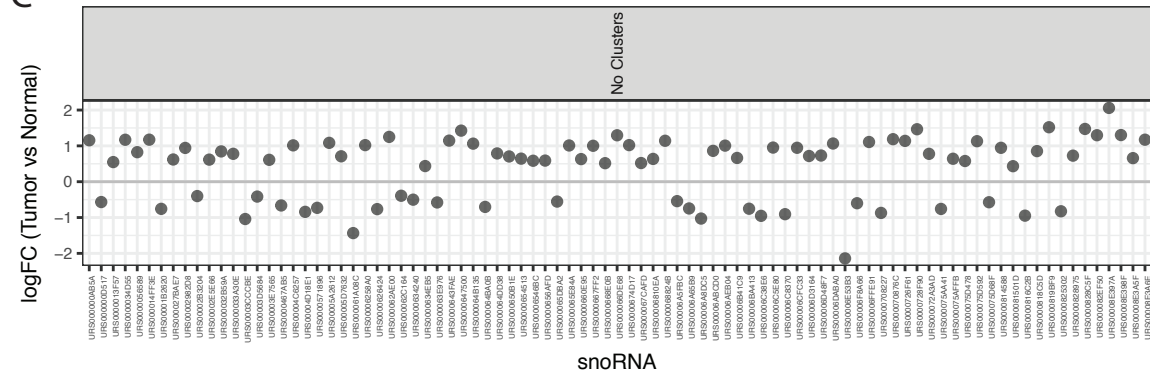

D

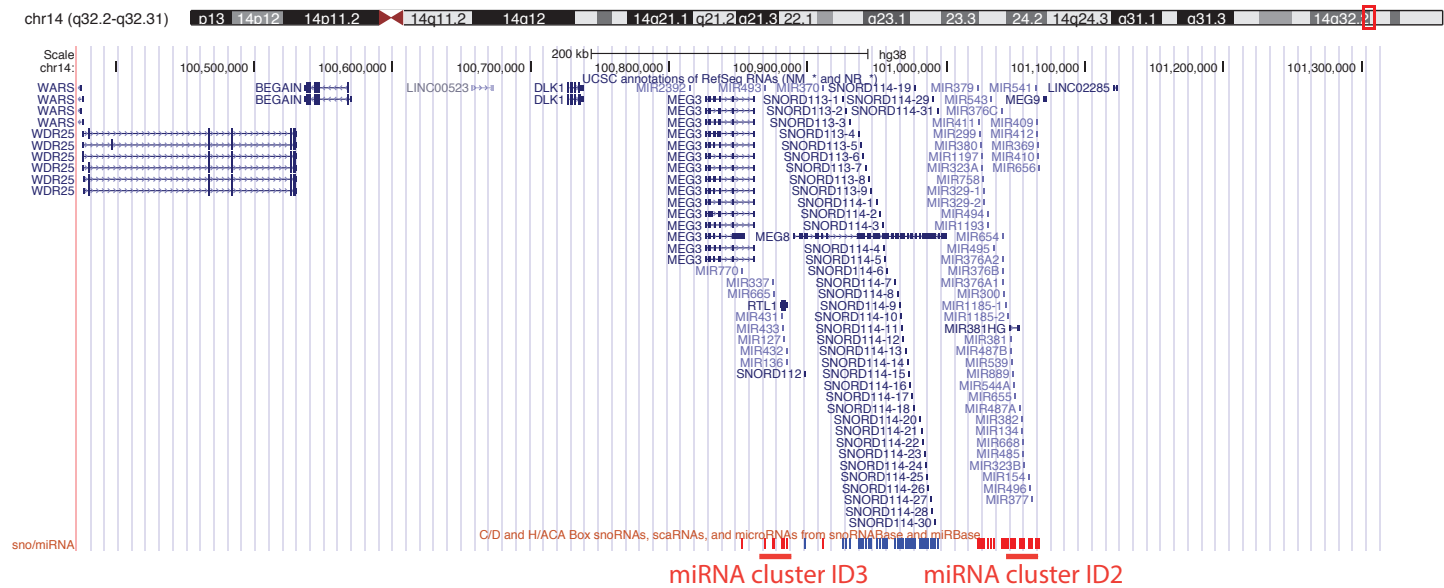

E

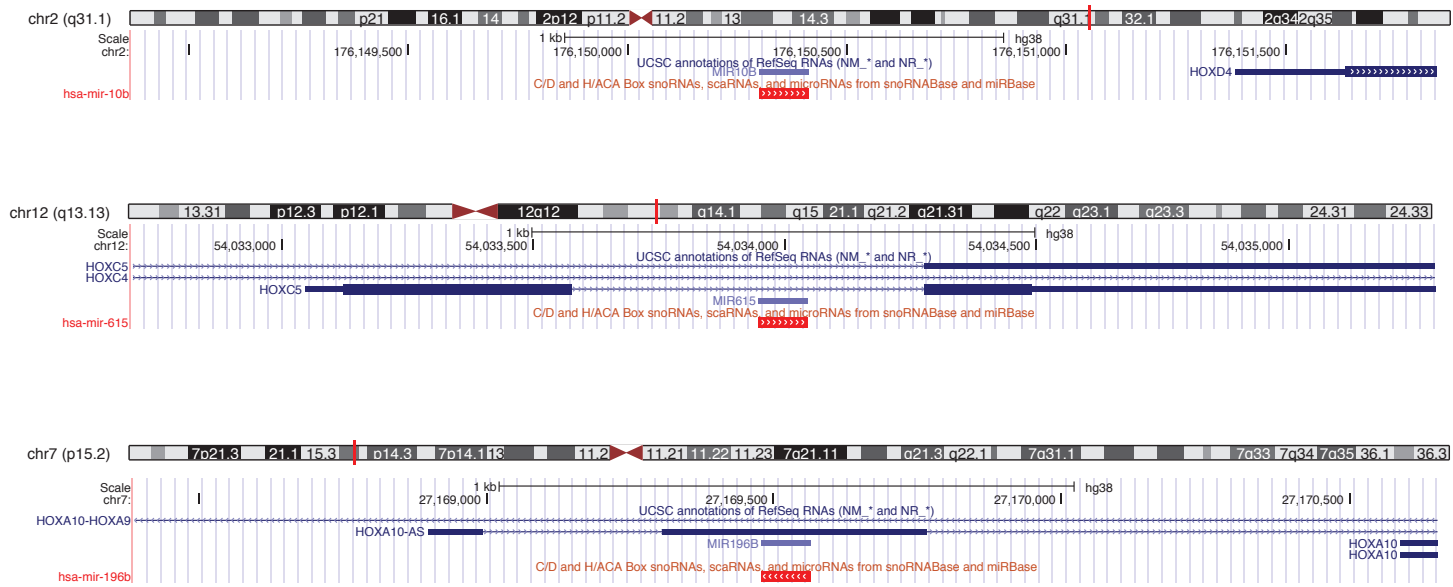

Supplement: Supplementary file 5 — Figure S3. Classes of ncRNAs and snoRNA clusters. A) Boxplot showing expression of the sRNAs that differ significantly between tumor and normal samples, grouped by RNA classes. B) A scatterplot of genomic clustered snoRNAs showing log fold change values between tumor and normal (y-axis) for each individual snoRNA within the clusters. The clusters are indicated with a unique ID on each facet of the plot. The figure only includes snoRNAs that are significantly differentially expressed between tumor and normal. The p-values are calculated using a two-tailed student’s t-test. C) Log fold change values for snoRNAs that are not contained within a genomic cluster. D) Genome browser graphics of snoRNAs adjacent to the MEG3 gene. The graphics is from http://genome-euro.ucsc.edu. The miRNA clusters ID2 and ID3 are indicated with a red line below the graphics. E) Genome browser graphics of miR-10b, miR-615 and miR-196b and the adjacent RefSeq genes. (PDF 673 kb) [file 12885_2019_5330_MOESM5_ESM.pdf]

A

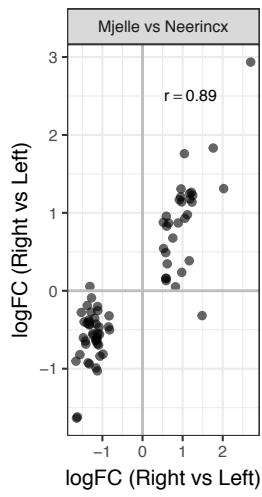

B

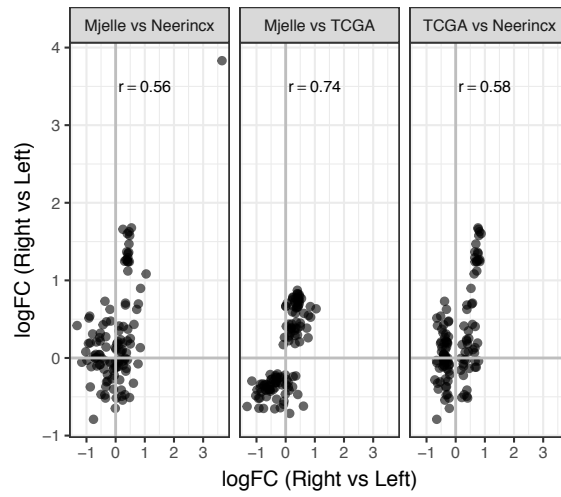

C

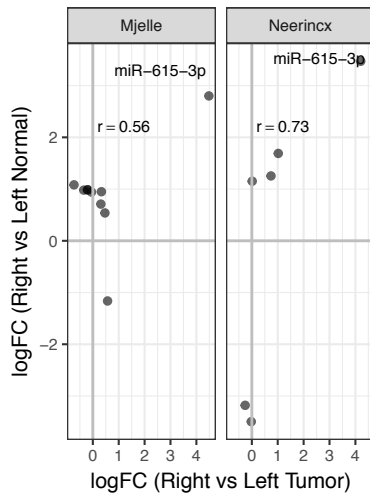

D

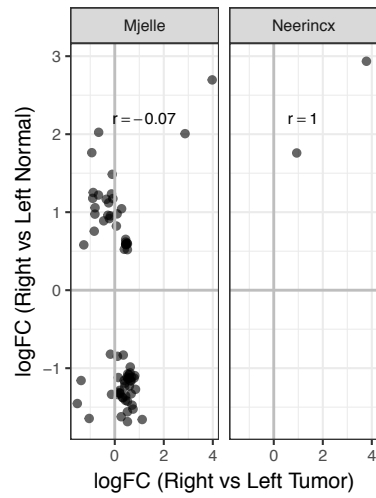

E

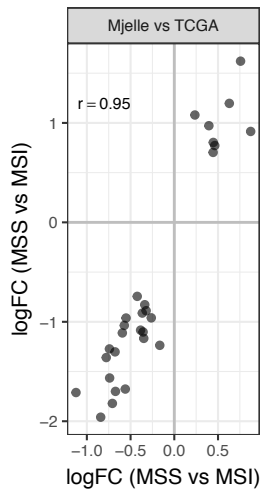

F

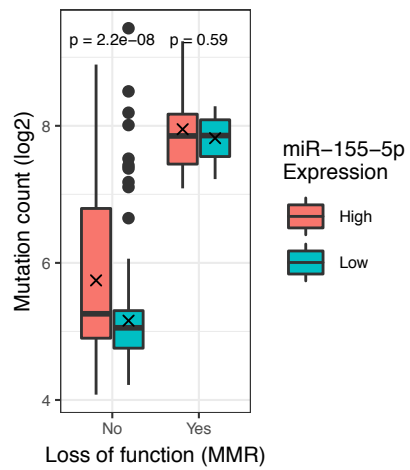

G

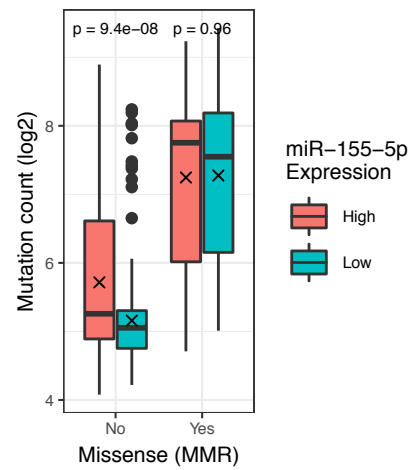

Supplement: Supplementary file 6 — Figure S4. MicroRNAs and isomiRs correlated with clinicopathological characteristics. A) Differentially expressed isomiRs in our dataset and the dataset of Neerincx et al. between right and left normal colon tissue. The comparison is Right-Left such that a positive fold change indicates that the corresponding miRNA is upregulated in right normal colon compared to left normal colon. B) Differentially expressed isomiRs in our data and the dataset of Neerincx et al. and TCGA comparing right and left tumor colon tissue. The statistical comparison is described in A). C) Differentially expressed miRNAs between right and left tumor tissue (x-axis) compared with differentially expressed miRNAs between right and left normal tissue (y-axis), for the datasets Mjelle et al. and Neerincx et al. D) Similar comparison as in C), for isomiRs. E) Differentially expressed isomiRs in our data and the dataset and TCGA comparing MSS and MSI. The comparison is MSS-MSI such that a positive fold change indicates that the corresponding isomiR is upregulated in MSS compared to MSI. F-G) Mutation counts for tumors grouped by miR-155 expression level and presence of somatic (F) loss-of-function or (G) missense mutations in MMR genes. The mean value is indicated with a cross. The p-values is the output from the linear model. (PDF 1150 kb) [file 12885_2019_5330_MOESM6_ESM.pdf]

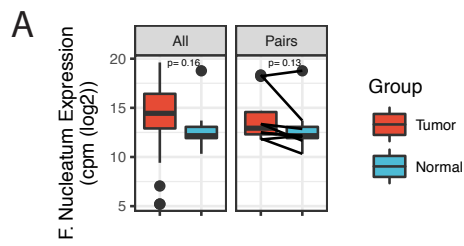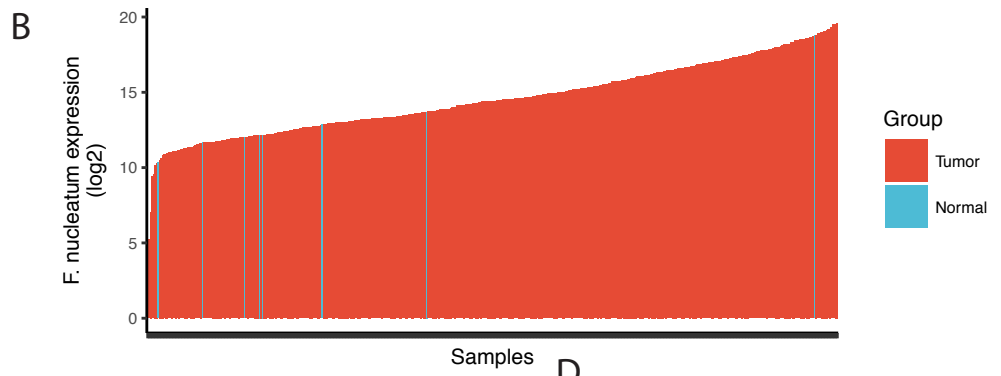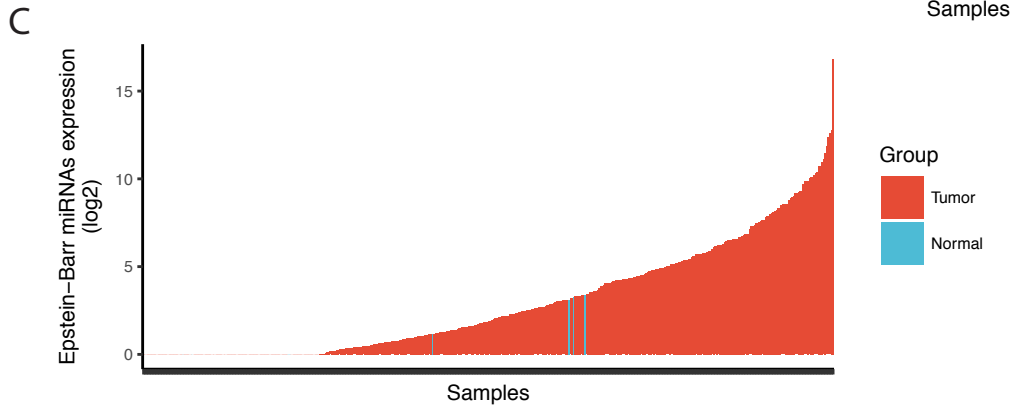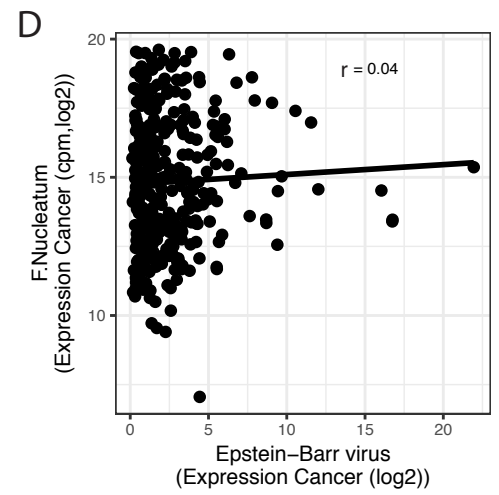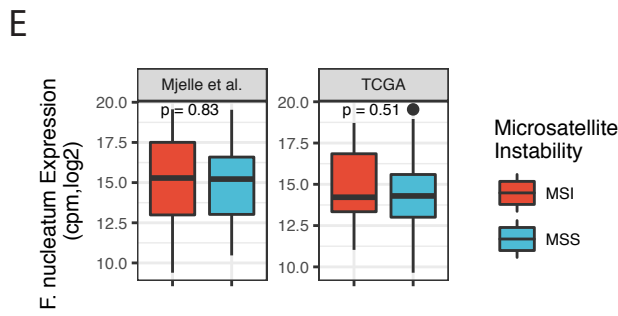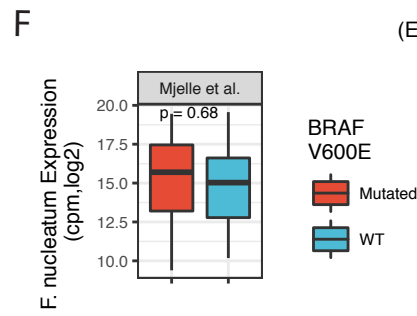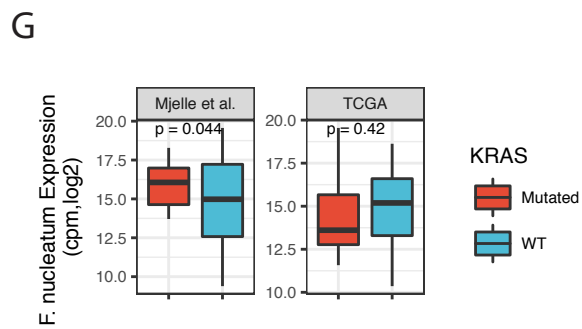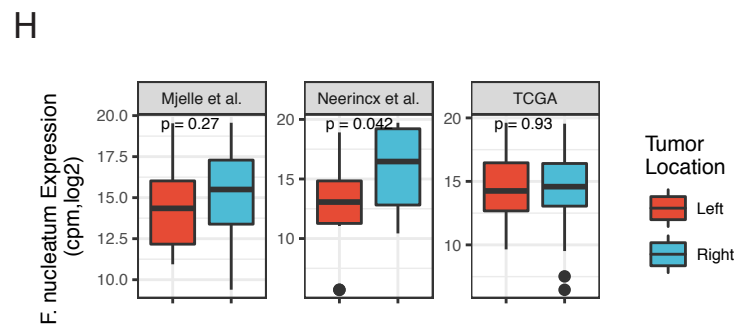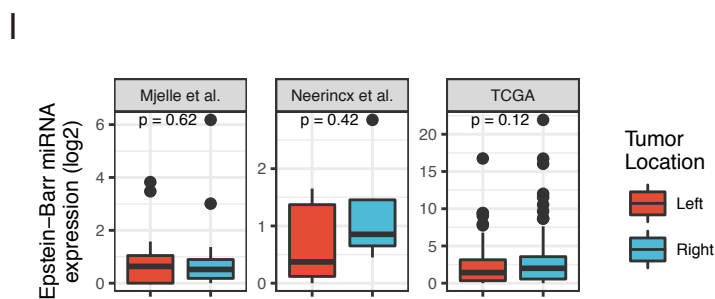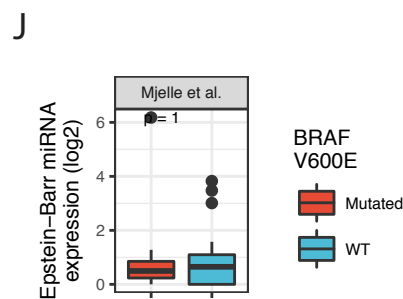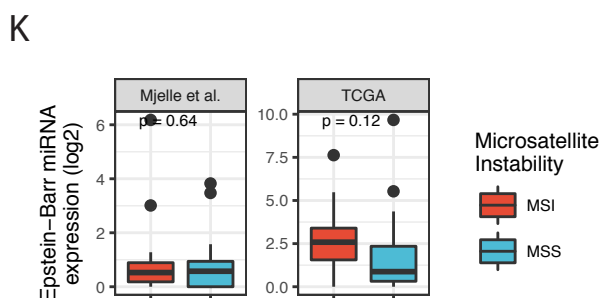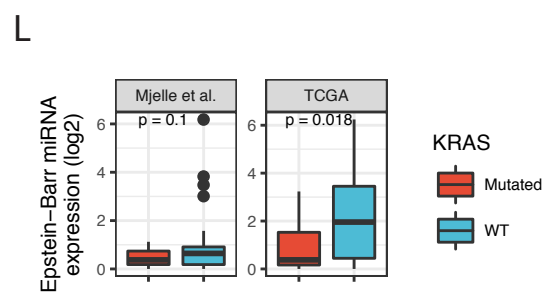

Supplement: Supplementary file 7 — Figure S5. Expression of F. nucleatum and EBV miRNA in TCGA data and correlation with clinical parameters across data sets. The p-values in Fig. S5E-L are calculated using an unpaired two-tailed Student’s t-test. A) Expression of F. nucleatum in TCGA small RNA data. Left: All tumor samples compared to the eight normal samples. Right: The 8 paired tumor and normal samples. The p-values are calculated using an unpaired and paired two-tailed Student’s t-test for the left and right boxplot, respectively. B) Expression of F. nucleatum in TCGA colon samples. C) Expression of EBV miRNAs in TCGA colon samples. The figure shows the sum of all detected EBV miRNA for each sample as log2 and is normalized based on library sizes of mature miRNAs in the corresponding samples. D) Correlation between F. nucleatum and EBV miRNA in Neerincx et al., Sun et al., TCGA, and our datasets. The correlation is calculated using the cor() function in R with pearson correlation. E) F. nucleatum expression in MSI and MSS tumors in Mjelle et al. and TCGA. F) F. nucleatum expression in BRAF mutated and BRAF wild type (non-mutated) tumors in Mjelle et al. G) F. nucleatum expression in KRAS mutated and KRAS wild-type (non-mutated) tumors in Mjelle et al. and TCGA. H) F. nucleatum expression in right-and left-sided tumor tissue in Mjelle et al., Neerincx et al. and TCGA. I) EBV miRNA expression in right-and left-sided tumor tissue in Mjelle et al., Neerincx et al. and TCGA. J) EBV miRNA in BRAF mutated and BRAF wild type (non-mutated) tumors in Mjelle et al. K) EBV miRNA expression in MSI and MSS tumors in Mjelle et al. and TCGA. L) EBV miRNA expression in KRAS mutated and KRAS wild-type (non-mutated) tumors in Mjelle et al. and TCGA. (PDF 462 kb) [file 12885_2019_5330_MOESM7_ESM.pdf]
